# NRIP1 co-activates nuclear translocated FOXO3 to upregulate TFAM expression and promote radioresistance in non-small cell lung cancer

**DOI:** 10.1038/s41420-026-03028-8

**Published:** 2026-03-27

**Authors:** Ying Zha, Hui Huang, Yulian Liu, Mengzhi Wan, Qinqin Yao, Hongtao Chen, Jianping Xiong, Min Zhong

**Affiliations:** 1 Department of Oncology, The First Affiliated Hospital, Jiangxi Medical College, Nanchang University, Nanchang, PR China; 2 Jiangxi Key Laboratory for Individual Cancer Therapy, Nanchang, PR China; 3 Department of Respiratory Emergency and Critical Care Medicine, The First Affiliated Hospital of Nanchang University, Nanchang, PR China

**Keywords:** Non-small-cell lung cancer, Radiotherapy, Nuclear receptors

## Abstract

Radioresistance remains a major obstacle in the treatment of non-small cell lung cancer (NSCLC). This study investigated the coordinated regulation of TFAM, FOXO3, and NRIP1 in NSCLC radioresistance. Radioresistant cell lines (A549-RR and H157-RR) were established to examine the effects of silencing these factors on cellular responses to radiation. In vivo, the impact of FOXO3 knockdown on tumor growth under irradiation was evaluated using A549-RR xenografts. Results show that TFAM expression was elevated in radioresistant cells, and its knockdown significantly restored radiosensitivity. ChIP-qPCR demonstrated direct FOXO3 binding to TFAM regulatory regions, establishing FOXO3 as an upstream transcriptional activator of TFAM. Silencing FOXO3 reduced TFAM expression and enhanced radiosensitivity, whereas LOM612, a FOXO nuclear relocator, promoted FOXO3 nuclear accumulation, upregulated TFAM, and reduced radiosensitivity. NRIP1 deficiency constrains FOXO3-dependent regulation of TFAM. Restoring NRIP1 selectively enhanced TFAM without affecting FOXO3 abundance, indicating its role as a coactivator. Co-immunoprecipitation confirmed FOXO3/NRIP1 interaction in NSCLC cells, with stronger interactions observed in radioresistant cells. Accordingly, NRIP1 silencing decreased TFAM levels and increased radiosensitivity. In vivo, FOXO3 knockdown markedly suppressed A549-RR tumor growth and improved radiotherapy response. Collectively, these findings indicate that nuclear accumulation of FOXO3 drives NSCLC radioresistance by transcriptionally upregulating TFAM, with NRIP1 enhancing this regulatory activity. Targeting FOXO3 may represent a promising strategy to enhance radiosensitivity in NSCLC.

## Introduction

Non-small cell lung cancer (NSCLC) is among the most common and deadly forms of lung cancer worldwide. Radiotherapy is a cornerstone treatment, particularly for patients with locally advanced or inoperable disease; however, its effectiveness is frequently limited by the emergence of radioresistance [1]. Radioresistance is characterized by a reduced susceptibility of tumor cells to the cytotoxic effects of ionizing radiation, including DNA damage, oxidative stress, and apoptosis, which ultimately contributes to treatment failure and disease progression [2]. The mechanisms driving NSCLC radioresistance are complex and multifactorial. A deeper understanding of these processes and the development of targeted combination therapies may provide new strategies to overcome radioresistance and improve clinical outcomes [3].

The antitumor effects of radiotherapy are largely mediated by the induction of reactive oxygen species (ROS) and DNA damage, with mitochondrial function playing a central role in regulating ROS homeostasis, metabolic reprogramming, and apoptosis [4]. Mitochondrial dynamics—including fission, fusion, and mitophagy—further influence cell survival following radiation exposure [5]. Consequently, mitochondrial dysfunction has emerged as a critical mechanism underlying NSCLC radioresistance [6]. Targeting mitochondrial pathways, therefore, represents a promising strategy to enhance radiosensitivity in NSCLC.

Mitochondria contain their own DNA encoding a limited set of proteins essential for oxidative phosphorylation; however, the majority of mitochondrial proteins ( ~ 2000) are nuclear-encoded. Among these, mitochondrial transcription factor A (TFAM) is indispensable for mitochondrial DNA replication, transcription, maintenance, and biogenesis [7]. Our preliminary data show that TFAM expression is markedly elevated in radioresistant NSCLC cells, accompanied by increased mitochondrial DNA content, and that TFAM knockdown restores radiosensitivity, suggesting a pivotal role in radioresistance.

To explore upstream regulators of TFAM, we analyzed its coding sequence and identified multiple consensus Forkhead box O3 (FOXO3) binding motifs (5’-[AG]TAAA[TC]A-3’), raising the possibility that the transcription factor FOXO3 directly controls TFAM expression. FOXO3, a member of the FoxO family, regulates cell fate decisions in response to stress by cooperating with co-regulators. It also serves as a key modulator of mitochondrial homeostasis, influencing mitochondrial dynamics and mitophagy [8, 9]. Under oxidative stress, FOXO3 translocates to the nucleus, where it binds promoter regions of antioxidant genes to activate their transcription [10]. Because radiotherapy generates substantial ROS, inducing oxidative stress in tumor cells [11], we hypothesize that FOXO3 translocates to the nucleus upon radiation and upregulates TFAM to preserve mitochondrial integrity and promote radioresistance in NSCLC.

Furthermore, Nuclear Receptor Interacting Protein 1 (NRIP1), also known as RIP140, is a well-characterized transcriptional coregulator that modulates a broad spectrum of transcription factors in cancer [12]. Our preliminary data indicate that NRIP1 deficiency disrupts FOXO3-dependent regulation of downstream targets, suggesting that NRIP1 may act as a co-regulatory factor (potentially a co-activator) for FOXO3 in NSCLC.

In this study, we systematically investigate the role of the FOXO3/TFAM axis in NSCLC radioresistance using comprehensive in vitro and in vivo models. We further examine the contribution of NRIP1 to FOXO3-mediated TFAM transcription, aiming to uncover potential targets for enhancing radiosensitivity in NSCLC.

## Results

### TFAM is upregulated in radiation-resistant NSCLC cells

**Fig. 1:**
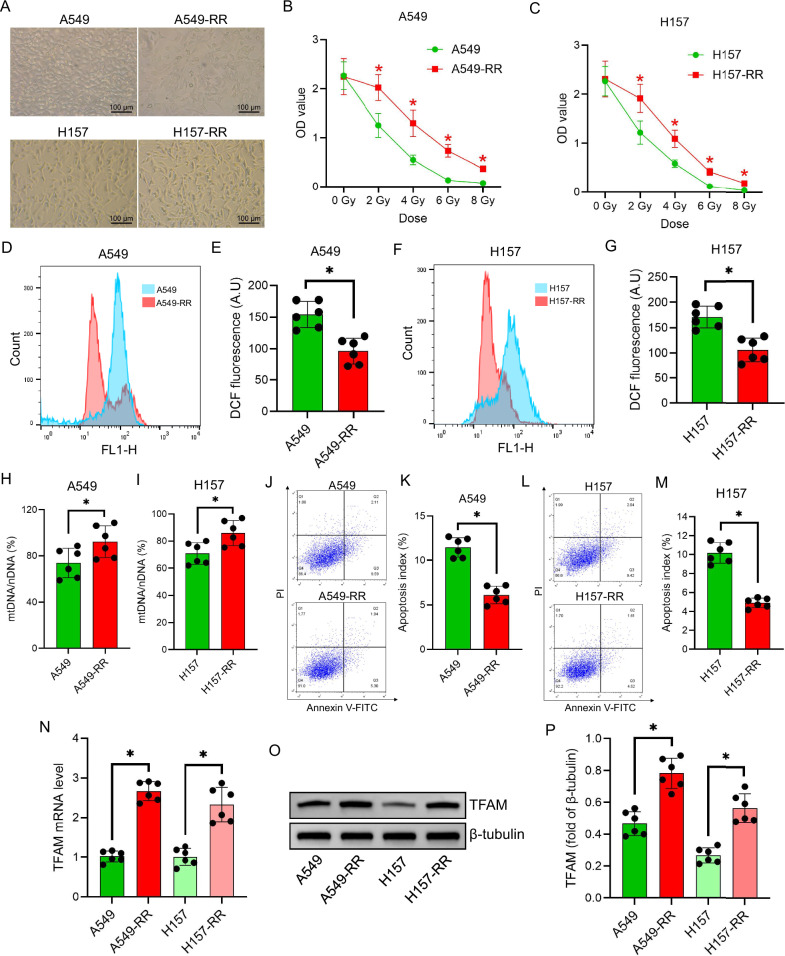
Establishment of radioresistant NSCLC cell lines and their impact on TFAM expression. **A** Morphological comparison between parental NSCLC cells and their radioresistant counterparts. **B**,**C** Cell survival of A549/A549-RR and H157/H157-RR cells following single-dose X-ray irradiation at varying doses. **D–G** Intracellular ROS levels in A549/A549-RR and H157/H157-RR cells 24 hours after 6 Gy irradiation, measured by flow cytometry. **H**,**I** mtDNA content in A549/A549-RR and H157/H157-RR cells following irradiation, quantified by qPCR. **J–M** Apoptotic cell populations in A549/A549-RR and H157/H157-RR cells post-irradiation, analyzed by flow cytometry. **N** Relative TFAM mRNA expression in parental and radioresistant NSCLC cells after irradiation, determined by qPCR. **O**,**P** TFAM protein levels in parental and radioresistant NSCLC cells post-irradiation, assessed by Western blotting. Data are presented as mean ± SD (*n* = 6). ^*^P < 0.05.

Radiation-resistant NSCLC cell lines A549-RR and H157-RR exhibited distinct morphological changes, including cell elongation and prominent filopodia (Fig. 1A). Upon irradiation, these resistant cells maintained significantly higher viability than their parental counterparts (*P* < 0.05; Fig. 1B, C), indicating reduced sensitivity to radiation-induced cytotoxicity.

We next assessed intracellular ROS levels. Compared with parental cells, radiation-resistant cells showed markedly lower ROS accumulation following irradiation (*P* < 0.05; Fig. 1D–G). In parallel, mtDNA copy number was significantly higher in resistant cells relative to normal cells after irradiation (*P* < 0.05; Fig. 1H, I).

Flow cytometry analysis further revealed a substantially lower apoptosis rate in resistant cells under identical radiation conditions (*P* < 0.05; Fig. 1J–M), confirming the successful establishment of radiation resistance.

To explore underlying molecular alterations, we evaluated TFAM expression. qPCR analysis demonstrated a significant upregulation of TFAM mRNA in resistant cells (*P* < 0.05; Fig. 1N), which was corroborated by Western blotting showing increased TFAM protein levels (*P* < 0.05; Fig. 1O, P).

### TFAM knockdown sensitizes resistant NSCLC cells to irradiation

**Fig. 2:**
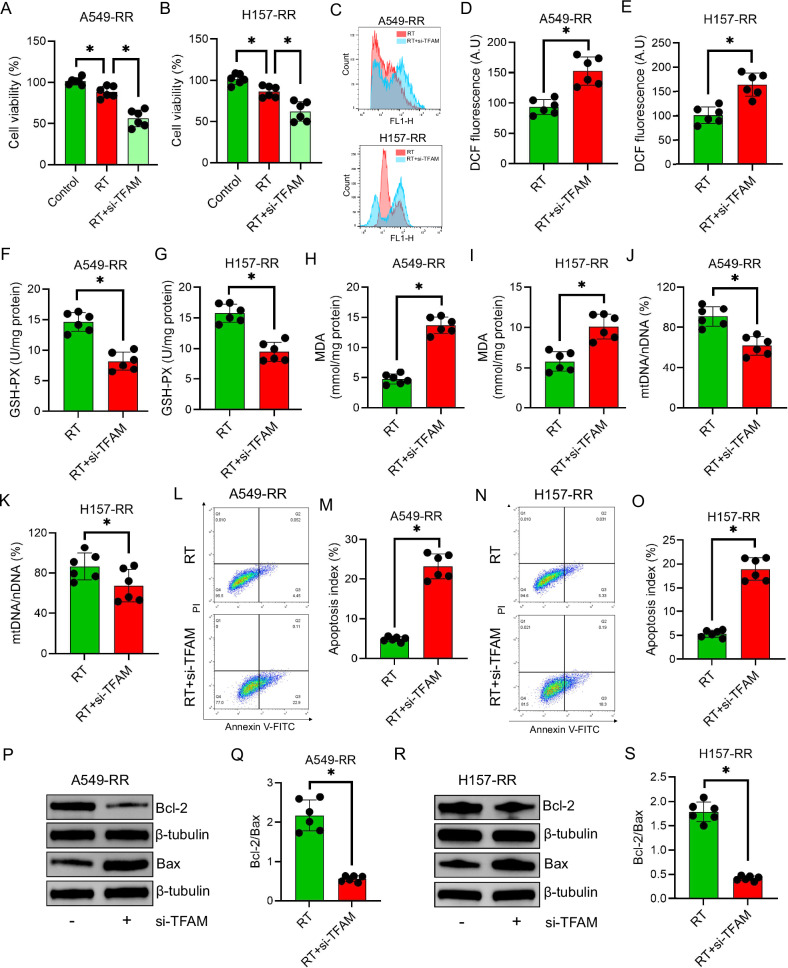
Effects of TFAM knockdown on radioresistant NSCLC cells. **A**,**B** Cell viability of A549-RR and H157-RR cells 24 h after a single 6 Gy X-ray exposure. The Control group represents non-irradiated cells; the RT group represents cells subjected to a single irradiation; and the RT + si-TFAM group represents cells transfected with 50 nM TFAM siRNA followed by irradiation 48 h later. **C–E** Intracellular ROS levels in A549-RR and TFAM-knockdown A549-RR cells after irradiation, analyzed by flow cytometry. **F**,**G** GSH-PX levels in A549-RR and H157-RR cells with or without TFAM knockdown following irradiation. **H**,**I** MDA levels in A549-RR and H157-RR cells after irradiation, with or without TFAM knockdown. **J**,**K** mtDNA content in A549-RR and H157-RR cells post-irradiation, quantified by qPCR. **L–O** Apoptotic cell populations in A549-RR and H157-RR cells after irradiation, with or without TFAM knockdown, assessed by flow cytometry. **P–S** Bcl-2 and Bax protein expression in A549-RR and H157-RR cells post-irradiation, analyzed by Western blotting. Data are presented as mean ± SD (*n* = 6). ^*^P < 0.05.

To investigate the functional role of TFAM, siRNA-mediated knockdown was performed in radiation-resistant NSCLC cells. TFAM silencing significantly enhanced radiation-induced cytotoxicity, resulting in reduced cell viability compared with irradiation alone (*P* < 0.05; Fig. 2A, B).

Flow cytometry revealed that ROS levels were markedly elevated in the RT+siTFAM group relative to irradiation alone (*P* < 0.05; Fig. 2C–E). Consistently, TFAM knockdown decreased GSH-PX activity and increased MDA levels, indicating augmented oxidative stress (*P* < 0.05; Fig. 2F–I).

Mitochondrial DNA content was significantly reduced in TFAM-silenced cells under irradiation (*P* < 0.05; Fig. 2J, K). Apoptosis analysis showed a substantial increase in apoptotic cells following TFAM knockdown (*P* < 0.05; Fig. 2L–O). Western blot analysis further demonstrated a decreased Bcl-2/Bax ratio in the RT+siTFAM group (*P* < 0.05; Fig. 2P–S), confirming enhanced apoptotic signaling.

### FOXO3 exhibits increased nuclear localization in resistant cells

**Fig. 3:**
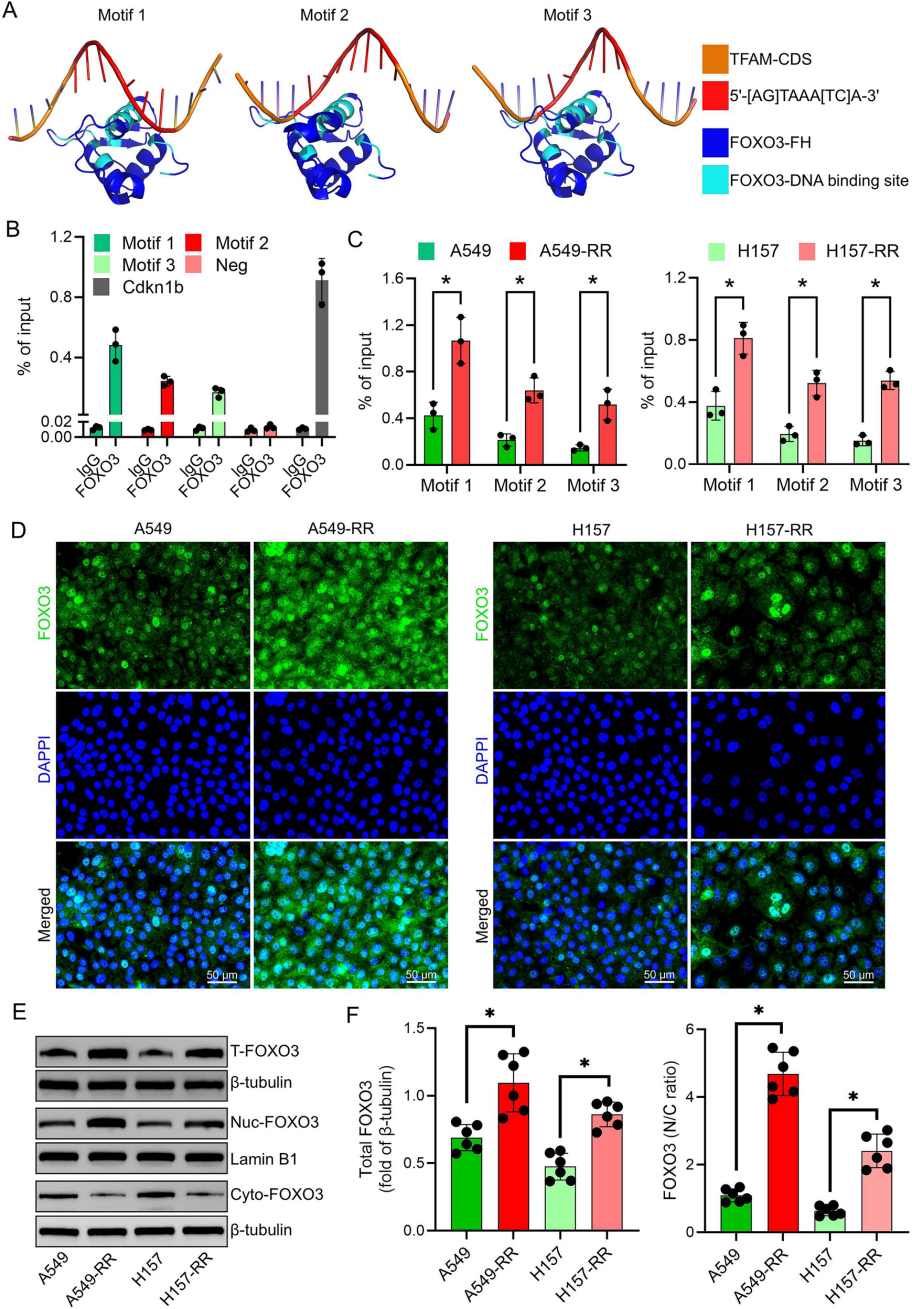
Effect of radioresistance on FOXO3 expression and nuclear localization in NSCLC cells. **A** AlphaFold simulation illustrating the interaction between the DNA-binding domain (DBD) of FOXO3 and three characteristic motifs of TFAM. Motif 1: 5’-TTACATAAACATCTC-3’, Motif 2: 5’-AGGTGTAAACAGTGA-3’, Motif 3: 5’-GCCAGTAAATAAAGT-3’. **B** ChIP-qPCR confirming the direct binding of FOXO3 to the regulatory regions of TFAM in A549 cells. **C** ChIP-qPCR analysis assessing the binding strength of FOXO3 to different TFAM motifs in NSCLC cells before and after the development of radioresistance. **D** Immunofluorescence analysis showing FOXO3 expression and subcellular localization in parental and radioresistant NSCLC cells. **E**,**F** Western blot analysis of total, nuclear, and cytoplasmic FOXO3 protein levels in normal and radioresistant NSCLC cells. Data are presented as mean ± SD (*n* = 6). ^*^P < 0.05.

Given the presence of FOXO3 consensus binding sites (5′-[AG]TAAA[TC]A-3′) within the TFAM coding sequence, we hypothesized that FOXO3 may regulate TFAM expression. AlphaFold simulations predicted interactions between FOXO3’s DNA-binding domain and these motifs (Fig. 3A). These predictions were experimentally validated by ChIP‑qPCR, which demonstrated that FOXO3 directly associates with the predicted motifs within the *TFAM* coding region, confirming the computationally identified binding sites (Fig. 3B, C).

Immunofluorescence analysis revealed markedly enhanced nuclear localization of FOXO3 in radiation-resistant cells compared with parental cells (Fig. 3D). Consistently, Western blotting confirmed increased total and nuclear FOXO3 protein levels in resistant cells (*P* < 0.05; Fig. 3E, F).

### FOXO3 knockdown reduces TFAM expression and enhances radiosensitivity

**Fig. 4:**
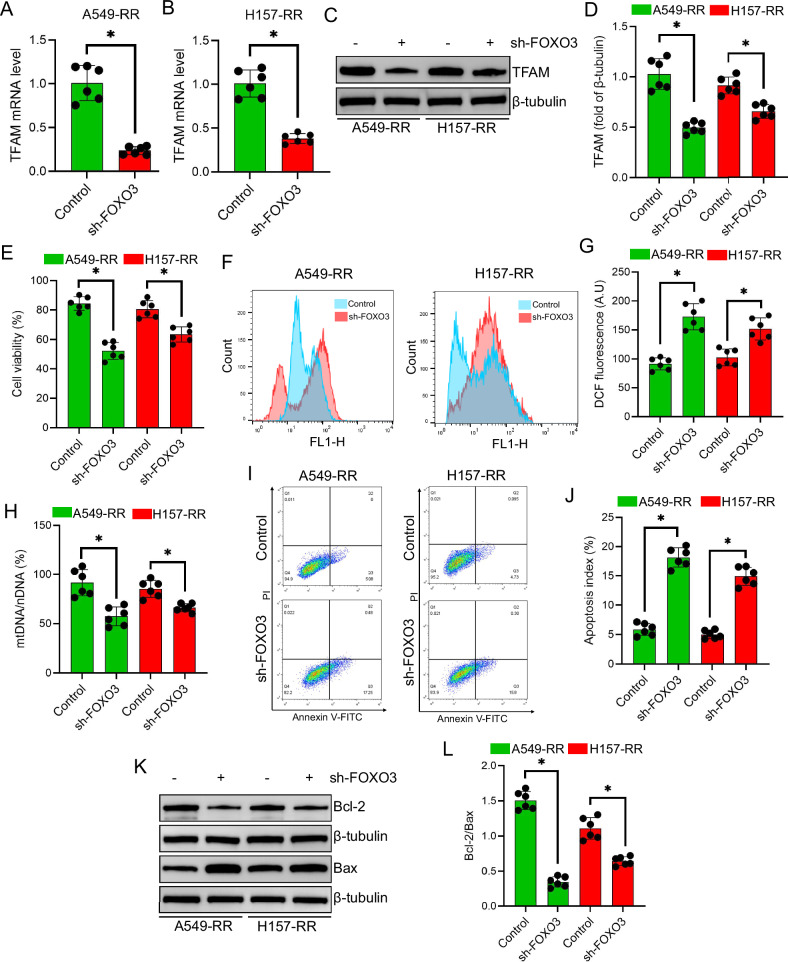
Effect of FOXO3 knockdown on radioresistant NSCLC cells. **A**,**B** Relative TFAM mRNA expression levels in radioresistant NSCLC cells after FOXO3 knockdown. **C**,**D** TFAM protein expression following FOXO3 knockdown in radioresistant NSCLC cells. **E** Cell viability of FOXO3-knockdown radioresistant NSCLC cells 24 h after a single 6 Gy irradiation. **F**,**G** Flow cytometry analysis of ROS levels in radioresistant NSCLC cells after FOXO3 knockdown and irradiation. **H** mtDNA levels in radioresistant NSCLC cells after FOXO3 knockdown, assessed by qPCR following irradiation. **I**,**J** Flow cytometry analysis of apoptosis in radioresistant NSCLC cells after FOXO3 knockdown post-irradiation. **K**,**L** Western blot analysis of Bcl-2 and Bax protein expression in radioresistant NSCLC cells after FOXO3 knockdown and irradiation. Data are presented as mean ± SD (*n* = 6). ^*^P < 0.05.

Silencing FOXO3 in A549-RR and H157-RR cells significantly decreased TFAM mRNA and protein expression (*P* < 0.05; Fig. 4A–D). FOXO3 knockdown also markedly reduced cell survival following irradiation (*P* < 0.05; Fig. 4E) and elevated intracellular ROS levels (*P* < 0.05; Fig. 4F, G). In addition, mtDNA content was significantly diminished in FOXO3-silenced cells under radiation (*P* < 0.05; Fig. 4H).

Consistent with these findings, apoptosis was significantly increased in FOXO3-deficient cells post-irradiation (*P* < 0.05; Fig. 4I, J), accompanied by a reduction in the Bcl-2/Bax ratio (*P* < 0.05; Fig. 4K, L), indicating enhanced radiation-induced apoptotic signaling.

### Nuclear accumulation of FOXO3 mediates TFAM activation and radiation resistance

**Fig. 5:**
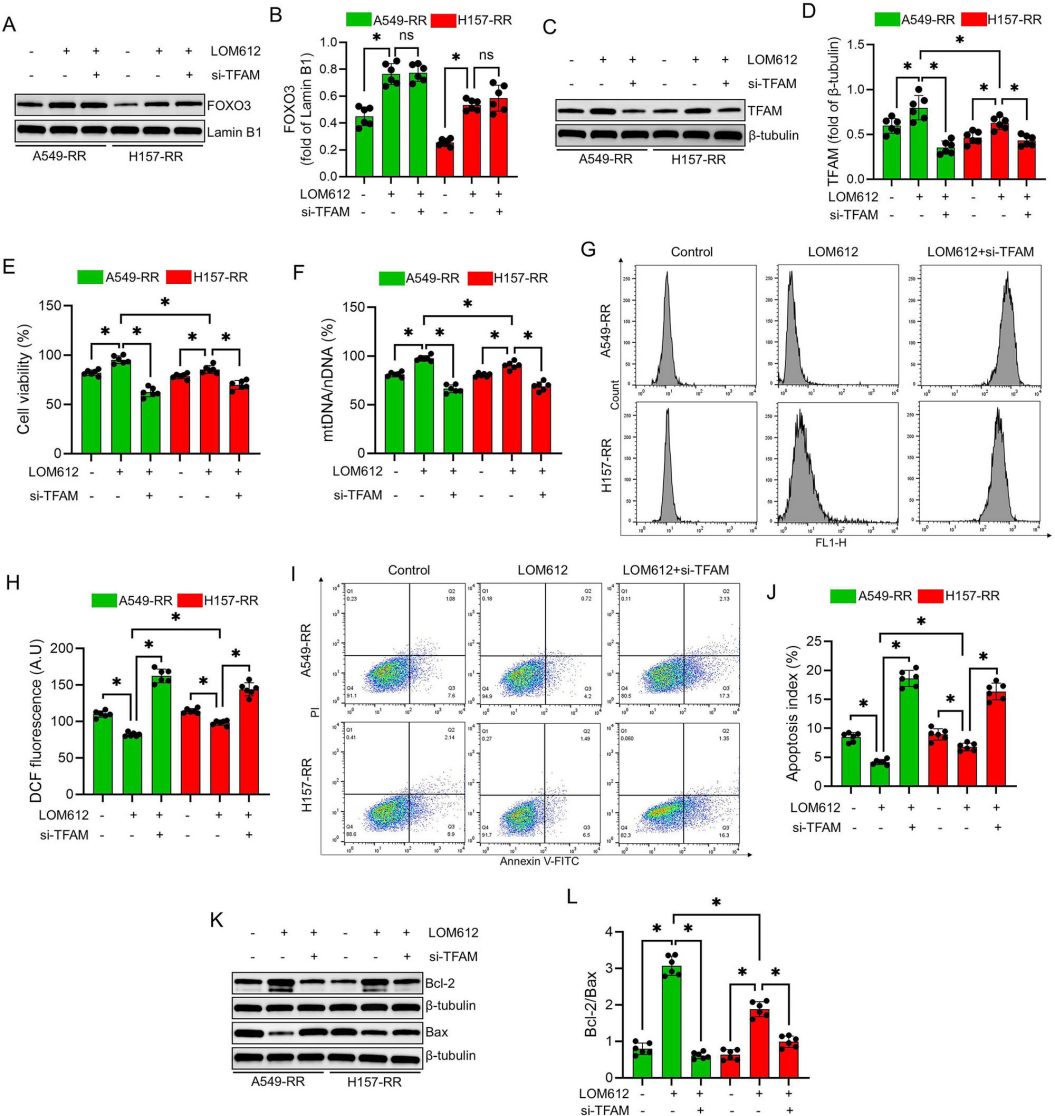
Effect of LOM612 on radioresistant NSCLC cells. **A**,**B** Western blot analysis showing FOXO3 protein expression in radiotherapy-resistant NSCLC cells 24 h after a single 6 Gy radiation dose. LOM612 (1 μM) was administered 24 h before irradiation. For si-TFAM treatment, cells were transfected with 50 nM siRNA using liposomes and irradiated 48 h later. When both interventions were combined, treatments followed the same schedule. All subsequent experimental groups adhered to this protocol. **C**,**D** Western blot analysis of TFAM protein expression under the same treatment conditions. **E** Assessment of cell viability in each group using the CCK-8 assay. **F** Quantification of mtDNA levels across treatment groups by qPCR. **G**,**H** Flow cytometric analysis of intracellular ROS levels in each group. **I**,**J** Flow cytometric evaluation of apoptosis in radioresistant NSCLC cells following irradiation treatment. **K**,**L** Western blot analysis of Bcl-2 and Bax protein expression in each group. Data are presented as mean ± SD (*n* = 6). ^*^P < 0.05.

Treatment with LOM612, a FOXO3 nuclear localization enhancer, significantly increased nuclear FOXO3 accumulation in radiation-resistant cells, regardless of TFAM knockdown (*P* < 0.05; Fig. 5A, B). LOM612 also upregulated TFAM expression (*P* < 0.05), an effect abolished when TFAM was silenced (Fig. 5C, D).

LOM612 enhanced cell survival and mtDNA content following irradiation, both of which were reversed by TFAM knockdown (*P* < 0.05; Fig. 5E, F). Moreover, LOM612 significantly decreased ROS accumulation and apoptosis, whereas TFAM silencing negated these effects (*P* < 0.05; Fig. 5G–J). Western blot analysis confirmed that LOM612 attenuated apoptotic signaling, as shown by an increased Bcl-2/Bax ratio, which was reversed upon TFAM knockdown (*P* < 0.05; Fig. 5K, L).

Notably, A549-RR cells exhibited a stronger response to LOM612 than H157-RR cells, with greater increases in TFAM expression, mtDNA content, and cell survival, along with more pronounced suppression of ROS and apoptosis (*P* < 0.05).

### NRIP1 stabilizes FOXO3 to promote TFAM transcription

**Fig. 6:**
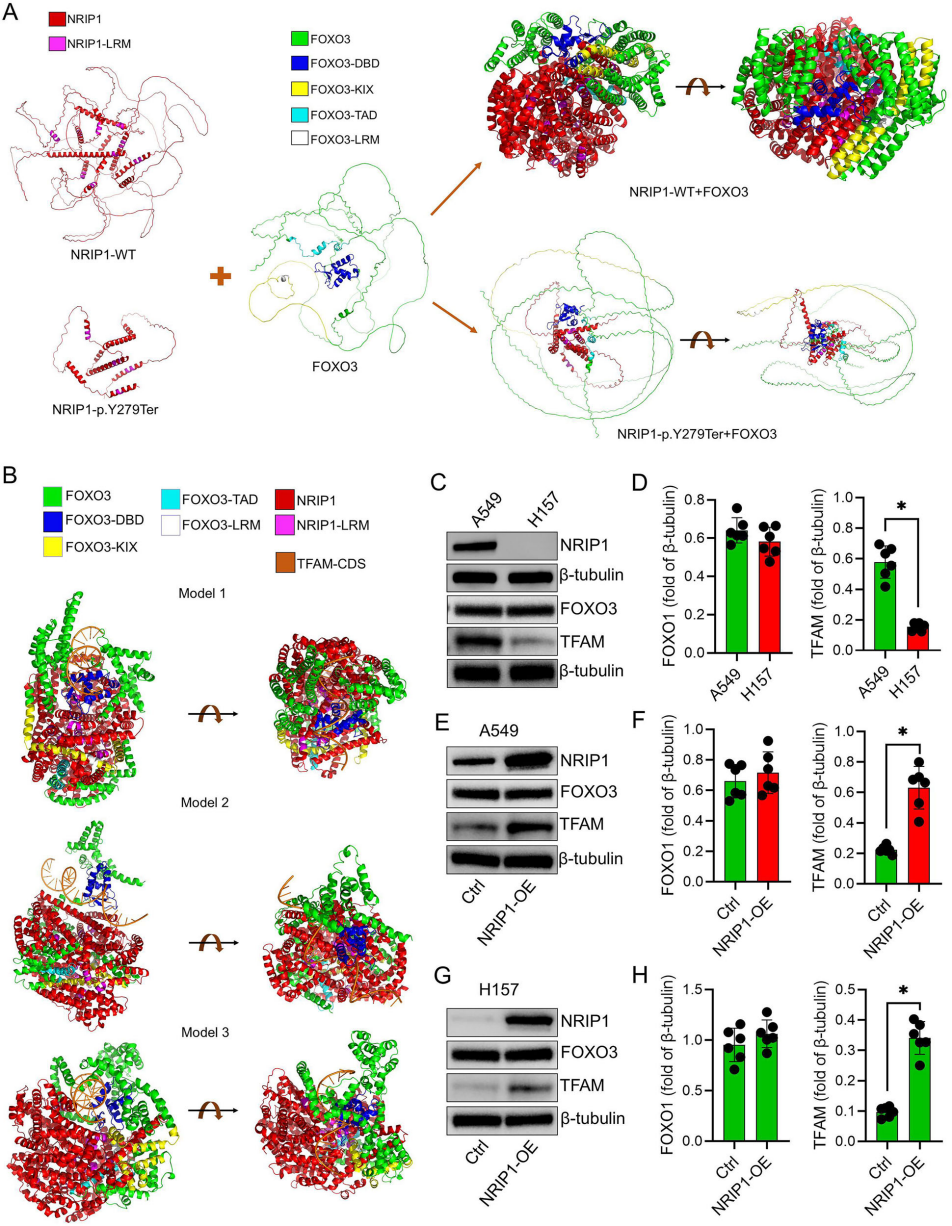
Simulation analysis of the NRIP1–FOXO3 protein interaction. **A** AlphaFold modeling of the interaction between FOXO3 and either wild-type or mutant NRIP1, illustrating predicted differences in binding conformation. **B** AlphaFold simulation of the binding patterns between the NRIP1-FOXO3 complex and characteristic TFAM sequences, revealing three distinct interaction modes. **C**,**D** Western blot analysis of NRIP1, FOXO3 and TFAM protein expression in A549 and H157 cells. **E**,**F** Western blot analysis of NRIP1, FOXO3 and TFAM protein expression in A549 cells after transfection of NRIP1 overexpression vector (NRIP1-OE) and empty control vector (Ctrl). **G**,**H** Western blot analysis of NRIP1, FOXO3 and TFAM protein expression in H157 cells after transfection of NRIP1 overexpression vector (NRIP1-OE) and empty control vector (Ctrl). Data are presented as mean ± SD (*n* = 6). ^*^P < 0.05.

To explore mechanisms underlying the differential responses of H157-RR and A549-RR cells to LOM612, mutational profiles were compared using the CCLE database, focusing on genes that could affect nuclear protein function or expression. This analysis identified NRIP1 as a candidate due to its known role in regulating nuclear transcription factors. H157 cells harbor a loss-of-function NRIP1 mutation (p.Y279Ter), generating a truncated protein prone to misfolding and rapid degradation, effectively eliminating NRIP1 expression (https://depmap.org/portal/cell_line/ACH-000921?tab=mutations). AlphaFold modeling predicted that wild-type NRIP1 forms a stable complex with FOXO3, whereas the mutant fails to interact stably (Fig. 6A). Simulation of the wild-type NRIP1-FOXO3 complex bound to the TFAM target sequence indicated full exposure of the FOXO3 DNA-binding domain, allowing effective recognition of the TFAM motif (Fig. 6B).

Consistently, Western blot analysis confirmed robust NRIP1 expression in A549 cells but complete absence in H157 cells, while FOXO3 levels were comparable and TFAM expression was significantly lower in H157 cells (*P* < 0.05; Fig. 6C, D). NRIP1 overexpression in A549 cells enhanced TFAM protein without affecting FOXO3, and transfection of wild-type NRIP1 into H157 cells restored NRIP1 expression and markedly upregulated TFAM (*P* < 0.05 vs. Ctrl; Fig. 6E–H). These results suggest that NRIP1 stabilizes FOXO3, facilitating its binding to TFAM and promoting transcriptional regulation.

### NRIP1 acts as a FOXO3 co-activator in radiation resistance

**Fig. 7:**
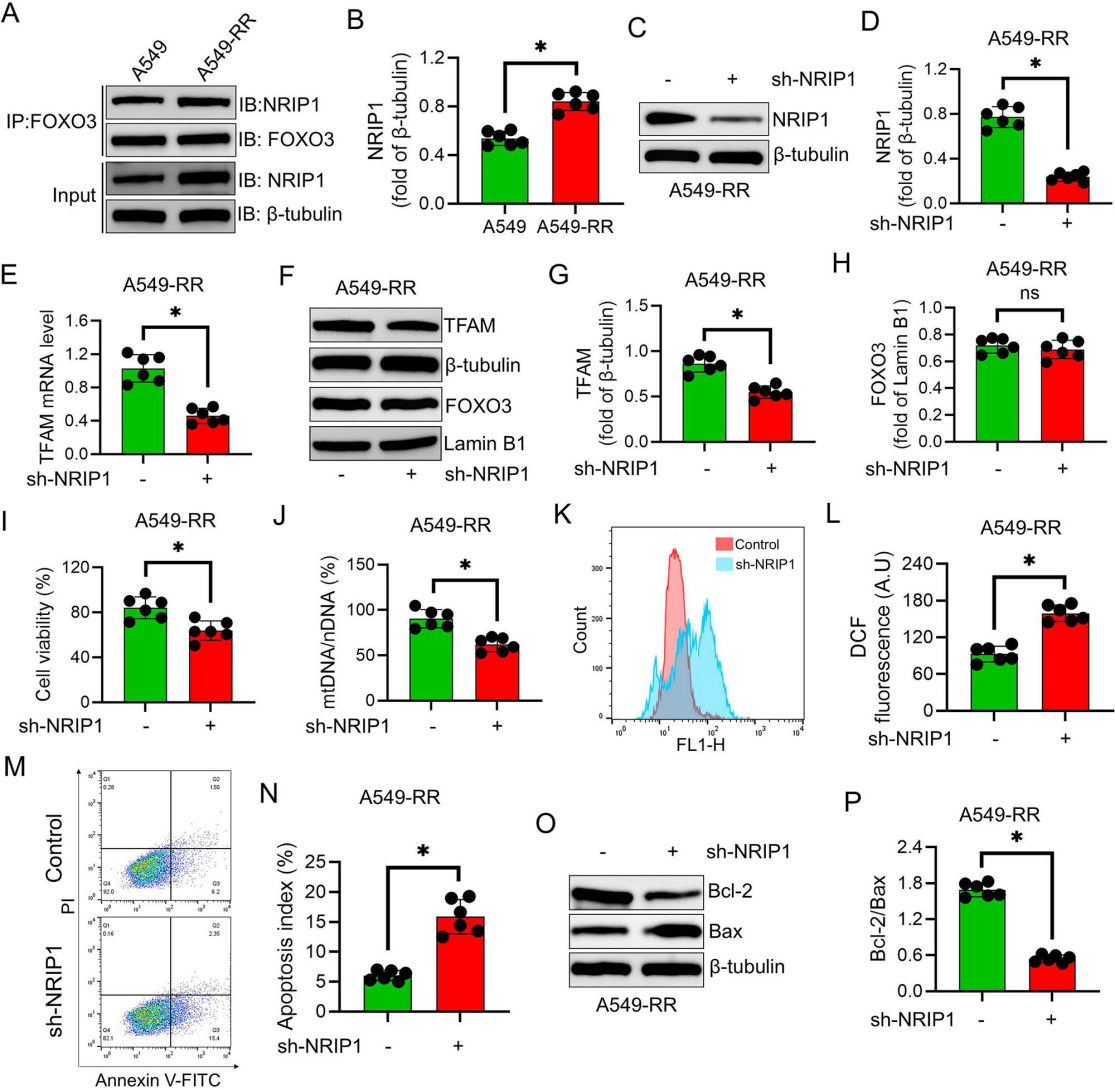
Effects of NRIP1 knockdown on radioresistant NSCLC cells. **A** Co-IP using an anti-FOXO3 antibody followed by Western blotting to detect NRIP1 and FOXO3 interactions in A549 and A549-RR cells. **B** Semi-quantitative analysis of NRIP1 protein levels (normalized to β-tubulin) in A549 and A549-RR cells. **C**,**D** Western blot analysis confirming NRIP1 knockdown efficiency in A549-RR cells. **E** qPCR analysis of TFAM mRNA expression in A549-RR cells following NRIP1 knockdown. **F–H** Western blot analysis of TFAM and nuclear FOXO3 protein levels after NRIP1 knockdown. **I** Cell viability of A549-RR cells measured by CCK-8 assay 24 h after a single 6 Gy radiation dose with or without NRIP1 knockdown. **J** qPCR quantification of mtDNA content in A549-RR cells following NRIP1 knockdown and radiation exposure. **K**,**L** Flow cytometry analysis of intracellular ROS levels in A549-RR cells after NRIP1 knockdown under radiation. **M**,**N** Flow cytometry analysis of apoptosis in A549-RR cells following NRIP1 knockdown after radiation. **O**,**P** Western blot analysis of Bcl-2 and Bax protein levels in A549-RR cells after NRIP1 knockdown and radiation treatment. Data are presented as mean ± SD (*n* = 6). ^*^P < 0.05.

As NRIP1 expression was undetectable in H157 cells, subsequent experiments were conducted using A549 and A549-RR cells. Co-IP demonstrated a strengthened interaction between FOXO3 and NRIP1 in A549-RR cells, coinciding with increased NRIP1 levels (*P* < 0.05; Fig. 7A, B). Functionally, NRIP1 knockdown in A549-RR cells significantly reduced TFAM expression, cell viability, and mtDNA content under irradiation, while markedly increasing ROS accumulation and apoptosis (*P* < 0.05; Fig. 7C–P). Notably, NRIP1 depletion did not alter nuclear FOXO3 levels (Fig. 7F, H), indicating that NRIP1 functions as a FOXO3 co-activator to enhance TFAM transcription without affecting FOXO3 nuclear localization, thereby contributing to radiation resistance.

### FOXO3 knockdown enhances radiosensitivity in vivo

**Fig. 8:**
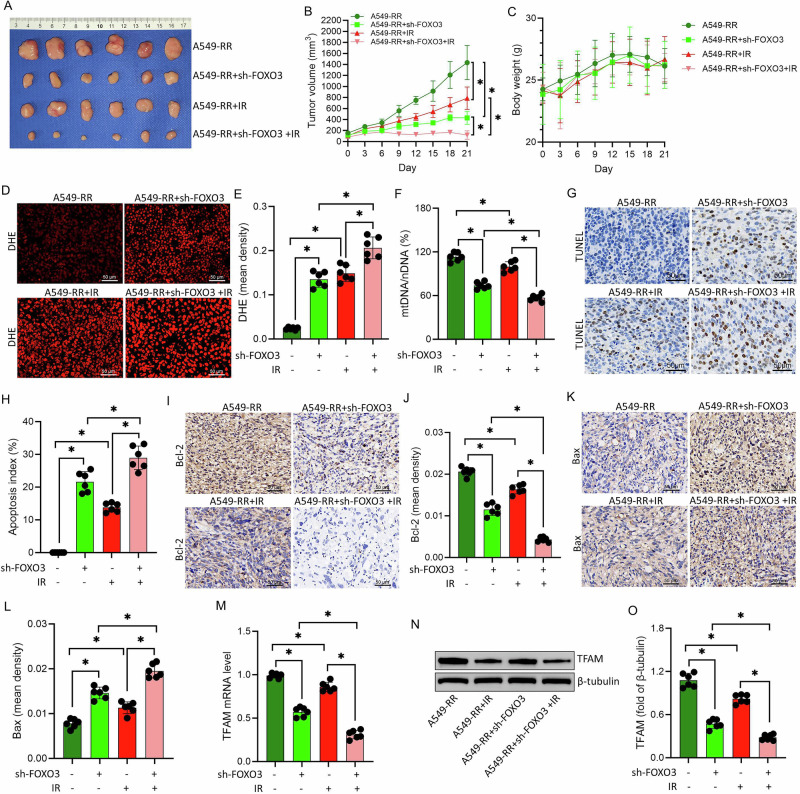
Effect of FOXO3 knockdown on radiation sensitivity in A549-RR xenograft mice. **A** Representative tumor images from each experimental group at the study endpoint. **B** Tumor growth curves showing changes in tumor volume across treatment groups. **C** Body weight monitoring of mice throughout the experimental period. **D** Representative images of DHE staining in tumor tissues from each group to assess oxidative stress. **E** Quantitative analysis of DHE fluorescence intensity shown in (**D**). **F** Comparison of mtDNA content in tumor tissues among different treatment groups. **G** Representative images of TUNEL staining in tumor sections from each group to assess apoptosis. **H** Quantitative analysis of apoptotic cell ratios corresponding to (**G**). **I** Representative immunohistochemical images showing Bcl-2 expression in tumor tissues from each group. **J** Quantitative analysis of Bcl-2 staining intensity in (**I**). **K** Representative immunohistochemical images showing Bax expression in tumor tissues from each group. **L** Quantitative analysis of Bax staining intensity in (**K**). **M** Comparison of TFAM mRNA transcription levels in tumor tissues across the experimental groups. **N**,**O** Western blot analysis of TFAM protein expression in tumor tissues from the respective experimental groups. Data are presented as mean ± SD (*n* = 6). ^*^P < 0.05.

In A549-RR xenograft models, FOXO3 knockdown significantly suppressed tumor growth, an effect further amplified by irradiation (*P* < 0.05; Fig. 8A, B), without affecting overall body weight (Fig. 8C). DHE staining revealed increased ROS levels in FOXO3-silenced tumors after radiation (*P* < 0.05; Fig. 8D, E), accompanied by reduced mtDNA content (Fig. 8F).

TUNEL assays and immunohistochemistry demonstrated enhanced apoptosis in FOXO3-knockdown tumors, with decreased Bcl-2 and increased Bax expression, particularly following irradiation (*P* < 0.05; Fig. 8G-L), indicating increased radiosensitivity in vivo. Furthermore, both TFAM mRNA and protein levels were significantly reduced in FOXO3-silenced tumors, irrespective of radiation treatment (*P* < 0.05; Fig. 8M-O).

## Discussion

TFAM plays a critical role in mitochondrial DNA (mtDNA) replication, transcription, and maintenance. As a nuclear-encoded mitochondrial gene, TFAM has been proposed as a prognostic biomarker in NSCLC patients [13]. Prior work showed that TFAM downregulation impairs NSCLC cell cycle progression, reduces migration, and limits bioenergetics via ROS-dependent pathways [14], highlighting TFAM inhibition as a potential therapeutic strategy [15]. However, its contribution to NSCLC radioresistance remains unexplored. Radiotherapy induces oxidative stress, and under these conditions, TFAM expression has been reported to increase as an adaptive response. Ionizing radiation elevates TFAM levels and mtDNA replication in cancer cells [16]. TFAM knockout enhances radiation-induced cell death, likely through modulation of mitochondrial ROS via p53/TIGAR and ATM/p38/HuR signaling. [16, 17].

In our study, radioresistant A549-RR and H157-RR NSCLC cells showed significantly increased survival after irradiation. This was accompanied by reduced ROS accumulation, enhanced mtDNA replication and mitochondrial mass, decreased apoptosis, and upregulation of TFAM at both mRNA and protein levels. Importantly, TFAM knockdown in these resistant cells reduced survival, increased oxidative stress, decreased mtDNA content and mitochondrial number, and increased apoptosis, supporting a pivotal role for TFAM in maintaining mitochondrial integrity and radioresistance.

FOXO3 is traditionally regarded as a tumor suppressor; however, its biological role appears to be context-dependent. Its function is regulated by post-translational modifications and interactions with other regulatory proteins [18]. Under oxidative stress, FOXO3 can translocate to the nucleus and promote transcription of antioxidant and repair genes [10]. While some studies report that radiation induces FOXO3 nuclear localization and apoptosis [19], in other cancer types (e.g., nasopharyngeal [20], prostate [21], and colorectal [22]), FOXO3 expression decreases during the development of radioresistance; its activation restores radiosensitivity. In contrast, in NSCLC, FOXO3 overexpression promotes DNA repair and enhances resistance to ionizing radiation [23]. Consistently, we found that FOXO3 protein levels and nuclear accumulation were significantly elevated in our radioresistant NSCLC lines, suggesting a survival-promoting rather than apoptotic role.

Mechanistically, FOXO3 recognizes the consensus sequence 5’-[AG]TAAA[TC]A-3’ [24–26], and the TFAM coding region contains multiple such motifs. Guided by AlphaFold modeling and ChIP-qPCR, we confirmed that FOXO3’s DNA-binding domain can stably interact with these sites. FOXO3 knockdown reduced TFAM mRNA and protein, whereas LOM612 treatment, which promotes FOXO3 nuclear translocation, strongly increased TFAM expression. Functionally, FOXO3 silencing compromised viability, increased oxidative stress, impaired mtDNA replication, and triggered apoptosis in irradiated cells, whereas LOM612’s protective effect was abrogated by TFAM co-silencing. These observations point to a model in which FOXO3 directly drives TFAM transcription to bolster mitochondrial function and survival under radiation stress. Our in vivo findings reinforce the mechanistic model, indicating that FOXO3 activity is required to sustain mitochondrial resilience and maintain the radioresistant phenotype in tumor tissue.

Importantly, in addition to the FOXO3-TFAM axis, FOXO3 also enhances protective mitophagy via the PINK1/Parkin pathway, thereby eliminating damaged mitochondria and promoting survival under radiation stress [27]. While mitophagy clears dysfunctional mitochondria, the FOXO3-TFAM axis supports mitochondrial regeneration—together forming a dual strategy to preserve both mitochondrial quality and quantity. Interestingly, other studies report that FOXO3 can repress TFAM and other nuclear-encoded mitochondrial genes by antagonizing c-Myc, leading to reduced mtDNA copy number and diminished respiratory function [9]. Thus, FOXO3 appears to play a context-dependent dual role: under radiotherapy stress, it activates TFAM to support survival, whereas in other cellular states, it may restrain mitochondrial output via c-Myc inhibition.

We further identified NRIP1 as a critical co-regulator in this context. NRIP1 is a well-known transcriptional coregulator that influences many transcription factors in cancer [28–32]. Our data suggest that NRIP1 deficiency reduces FOXO3-driven TFAM expression, whereas re-expression of NRIP1 enhances TFAM transcription without altering FOXO3 abundance. Co-IP confirmed the FOXO3–NRIP1 interaction, which was increased in radioresistant cells, and loss of NRIP1 impaired mitochondrial content, increased oxidative stress, and increased apoptosis in radioresistant NSCLC cells, indicating enhanced radiosensitivity. Thus, NRIP1 likely acts as a coactivator that strengthens FOXO3’s transcriptional regulation of TFAM, contributing to radioresistance.

Nevertheless, our study has several limitations. We conducted most experiments in established cell lines and in T-cell–deficient xenograft models, which do not fully recapitulate the immune microenvironment or patient heterogeneity. Moreover, we have not yet validated our findings in clinical NSCLC specimens, nor explored the efficacy of pharmacologic targeting of the NRIP1–FOXO3–TFAM axis. Future studies should employ immunocompetent models, patient-derived xenografts, and clinical samples, as well as develop small-molecule interventions to modulate FOXO3 nuclear translocation or NRIP1 coactivation.

From a clinical perspective, the NRIP1–FOXO3–TFAM signaling axis offers promising therapeutic opportunities. Disruption of NRIP1–FOXO3 interactions or inhibition of FOXO3 nuclear localization may reduce TFAM-mediated mitochondrial protection, thereby enhancing radiotherapy effectiveness. In addition, nuclear FOXO3, NRIP1, or TFAM expression could serve as biomarkers to stratify NSCLC patients according to their likelihood of radioresistance and guide personalized radiotherapy strategies.

In summary, our findings reveal a novel regulatory mechanism in which FOXO3 nuclear accumulation directly upregulates TFAM transcription, supported by NRIP1 coactivation, to maintain mitochondrial integrity and promote radioresistance in NSCLC. Together with mitophagy-mediated mechanisms, this dual regulation of mitochondrial homeostasis represents a cancer cell adaptive mechanism to survive irradiation, and targeting this axis may provide a new route to sensitize tumors to radiotherapy.

## Materials and Methods

### Establishment of radioresistant cell lines

Human lung adenocarcinoma A549 cells and lung squamous carcinoma NCI-H157 (H157) cells were obtained from FuHeng Biology (Shanghai, China). Cell line authentication was confirmed by short tandem repeat (STR) profiling, and mycoplasma contamination was routinely tested every 4–6 weeks. To generate radioresistant sublines, parental A549 and H157 cells in exponential growth phase were subjected to fractionated X‑ray irradiation using an X‑RAD320iX generator (Precision X‑Ray, USA) at a dose rate of ~6.4 Gy/min. Cells were irradiated with 2 Gy per fraction, followed by immediate medium replacement and a 24 h recovery period prior to the next dose. This cycle was repeated 30 times, yielding a cumulative dose of ~60 Gy, based on previously published relevant protocols [33]. After completing the final fraction, surviving cells were cultured without further irradiation for at least 4 weeks to allow phenotypic stabilization and the recovery of proliferative capacity. The resulting sublines were designated A549‑RR and H157‑RR, and cryopreserved in aliquots for downstream use.

### Gene knockdown vector construction

Specific small interfering RNA (siRNA) sequences targeting human *TFAM* and *NRIP1* (Table 1) were designed using the BLOCK-iT™ RNAi Designer (Invitrogen, USA). The sequences were synthesized and cloned into the pSilencer™ 2.1-U6 neo vector (Ambion, USA). Target siRNA sequences were first amplified by PCR and digested with EcoRI to enable directional ligation. The ligation products were transformed into competent *E. coli*, and positive clones were confirmed by restriction enzyme digestion and sequencing.

**Table 1:** Gene Targeting Sequences for siRNA and shRNA Constructs.

Gene name	Species	Gene ID	Target Sequence (5’ to 3’)	Region
*TFAM*	Human	7019	GTAAGTTCTTACCTTCGATTT	CDS
*FOXO3*	Human	2309	CATGTTCAATGGGAGCTTGGA	CDS
*NRIP1*	Human	8204	GCGGAGAAGAATGAGTATGAA	CDS

For FOXO3 knockdown, specific short hairpin RNA (shRNA) sequences were synthesized (Table 1) and cloned into the pLKO.1 vector (Addgene, USA) using AgeI and EcoRI restriction sites. Positive clones were verified by digestion and sequencing. To achieve efficient gene delivery, the pLKO.1-shFOXO3 construct was packaged into AAV2/9 viral particles by co-transfection with AAV packaging plasmids (pAAV-RC and pHelper) into HEK293T cells. Viral particles were harvested after 48 hours and purified using the AAVpro Purification Kit (Takara, Japan). Knockdown efficiency was confirmed by quantitative PCR and Western blotting.

For transient knockdown experiments, cells were transfected with the siRNA constructs using Lipofectamine™ 2000 (Invitrogen, USA) according to the manufacturer’s protocol. TFAM or NRIP1 expression levels were assessed 48 h post-transfection by Western blotting or quantitative reverse transcription PCR (qRT-PCR) to confirm knockdown efficiency. All plasmid constructs were verified by restriction digestion and sequencing to ensure correct insertion of the target sequences.

### NRIP1 overexpression construct and transfection

A human NRIP1 overexpression plasmid was generated using the OriGene RG202101 clone (Rockville, MD, USA), which encodes full‑length NM_003489 NRIP1 fused to tGFP in the pCMV6‑AC‑GFP vector. The plasmid was reconstituted in sterile water, transformed into *E. coli*, and amplified under ampicillin selection. Purified plasmid DNA was transfected into cells using Lipofectamine. Expression of NRIP1‑tGFP was confirmed by Western blot with anti‑GFP or anti‑NRIP1 antibodies, and by fluorescence microscopy to verify intracellular localization. Functional rescue was assessed by comparing downstream NRIP1 target gene expression via qPCR between NRIP1‑overexpressing cells and cells transfected with the empty vector control.

### CCK-8 assay for cell viability

Cells were seeded at 1000 cells per well in 96-well plates. When cultures reached approximately 90% confluence, they were irradiated with X-rays at doses of 0, 2, 4, 6, and 8 Gy. Immediately after irradiation, the medium was replaced. Twenty-four hours later, 10 μL of CCK-8 reagent was added to each well, and cells were incubated at 37 °C for 1 h. Absorbance was measured at 450 nm using a microplate reader, with optical density (OD) values serving as an indicator of cell viability.

### Flow cytometry analysis

Cells were seeded at a density of 1 × 10⁵ per well in 48-well plates and allowed to attach for 24 h. For reactive oxygen species (ROS) measurement, cells were treated according to the experimental design and incubated with 2′,7′-dichlorodihydrofluorescein diacetate (H₂DCFDA) at 37 °C in the dark for 30 min. To stop the oxidation reaction, Trolox solution was then added, followed by an additional 10 min incubation under the same conditions. ROS levels were determined by flow cytometry (BD FACSCalibur, BD Biosciences, USA) using the FITC channel, with fluorescence intensity reflecting intracellular ROS.

For apoptosis assessment, cells underwent the designated treatments and were stained with Annexin V-FITC and propidium iodide (PI) following the manufacturer’s instructions. After 15 min of incubation at room temperature in the dark, samples were immediately analyzed by flow cytometry. Apoptotic cell populations were quantified based on Annexin V and PI staining using FlowJo software (FlowJo LLC, USA).

### Detection of Oxidative Stress

Cells were collected and lysed by mechanical disruption. Following centrifugation to remove debris, the supernatant was used to measure Glutathione Peroxidase (GSH-PX) activity and Malondialdehyde (MDA) levels. Assays were performed using commercial kits (Jiancheng Bioengineering Institute, Nanjing, China) according to the manufacturer’s instructions.

### Quantitative PCR (qPCR) Analysis

The mRNA expression of TFAM and mitochondrial DNA (mtDNA) content were quantified by qPCR. Total RNA was extracted using TRIzol reagent, and concentration and purity were determined spectrophotometrically. Complementary DNA (cDNA) was synthesized from RNA using a reverse transcription kit according to the manufacturer’s instructions. For mtDNA analysis, total DNA was extracted using a commercial DNA extraction kit.

qPCR amplification was performed using SYBR Green PCR Master Mix (ThermoFisher, Waltham, MA, USA) with specific primers for TFAM (mRNA) and COX I (mtDNA) (Table 2). β-Tubulin served as the internal reference for mRNA normalization, and β-actin was used for mtDNA normalization. Cycling conditions included initial denaturation at 95 °C for 5 min, followed by 40 cycles of 95 °C for 10 ss, 60 °C for 30 ss, and 72 °C for 30 s.

**Table 2:** Sequences of Primers Used for qPCR.

Gene name	Species	Primer sequence (5’- > 3’)
*TFAM*	Human	F: CTTATAGGGCGGAGTGGCAG R: TGGCAGAAGTCCATGAGCTG
*TUBB*	Human	F: CTCTCAGAACCTTCCTGCCG R: CCAGAACTTGGCACCGATCT
*COX1*	Human	F: CCACAGTTTCATGCCCATCG R: AGGCGACAGCGATTTCTAGG
*ACTB*	Human	F: CTCGCCTTTGCCGATCC R: TCTCCATGTCGTCCCAGTTG

Relative *TFAM* mRNA and mtDNA levels were calculated using the 2^−ΔΔCt^ method, with mtDNA content expressed as the ratio of mtDNA to nuclear DNA.

### Chromatin Immunoprecipitation and qPCR (ChIP-qPCR)

ChIP-qPCR was performed to assess the binding of FOXO3 to predicted motifs (5′-[A/G]TAAA[T/C]A) within the TFAM coding region, with *Cdkn1b* (*p27*) as a positive control [34]. Radiation-resistant and control cells were crosslinked with 1% formaldehyde and quenched, then lysed. Chromatin was sonicated to fragments of 200–600 bp. Immunoprecipitation was carried out using a FOXO3-specific antibody (ThermoFisher, Waltham, MA, USA), with IgG as a negative control. After washing, crosslinks were reversed at 65 °C overnight, and DNA was purified following proteinase K digestion. qPCR primers (Table 3) targeted TFAM motifs 1–3; for motifs 2 and 3, adjacent regions were amplified due to primer design limitations. A distal TFAM coding region served as a negative control. Standard curves were generated from serial dilutions of input DNA, and ChIP-qPCR signals were normalized as percentage of input or relative fold enrichment.

**Table 3:** Sequences of Primers Used for ChIP‑qPCR.

Gene name	Sequence ID	Product length	Forward primer (5’- > 3’)	Reverse primer (5’- > 3’)
*TFAM* (Motif 1)	NM_003201.3	101	TGAGCTCAGAGAGCACATAGGA (Template: 2973 → 2994)	TCTATGCTGCATTTGTCCCGA (Template: 3073 → 3053)
*TFAM* (Motif 2)	NM_003201.3	88	AGGGAGGAAAGGTGTAAACAG(Template: 3232 → 3252)	AACAGAGAACCAAATACAAGCTGAT (Template: 3319 → 3295)
*TFAM* (Motif 3)	NM_003201.3	154	TGTCAGTCTCTCTCATTGTTCAC (Template: 4828 → 4850)	AGTCAACTTTATTTACTGGCTCCT(Template: 4981 → 4958)
*TFAM* (Neg)	NM_003201.3	690	TGGAAAACCAAAAAGACCTCGT (Template: 594 → 615)	TATCACAGAACACCGTGGCT (Template: 1283 → 1264)
*Cdkn1b* (*p27*)			TTTTTACGCATCGCTGCTACT	CGTAGTGAGGTCGGCTAAGC

### Experimental animals and treatment

Female 4-week-old BALB/c nude mice were obtained from GemPharmatech (Nanjing, China) and maintained under controlled temperature and humidity with a 12 h light/dark cycle, with free access to food and water. All procedures complied with the Guide for the Care and Use of Laboratory Animals (National Research Council, 8th edition, 2011) and were approved by the Institutional Animal Care and Use Committee of The First Affiliated Hospital of Nanchang University (No. CDYFY-IACUC202501GR050).

After a 1-week acclimatization, 1 × 10⁶ cancer cells were subcutaneously implanted into the right forelimb shoulder to establish tumor xenografts. When tumors reached ~100 mm³, mice were randomly assigned to four groups: A549-RR, A549-RR+sh-FOXO3, A549-RR + IR, and A549-RR+sh-FOXO3 + IR. The A549-RR group received A549-RR cells only, while the A549-RR+sh-FOXO3 group received cells with stable FOXO3 knockdown via AAV transduction. Mice in the A549-RR + IR group received A549-RR cells followed by radiotherapy using the X-RAD SmART small animal precision radiotherapy system (5 Gy twice weekly for 3 weeks, total 30 Gy), under isoflurane anesthesia. The A549-RR+sh-FOXO3 + IR group underwent the same irradiation schedule after receiving FOXO3-knockdown cells. Sample size (*n* = 6 per group) was determined based on published literature, balancing expected effect size, feasibility, and ethical reduction of animal numbers. As this is an exploratory study, no formal power calculation was performed. Tumor volumes and body weights were recorded every 3 days. Blinding (masking): The investigators responsible for measuring tumor volume, monitoring body weight, and performing downstream analyses (e.g., histological, molecular assays) were blinded to group allocation. Group identity was coded and remained concealed until all data were collected and primary data analysis was completed. On day 21, mice were euthanized by cervical dislocation, and tumors were harvested for downstream analyses.

### Dihydroethidium (DHE) staining

Tumor tissues were frozen, sectioned, and incubated in 10 μM DHE solution at 37 °C in the dark for 30 min. After incubation, sections were washed three times with PBS to remove unbound dye. A small volume of PBS was added, and red fluorescence was observed under a fluorescence microscope (excitation 488 nm, emission 610 nm). Fluorescence intensity, reflecting reactive oxygen species (ROS) levels, was quantified using Image Pro Plus 6.0 software (Minneapolis, MN, USA).

### TUNEL staining

Paraffin-embedded tumor sections were deparaffinized, rehydrated, and treated with Proteinase K at 37 °C for 20 min. Sections were then washed three times with PBS (5 min each) to stop enzyme activity. TUNEL staining was performed using a commercial kit (Servicebio, Wuhan, China) following the manufacturer’s protocol. Sections were incubated with TdT reaction solution at 37 °C for 1 h, followed by DAB chromogen staining. Apoptotic cells were identified by brown-stained nuclei under an optical microscope, and the proportion of TUNEL-positive cells was quantified.

### Immunohistochemical analysis

Paraffin-embedded tumor sections were deparaffinized, rehydrated, and treated with 0.3% hydrogen peroxide in methanol for 30 min to block endogenous peroxidase activity. Sections were then blocked with serum and incubated overnight at 4 °C with primary antibodies against Bcl-2 and Bax. After washing, sections were incubated with the appropriate secondary antibody at room temperature for 1 h. Staining was performed using DAB chromogen, and nuclei were counterstained with hematoxylin. Sections were observed under an optical microscope, photographed, and protein expression was quantified using Image Pro Plus 6.0 software.

### Co-immunoprecipitation (Co-IP)

Cells were collected and lysed in buffer containing protease inhibitors, followed by disruption via sonication or repeated pipetting. Lysates were centrifuged at 14,000 × g for 10 min at 4 °C, and the supernatant was collected as whole-cell lysate. Lysates were incubated overnight at 4 °C with a primary antibody against FOXO3 (Abcam, Cambridge, UK). Protein A/G agarose beads (Beyotime Biotechnology, Shanghai, China) were then added and incubated for 2 h to capture the antibody–antigen complexes. The precipitates were washed multiple times with PBS to remove non-specific proteins, then eluted with SDS-containing buffer and boiled for 5 min. Eluted proteins were separated by SDS-PAGE and probed with primary antibodies against NRIP1 and FOXO3, followed by HRP-conjugated secondary antibodies. Bands were visualized using chemiluminescence, and signal intensities were quantified for analysis.

### Western blotting

Total protein was extracted from cells or tumor tissues, and nucleoproteins and cytoplasmic proteins were separated using a commercial extraction kit. Proteins were resolved by 10% SDS-PAGE and transferred onto PVDF membranes. Membranes were blocked at room temperature for 1 hour and then incubated overnight at 4 °C with primary antibodies against TFAM, FOXO3, NRIP1, Bcl-2, Bax, Lamin B1, and β-tubulin (Abcam, Cambridge, UK). After washing, membranes were incubated with HRP-conjugated secondary antibodies for 1 h, followed by additional washes. Protein bands were visualized using chemiluminescence (ECL), and signal intensities were quantified using ImageJ v2.1.4.7 software (National Institutes of Health, Bethesda, MD, USA).

### Data Analysis

Data are presented as mean ± standard deviation (SD). Statistical analyses were performed using SPSS version 20.0 (IBM Corp., Armonk, NY, USA). Comparisons between two groups were conducted using the independent samples t-test, with Levene’s test assessing variance homogeneity. For comparisons among more than two groups, one-way ANOVA was applied, followed by Tukey’s or Tamhane’s T2 post-hoc tests as appropriate. Two-way ANOVA was used to evaluate the effects of two independent variables and their interaction. Normality and variance homogeneity were verified prior to ANOVA. A *p*-value < 0.05 was considered statistically significant.

## Supplementary information

Full and uncropped western blots

## Data Availability

The datasets used and/or analyzed during the current study are available from the corresponding author on reasonable request.
