# Supplementary material for: NRIP1 co-activates nuclear translocated FOXO3 to upregulate TFAM expression and promote radioresistance in non-small cell lung cancer
Source: Cell Death Discov. 2026 Mar 27;12:196. doi: 10.1038/s41420-026-03028-8 (PMC13144454; doi:10.1038/s41420-026-03028-8)
Supplement: Supplementary file 1 — Full and uncropped western blots [file 41420_2026_3028_MOESM1_ESM.pdf]

Figure 1O

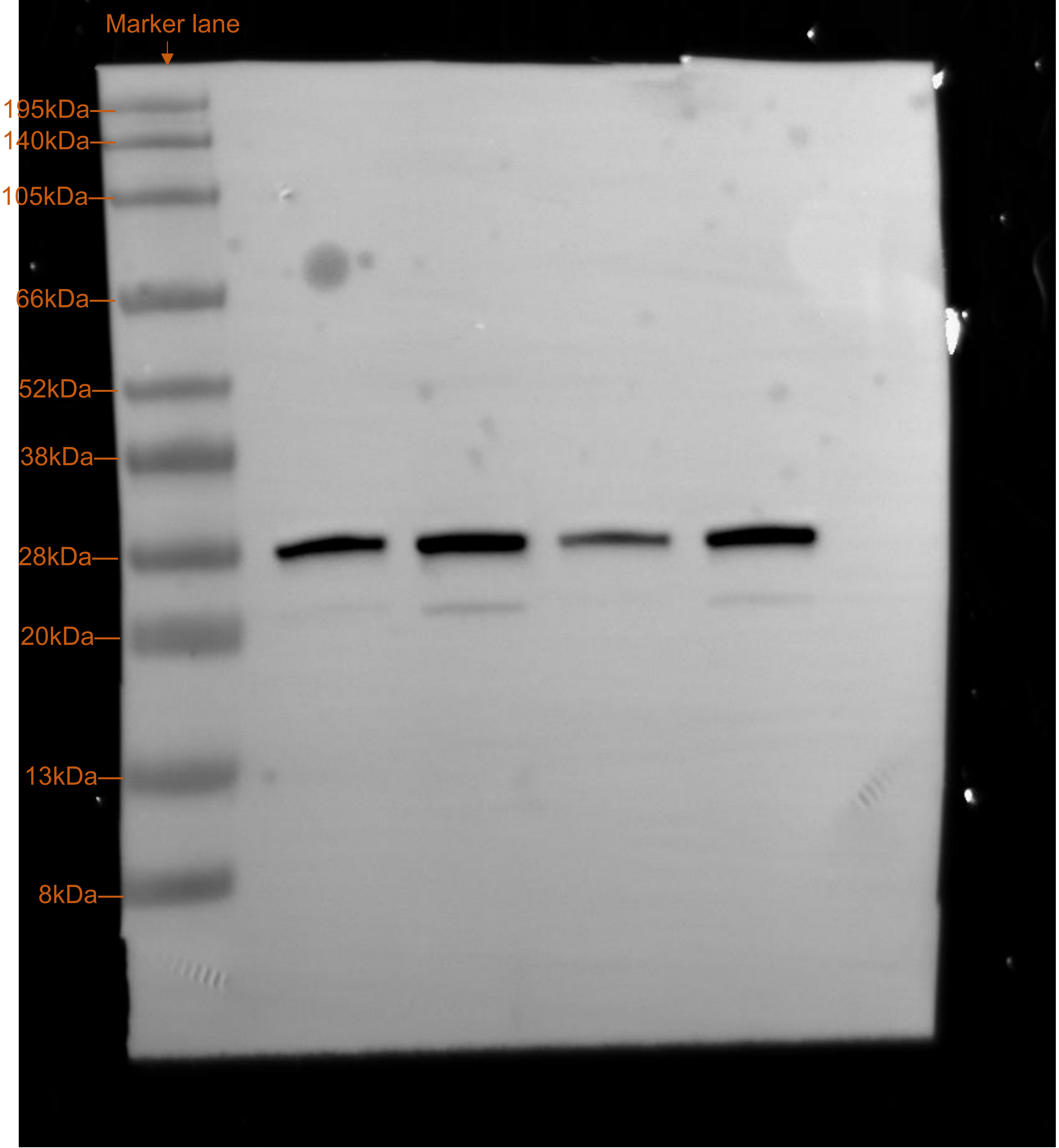

TFAM 29kD

Figure 1O

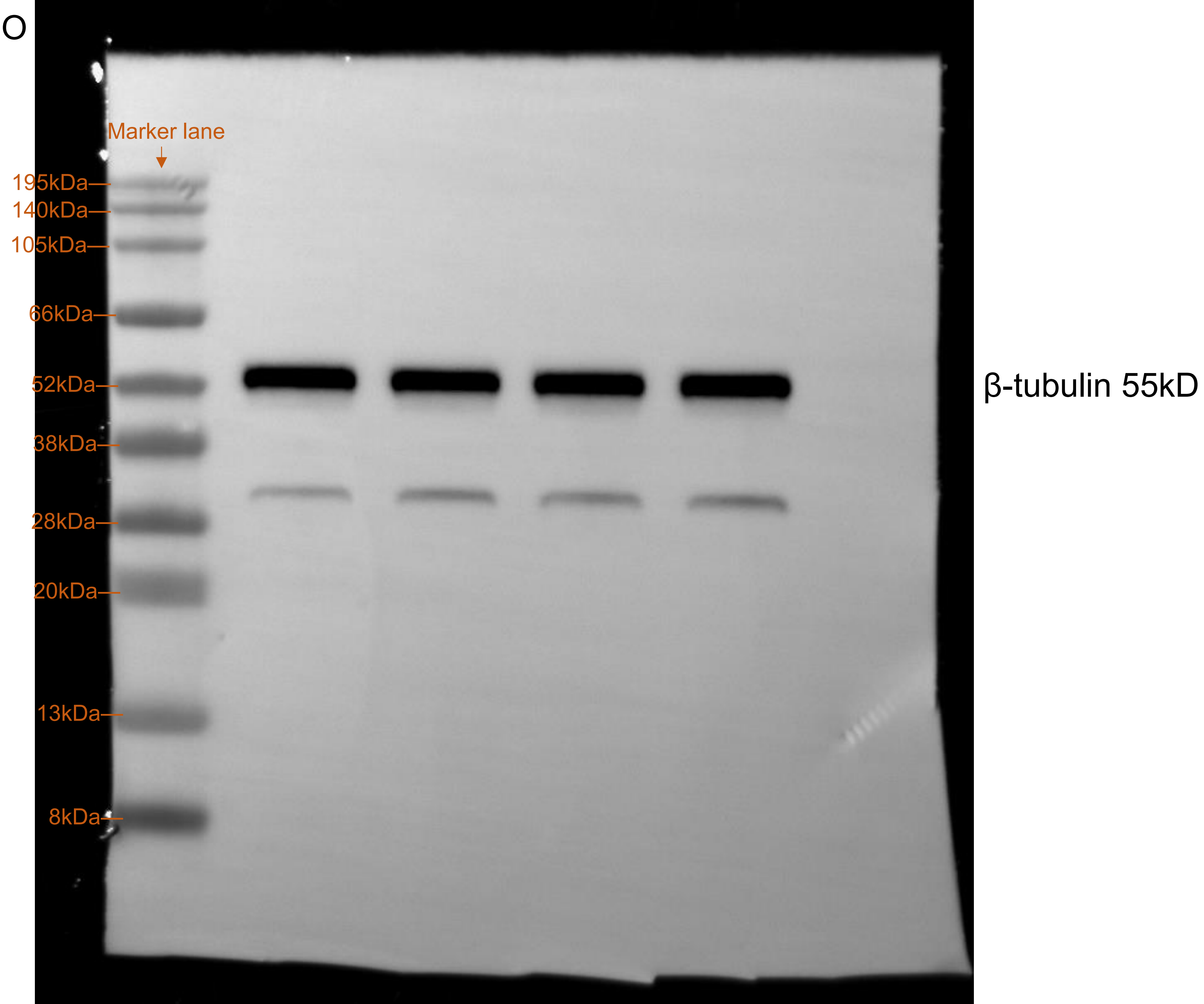

Marker lane
195kDa
140kDa
105kDa
66kDa
52kDa
38kDa
28kDa
20kDa
13kDa
8kDa
β-tubulin 55kD

Figure 2P

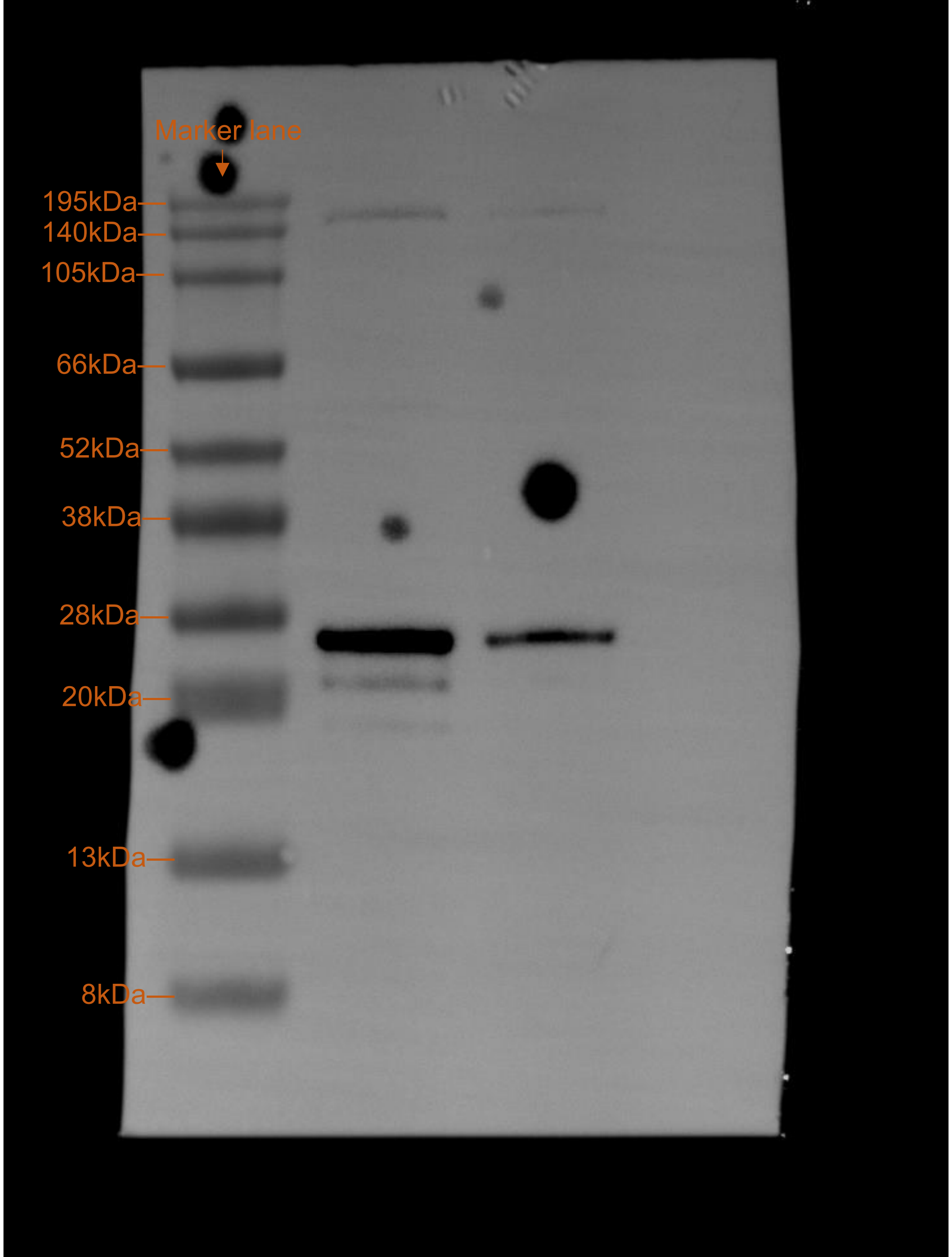

Bcl-2 26kD

Figure 2P

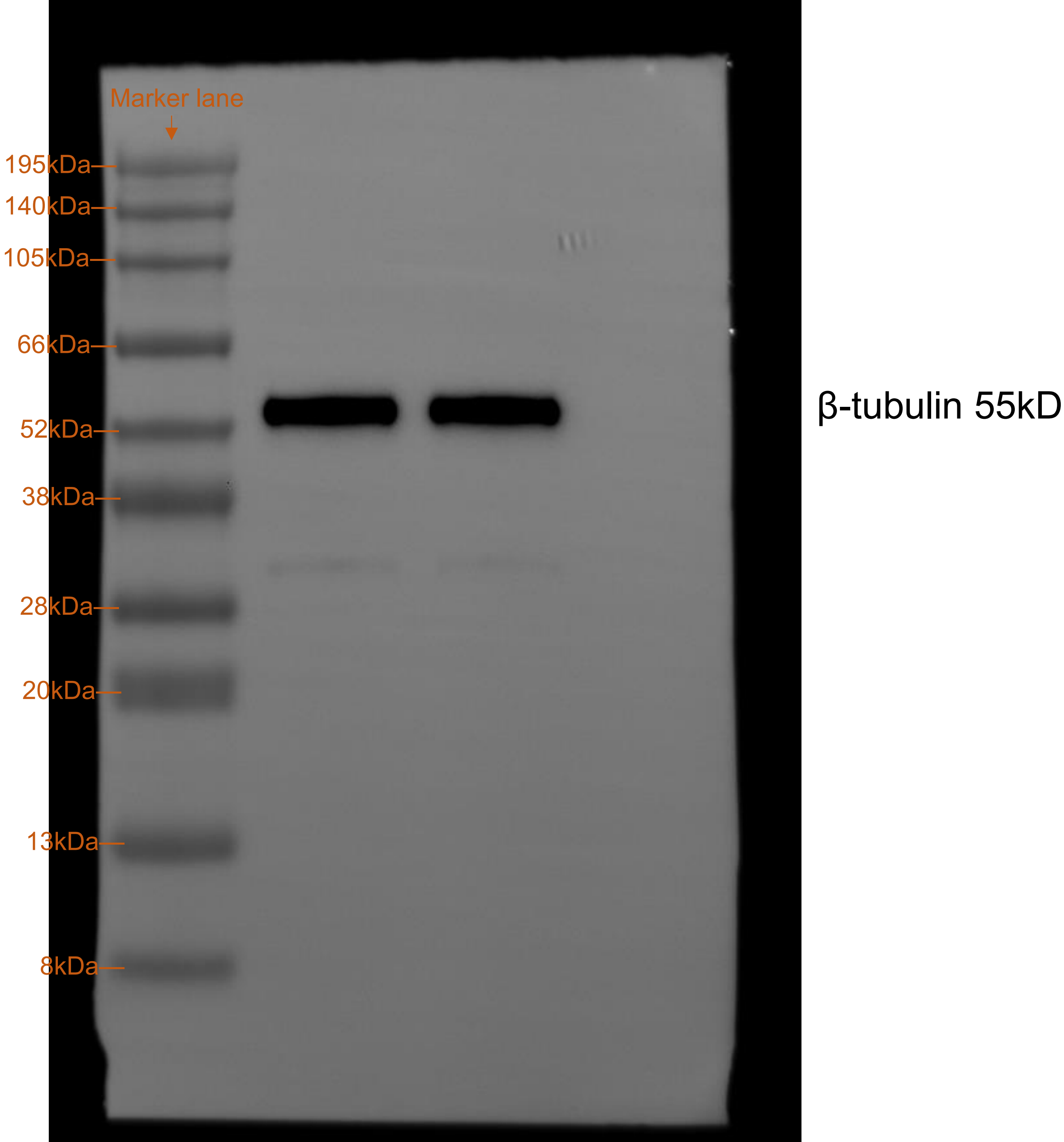

Marker lane
195kDa
140kDa
105kDa
66kDa
52kDa
38kDa
28kDa
20kDa
13kDa
8kDa
β-tubulin 55kD

Figure 2P

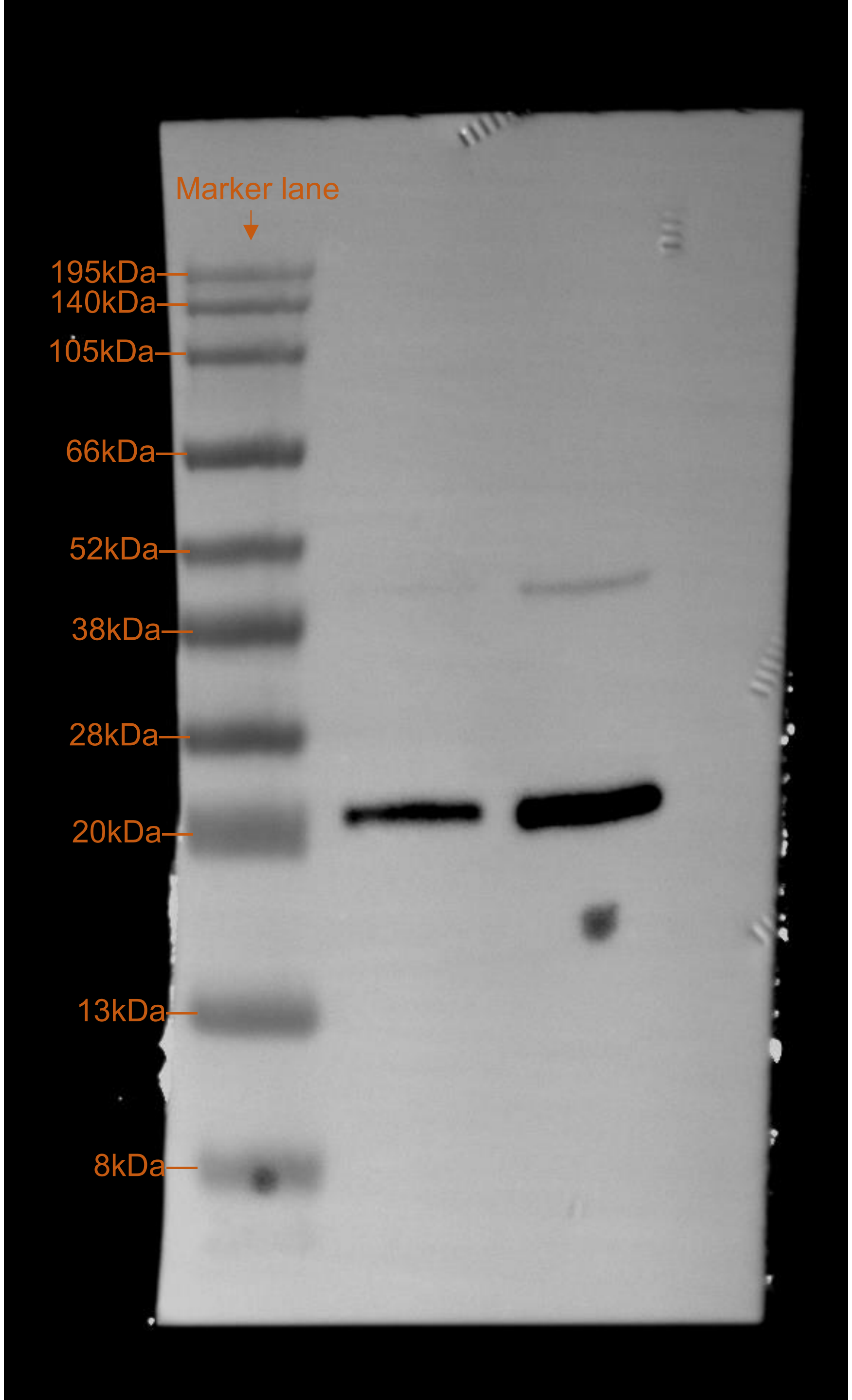

Bax 21kD

Figure 2P

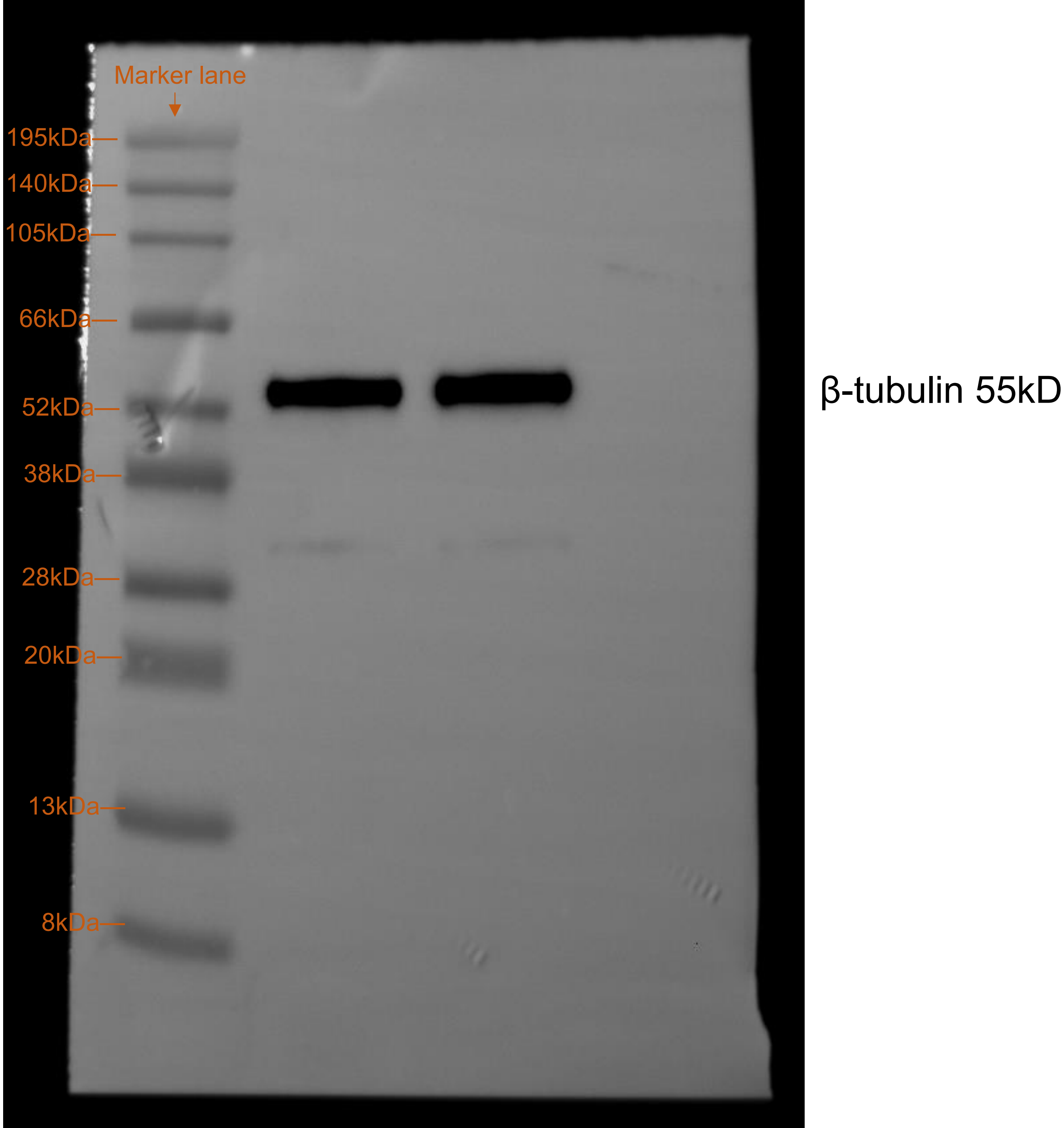

Marker lane
195kDa
140kDa
105kDa
66kDa
52kDa
38kDa
28kDa
20kDa
13kDa
8kDa
β-tubulin 55kD

Figure 2R

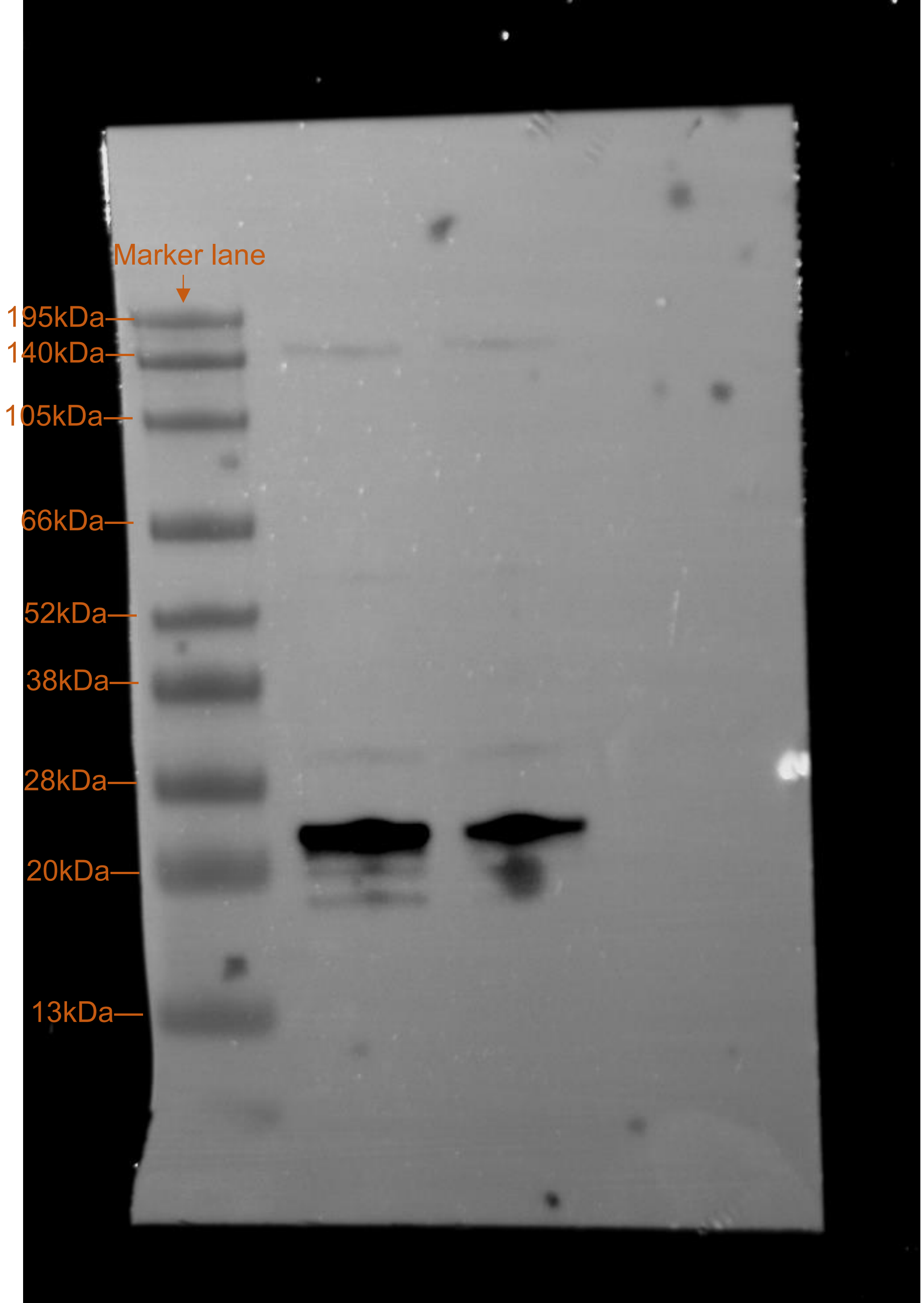

Bcl-2 26kD

Figure 2R

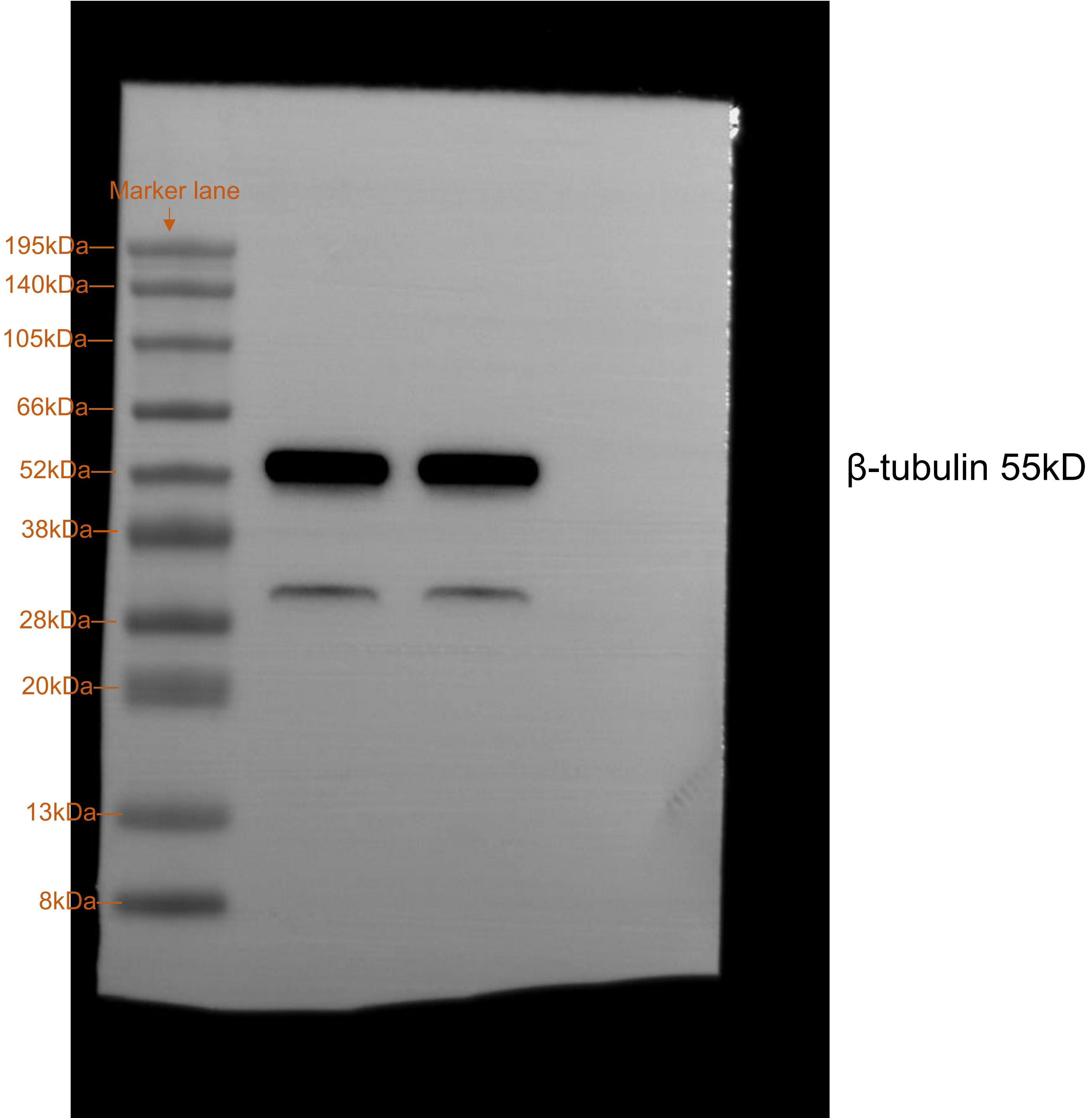

Marker lane
195kDa
140kDa
105kDa
66kDa
52kDa
38kDa
28kDa
20kDa
13kDa
8kDa
β-tubulin 55kD

Figure 2R

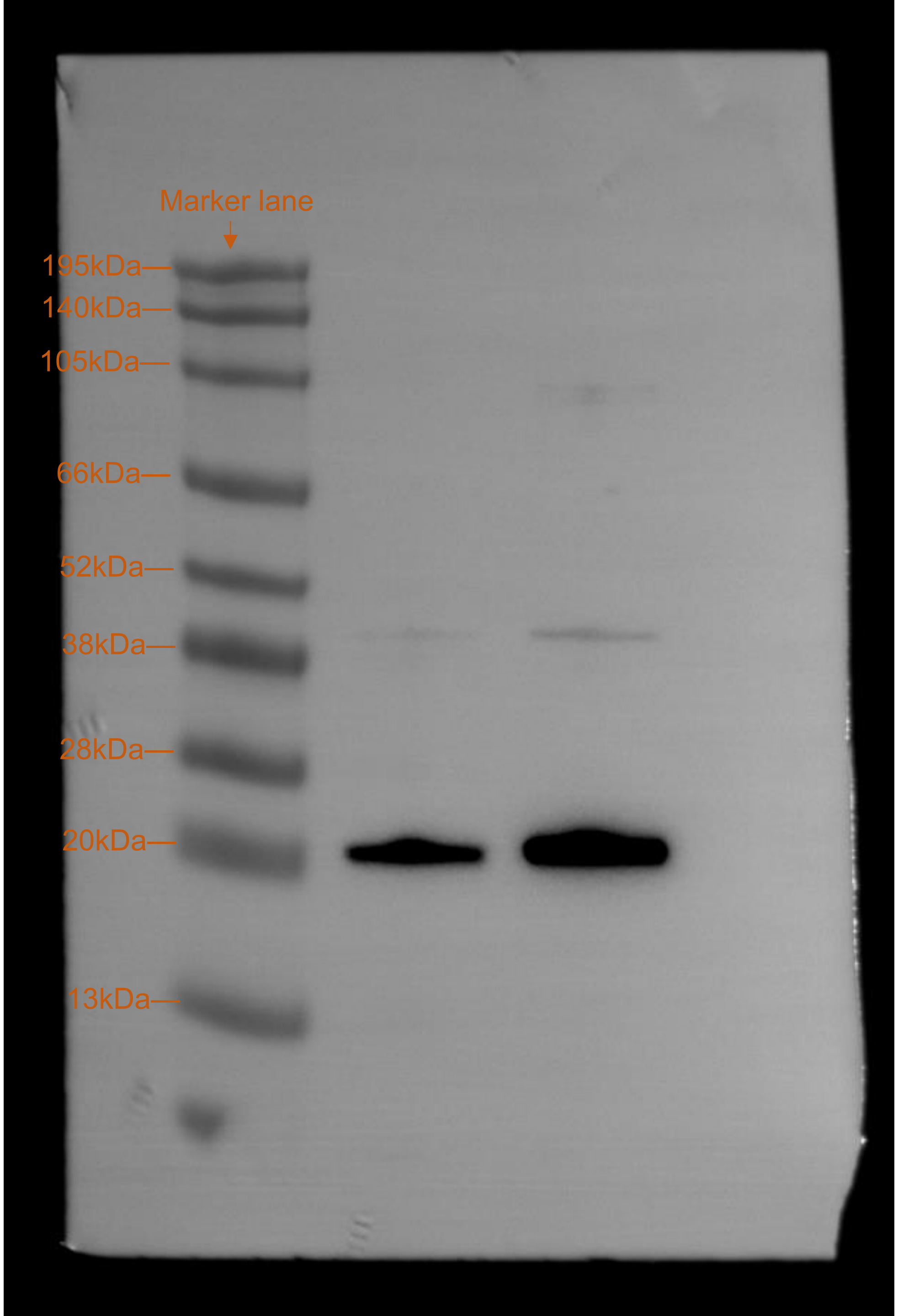

Bax 21kD

Figure 2R

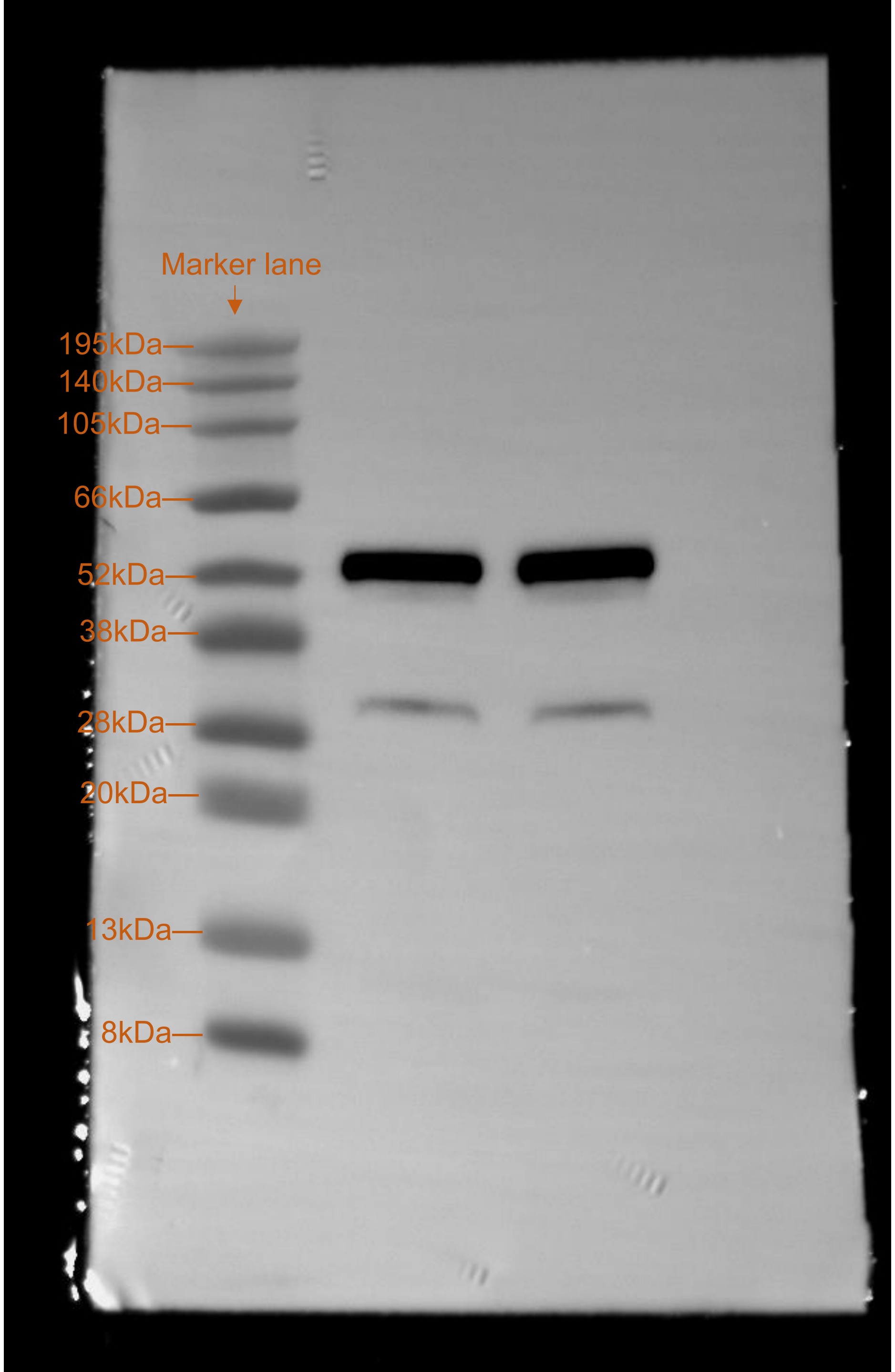

β-tubulin 55kD

Figure 3E

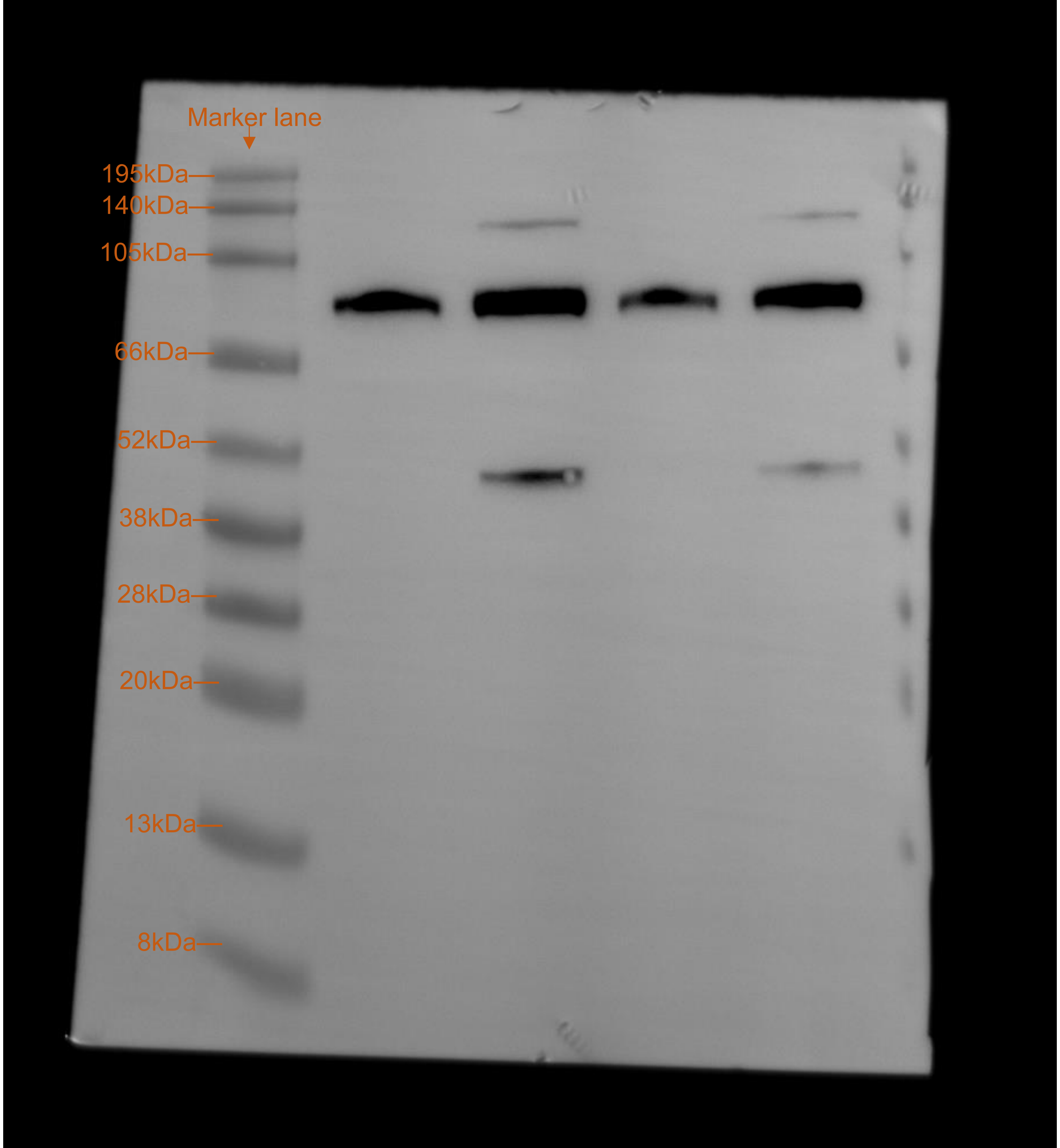

FOXO3 97kD

Figure 3E

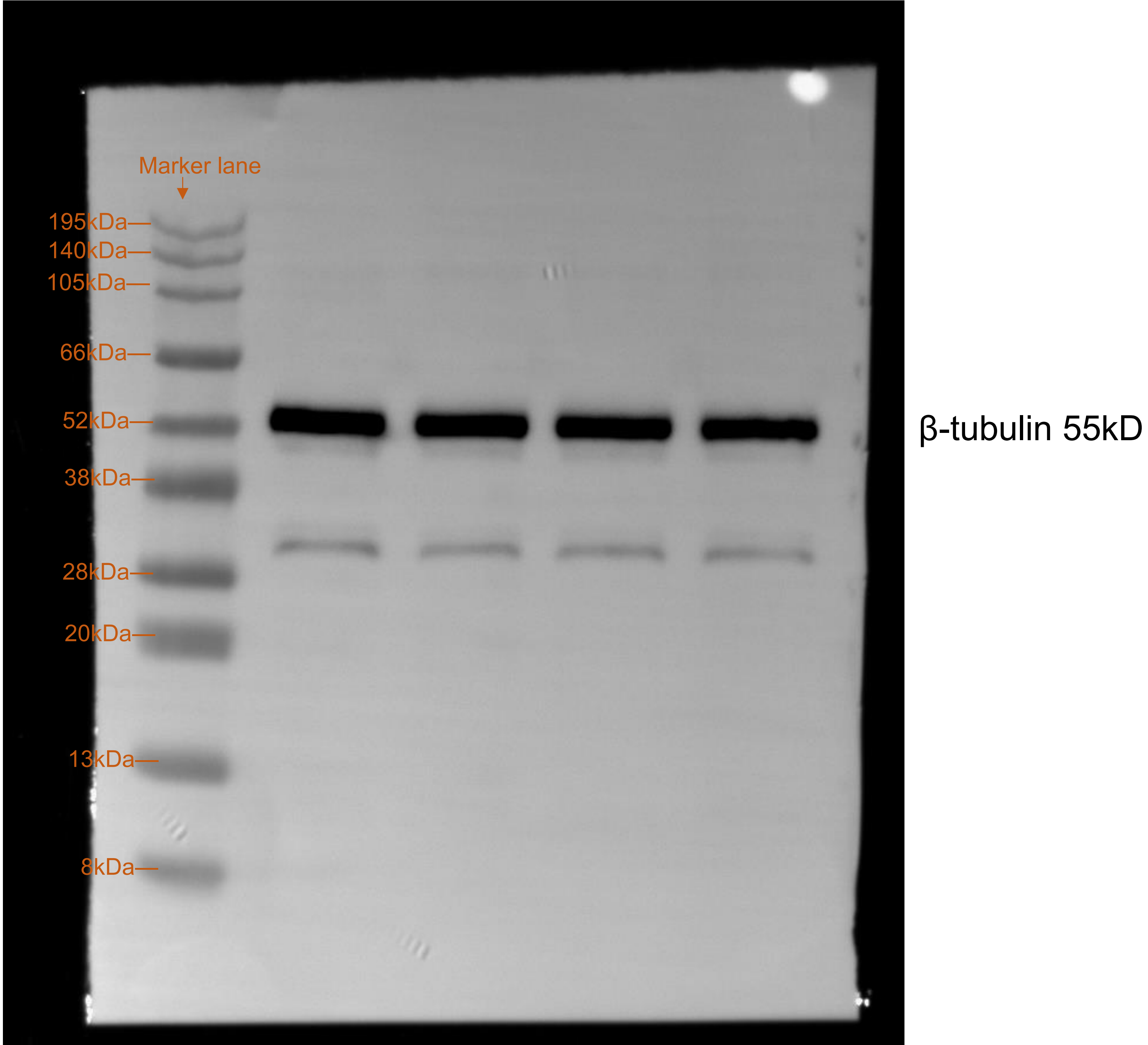

Marker lane
195kDa
140kDa
105kDa
66kDa
52kDa
38kDa
28kDa
20kDa
13kDa
8kDa
β-tubulin 55kD

Figure 3E

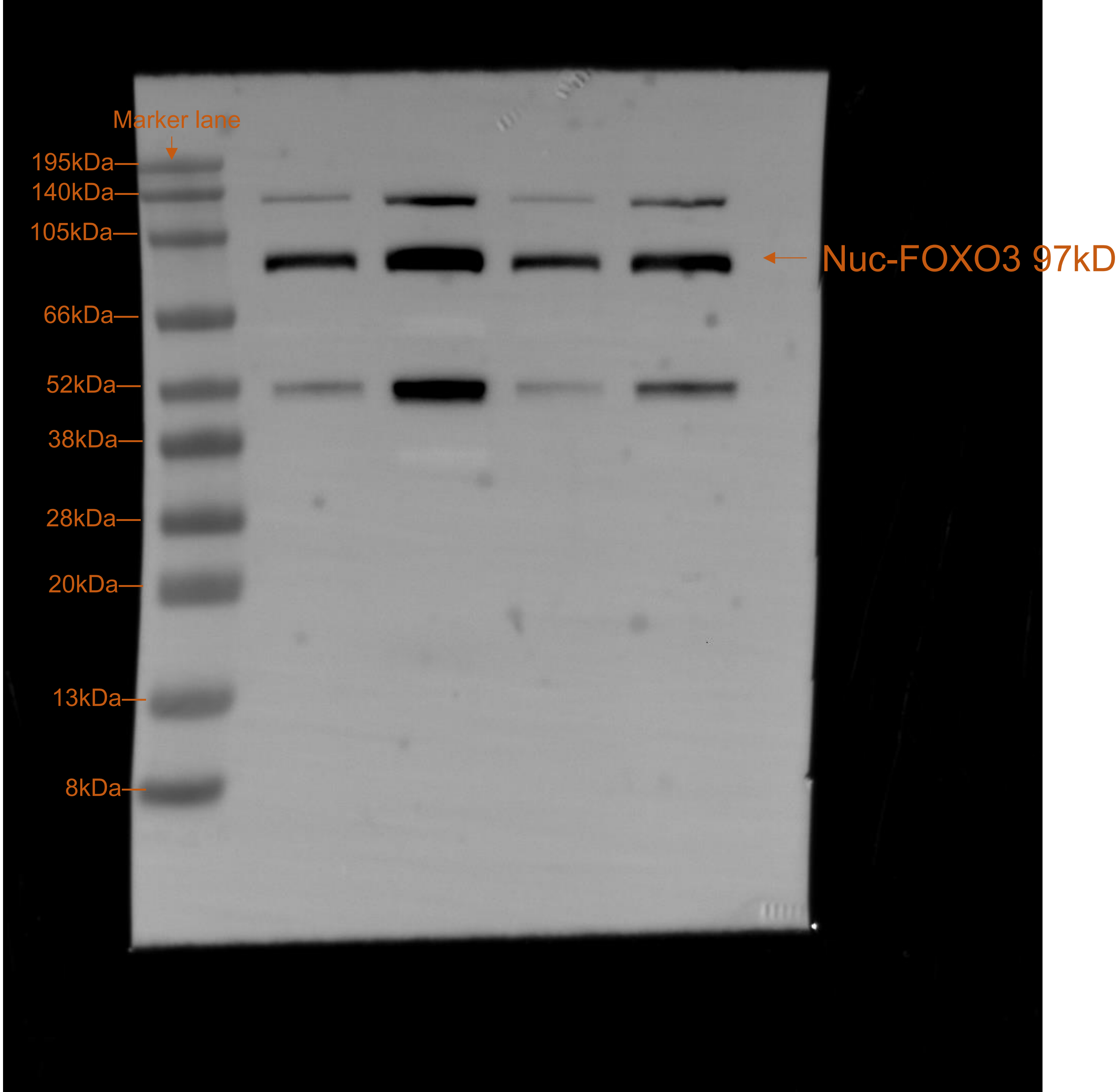
Marker lane
195kDa
140kDa
105kDa
66kDa
52kDa
38kDa
28kDa
20kDa
13kDa
8kDa
Nuc-FOXO3 97kD

Figure 3E

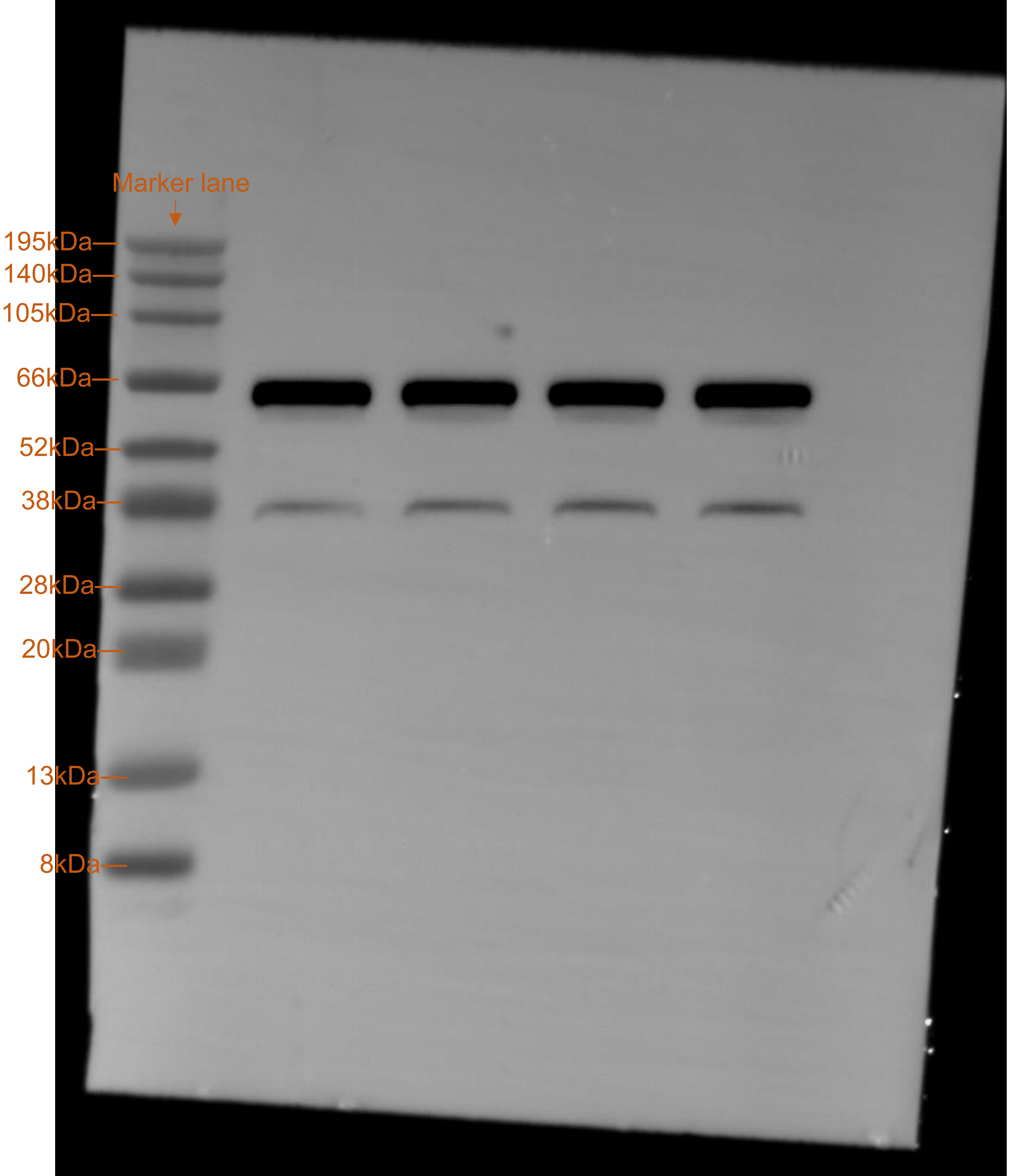

Lamin B1 66kD

Figure 3E

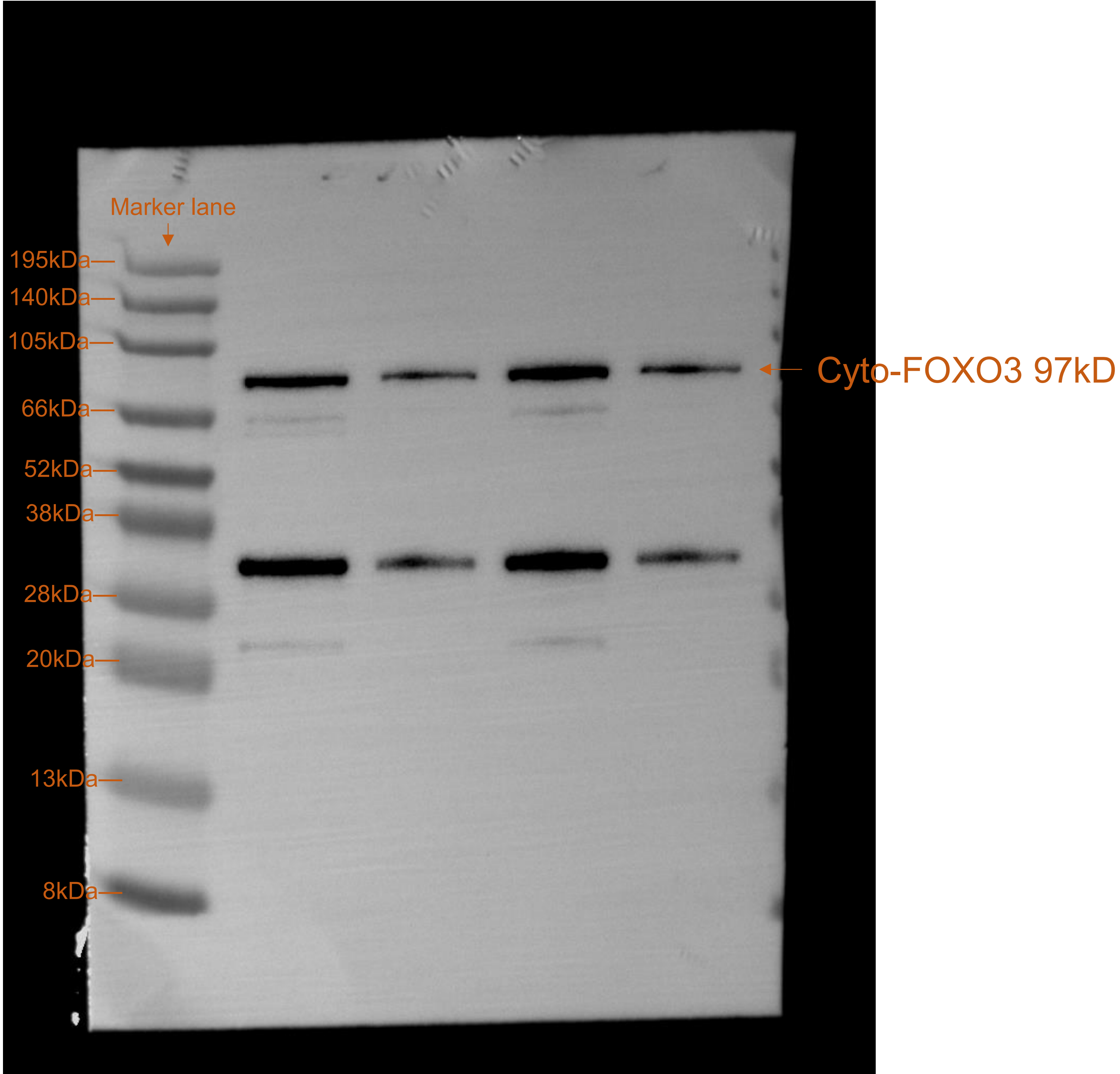

Marker lane
195kDa
140kDa
105kDa
66kDa
52kDa
38kDa
28kDa
20kDa
13kDa
8kDa
Cyto-FOXO3 97kD

Figure 3E

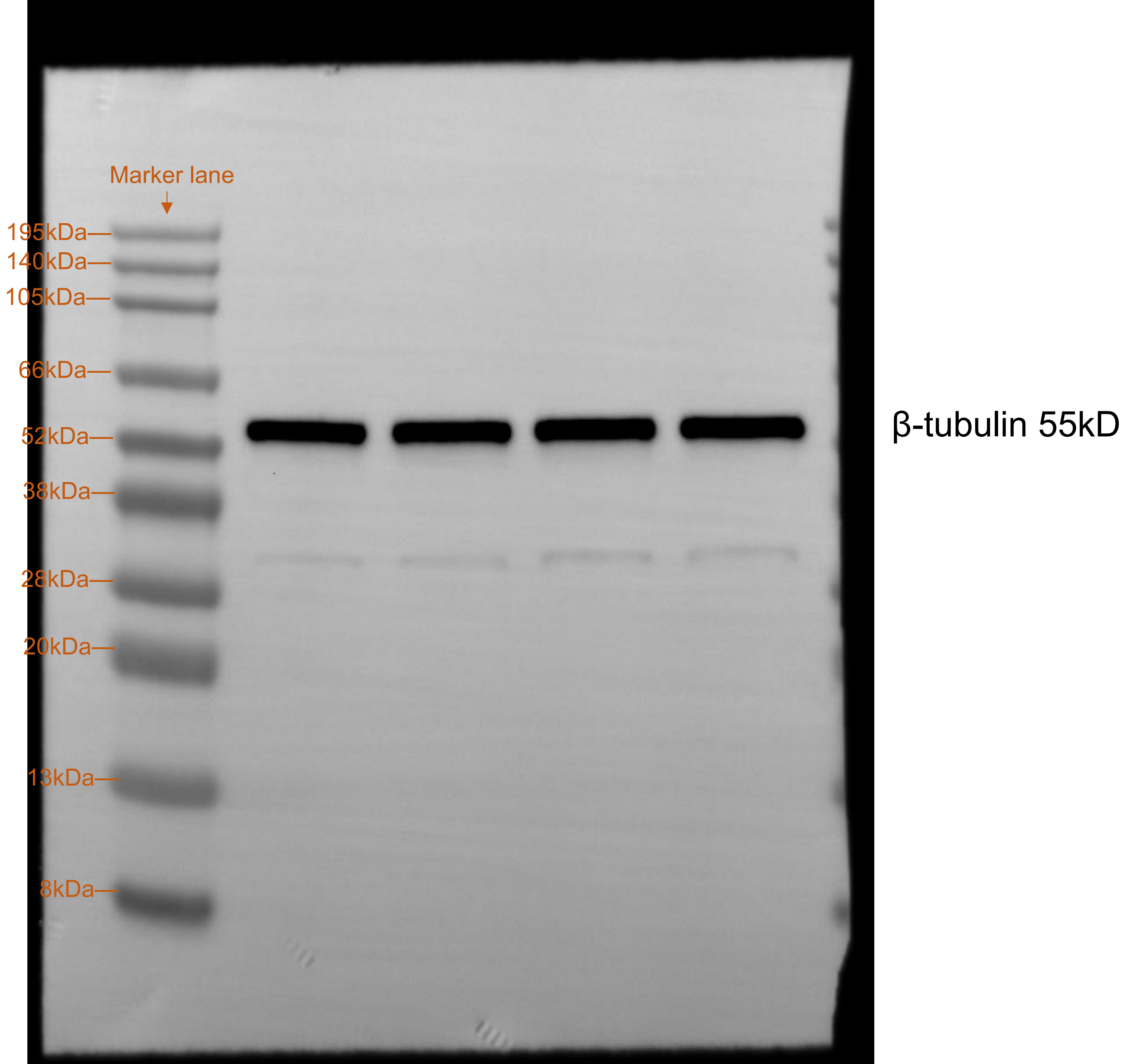

Marker lane
195kDa
140kDa
105kDa
66kDa
52kDa
38kDa
28kDa
20kDa
13kDa
8kDa
β-tubulin 55kD

Figure 4C

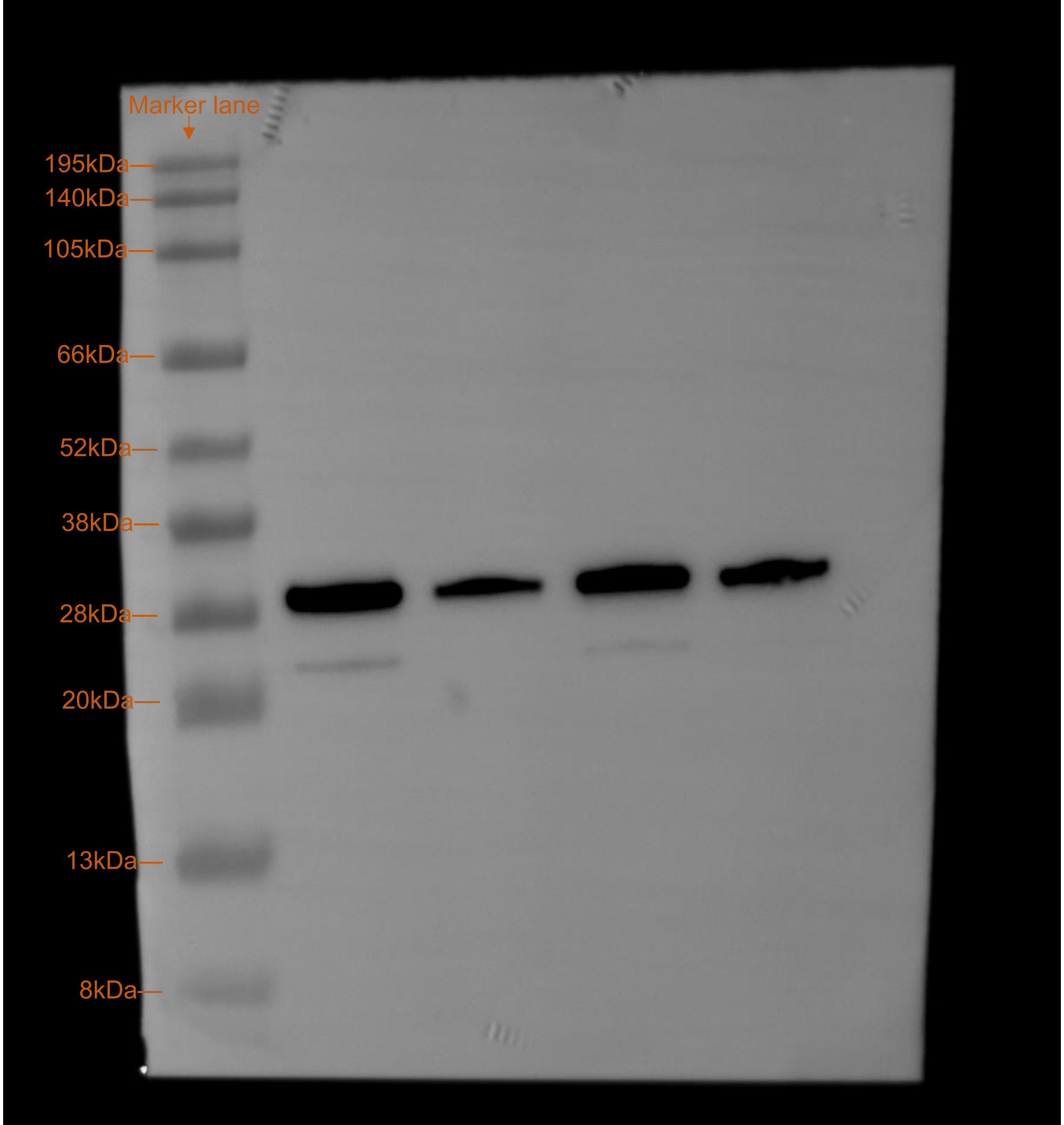
Marker lane
195kDa
140kDa
105kDa
66kDa
52kDa
38kDa
28kDa
20kDa
13kDa
8kDa
TFAM 29kD

Figure 4C

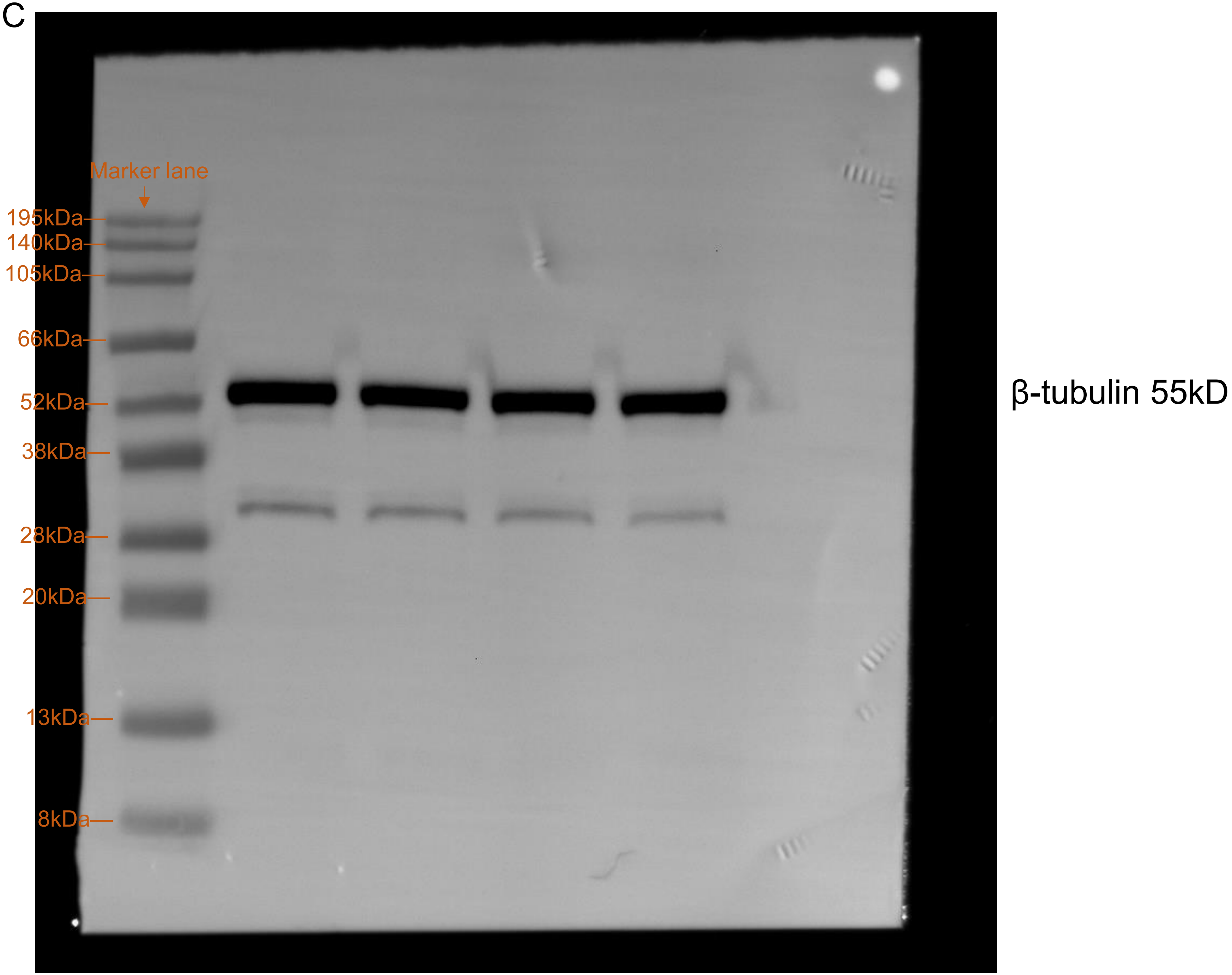
Marker lane
195kDa
140kDa
105kDa
66kDa
52kDa
38kDa
28kDa
20kDa
13kDa
8kDa
β-tubulin 55kD

Figure 4K

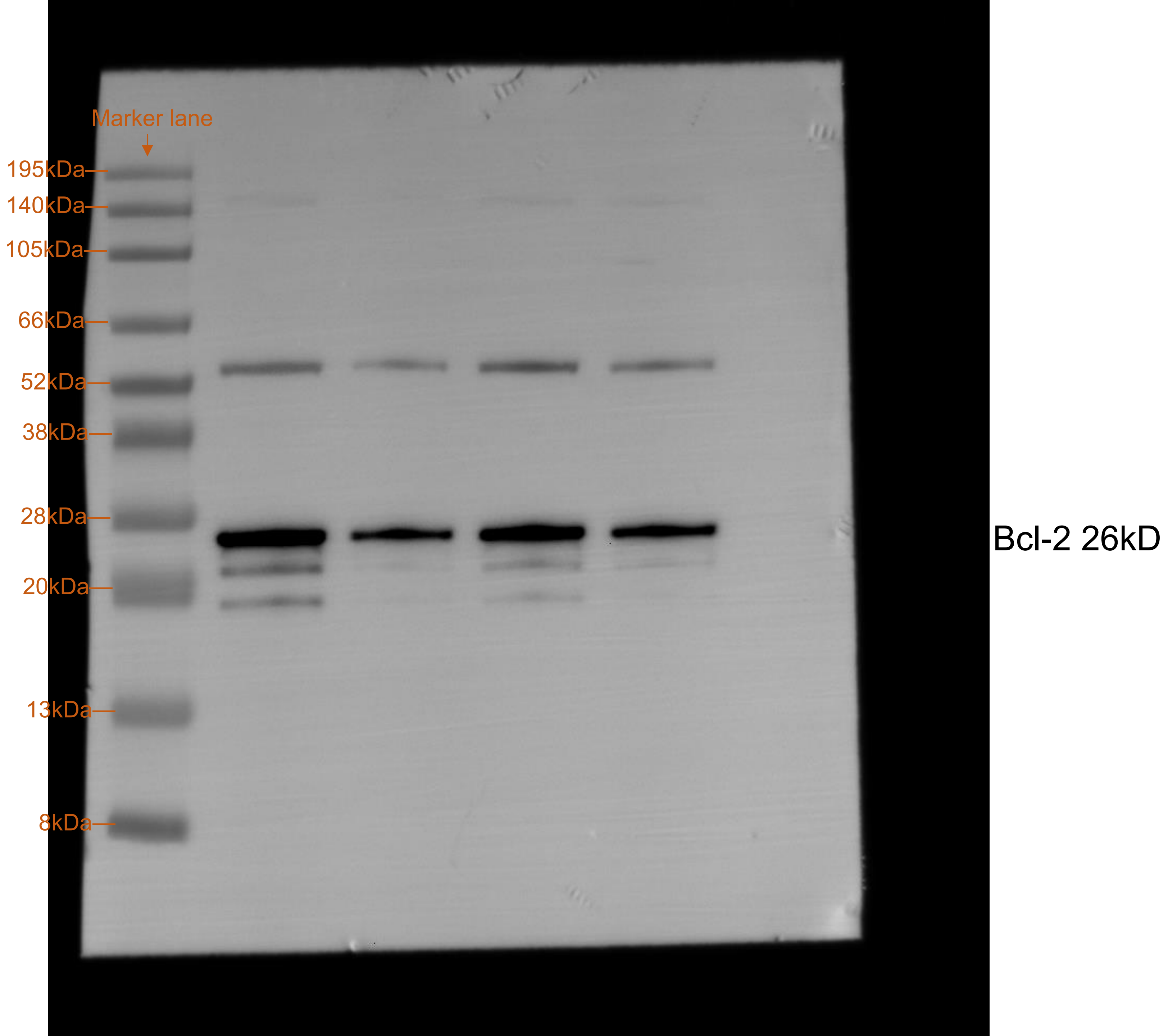
Marker lane
195kDa
140kDa
105kDa
66kDa
52kDa
38kDa
28kDa
20kDa
13kDa
8kDa
Bcl-2 26kD

Figure 4K

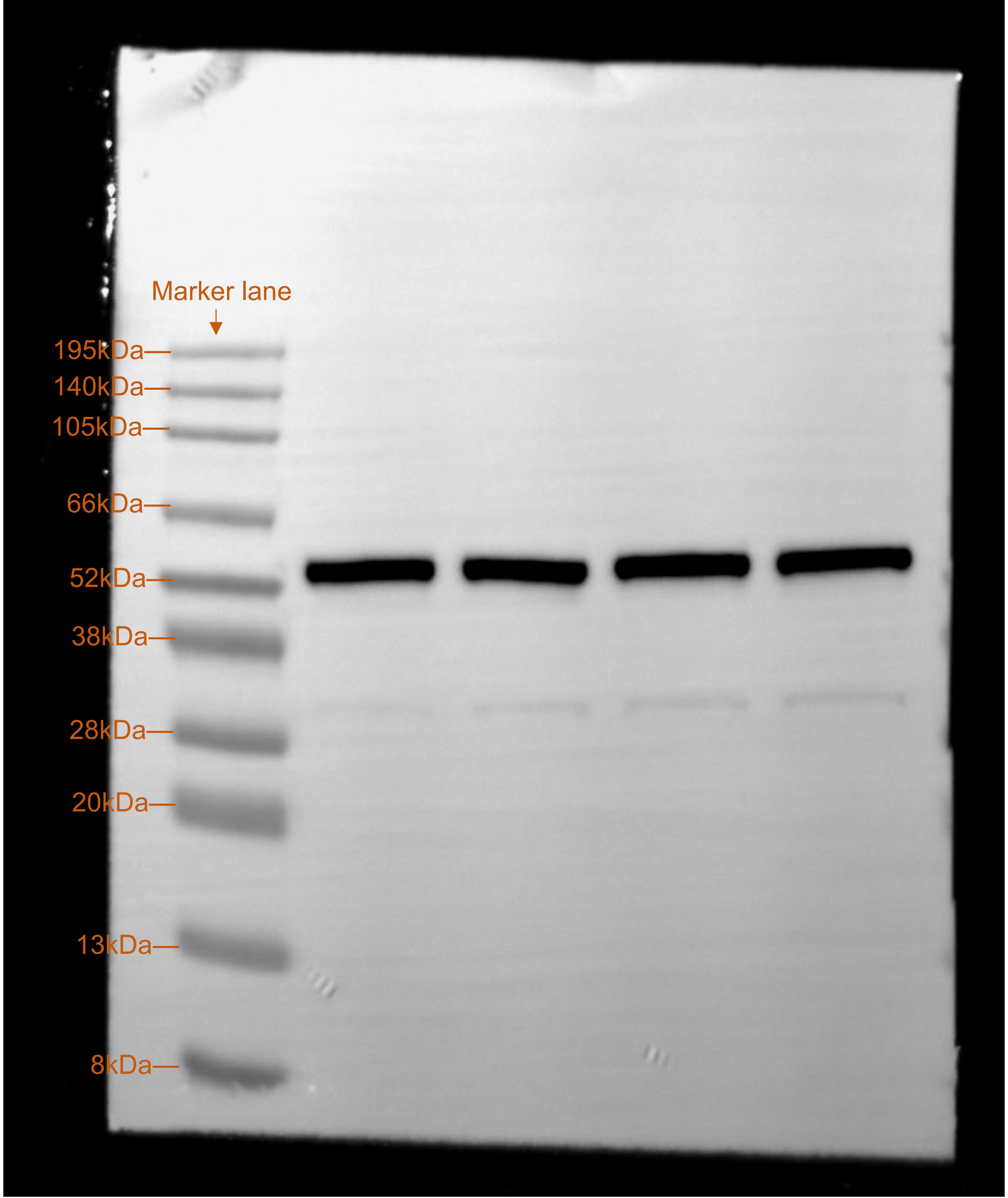

β-tubulin 55kD

Figure 4K

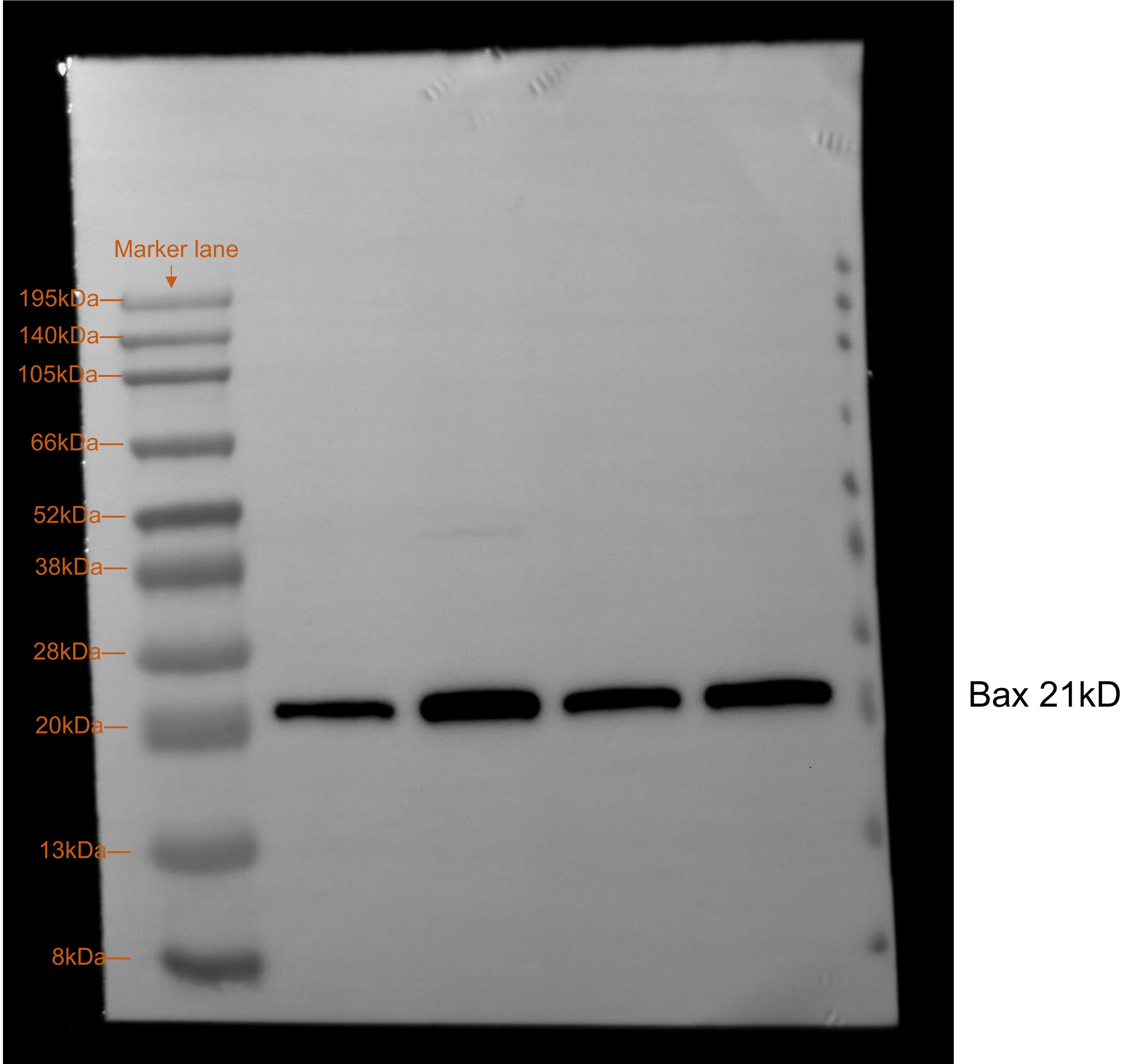
Marker lane
195kDa
140kDa
105kDa
66kDa
52kDa
38kDa
28kDa
20kDa
13kDa
8kDa
Bax 21kD

Figure 4K

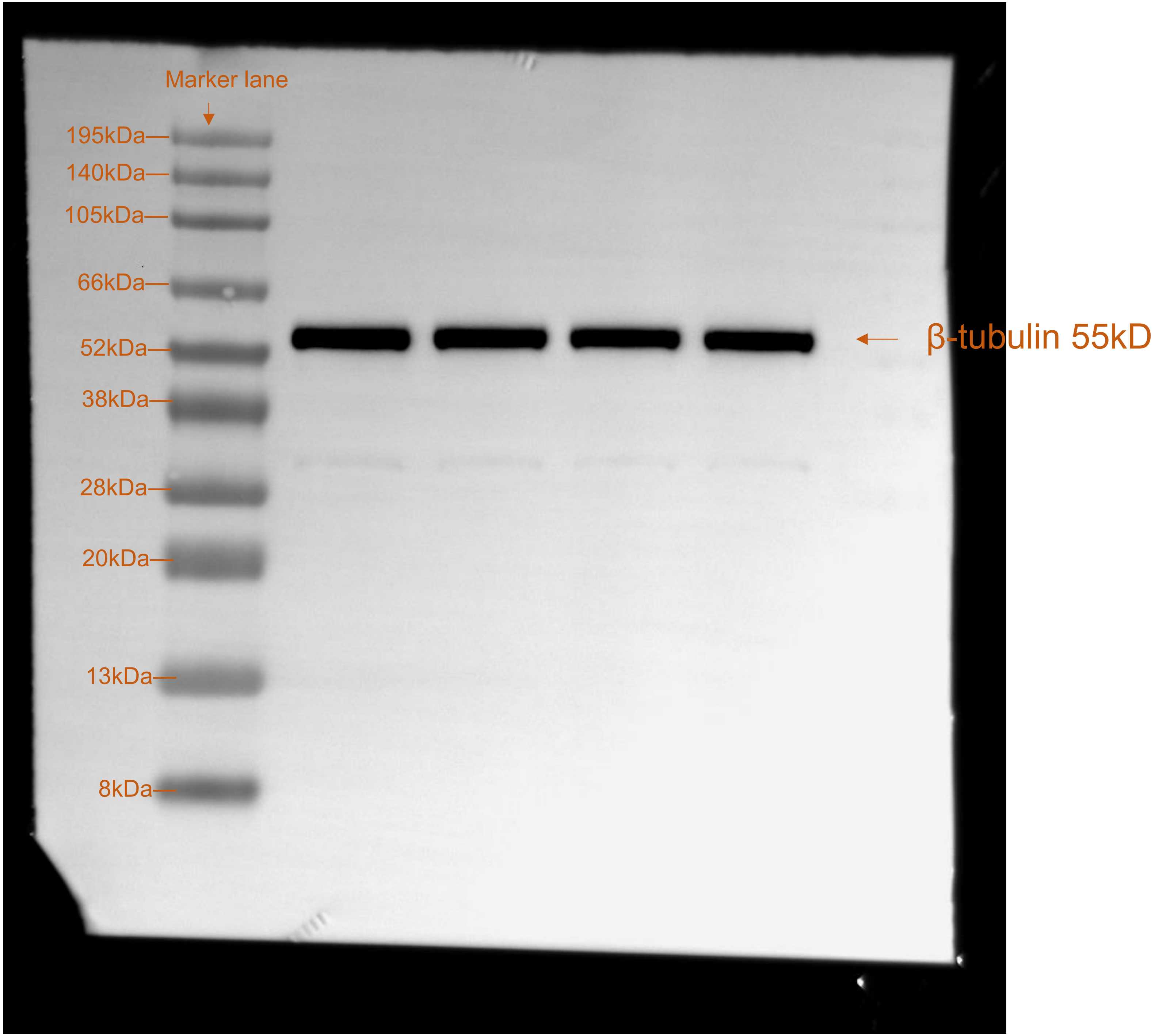

Marker lane
195kDa
140kDa
105kDa
66kDa
52kDa
38kDa
28kDa
20kDa
13kDa
8kDa
β-tubulin 55kD

Figure 5A

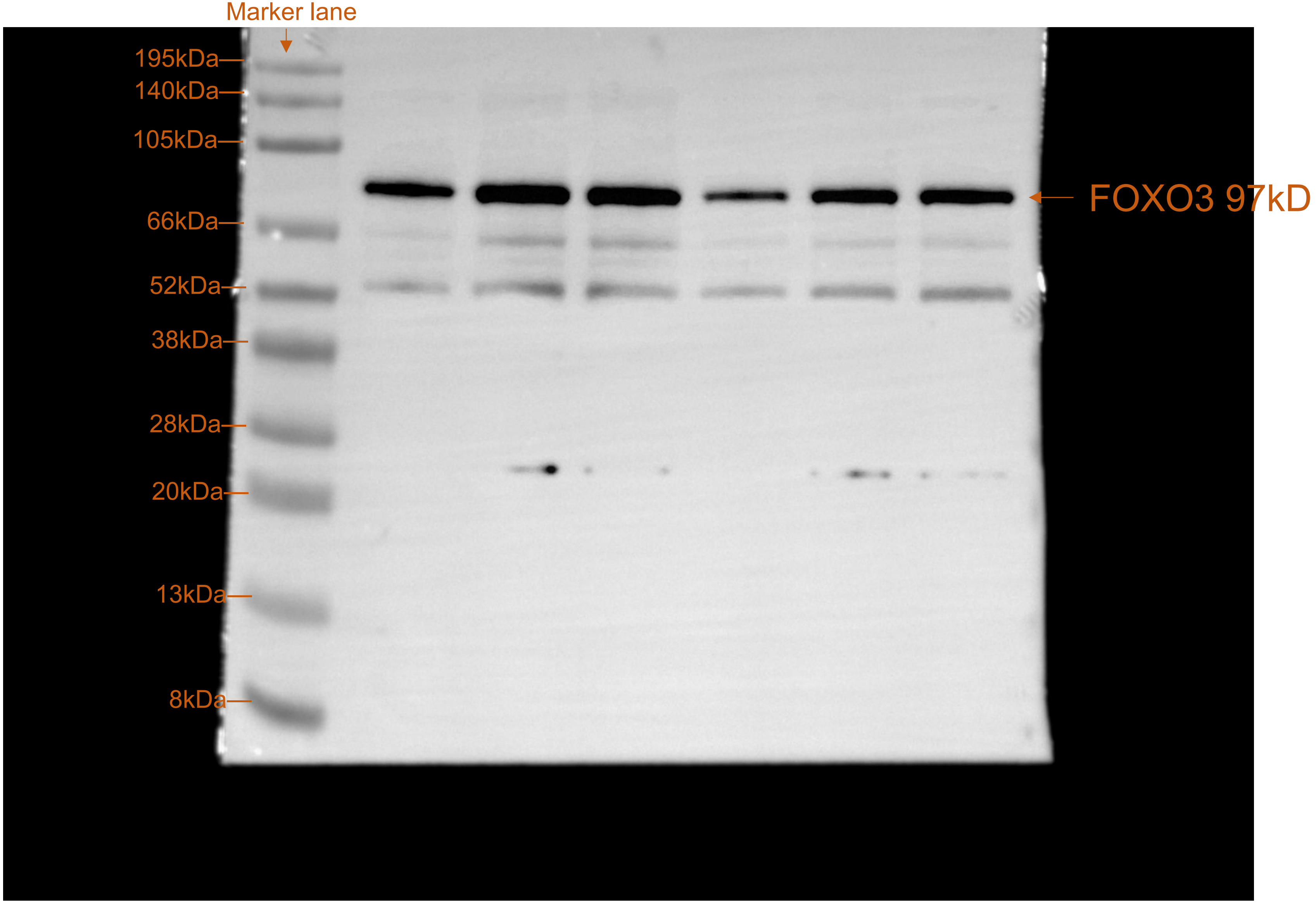

Marker lane
195kDa
140kDa
105kDa
66kDa
52kDa
38kDa
28kDa
20kDa
13kDa
8kDa
FOXO3 97kD

Figure 5A

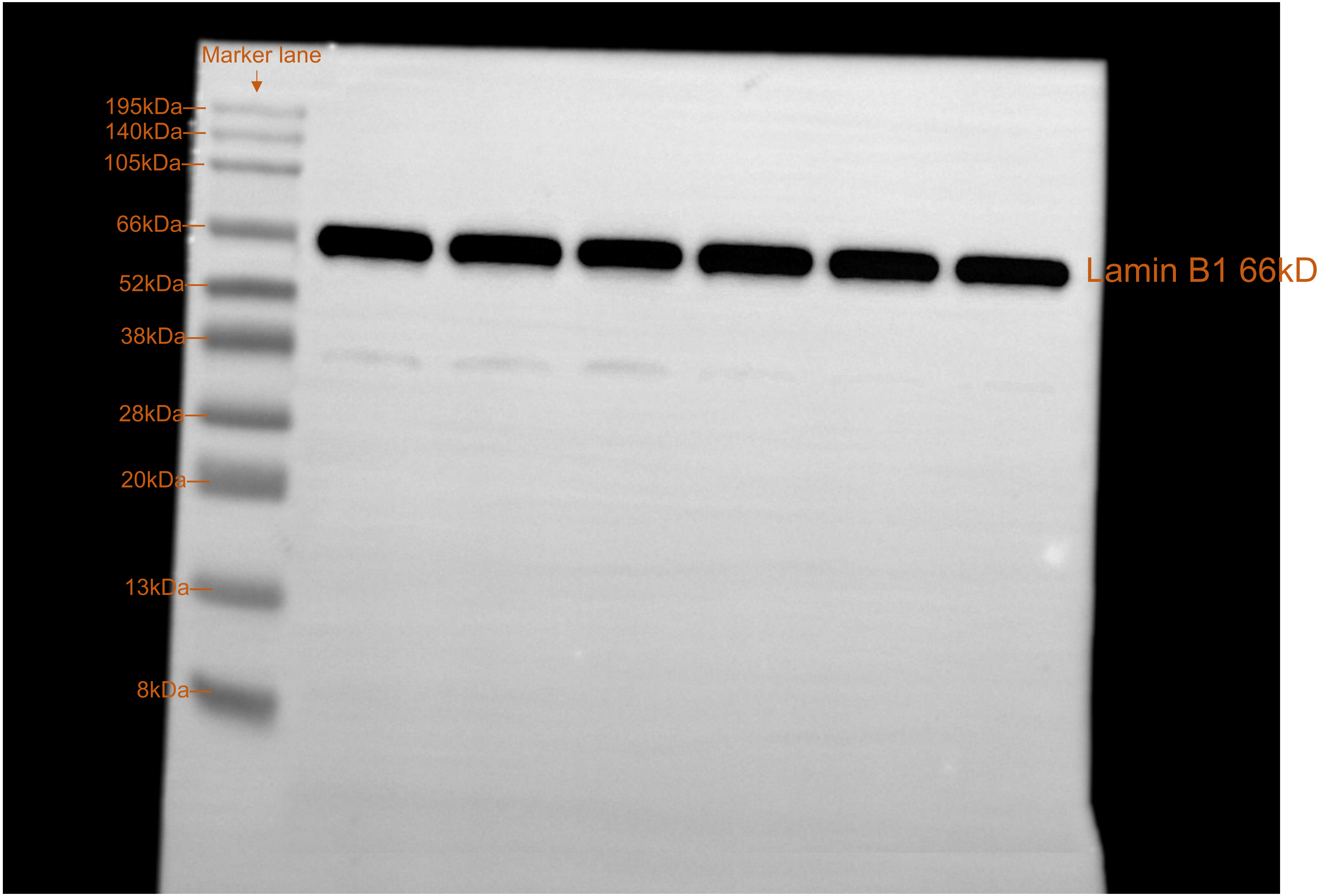

Marker lane
195kDa
140kDa
105kDa
66kDa
52kDa
38kDa
28kDa
20kDa
13kDa
8kDa
Lamin B1 66kD

Figure 5C

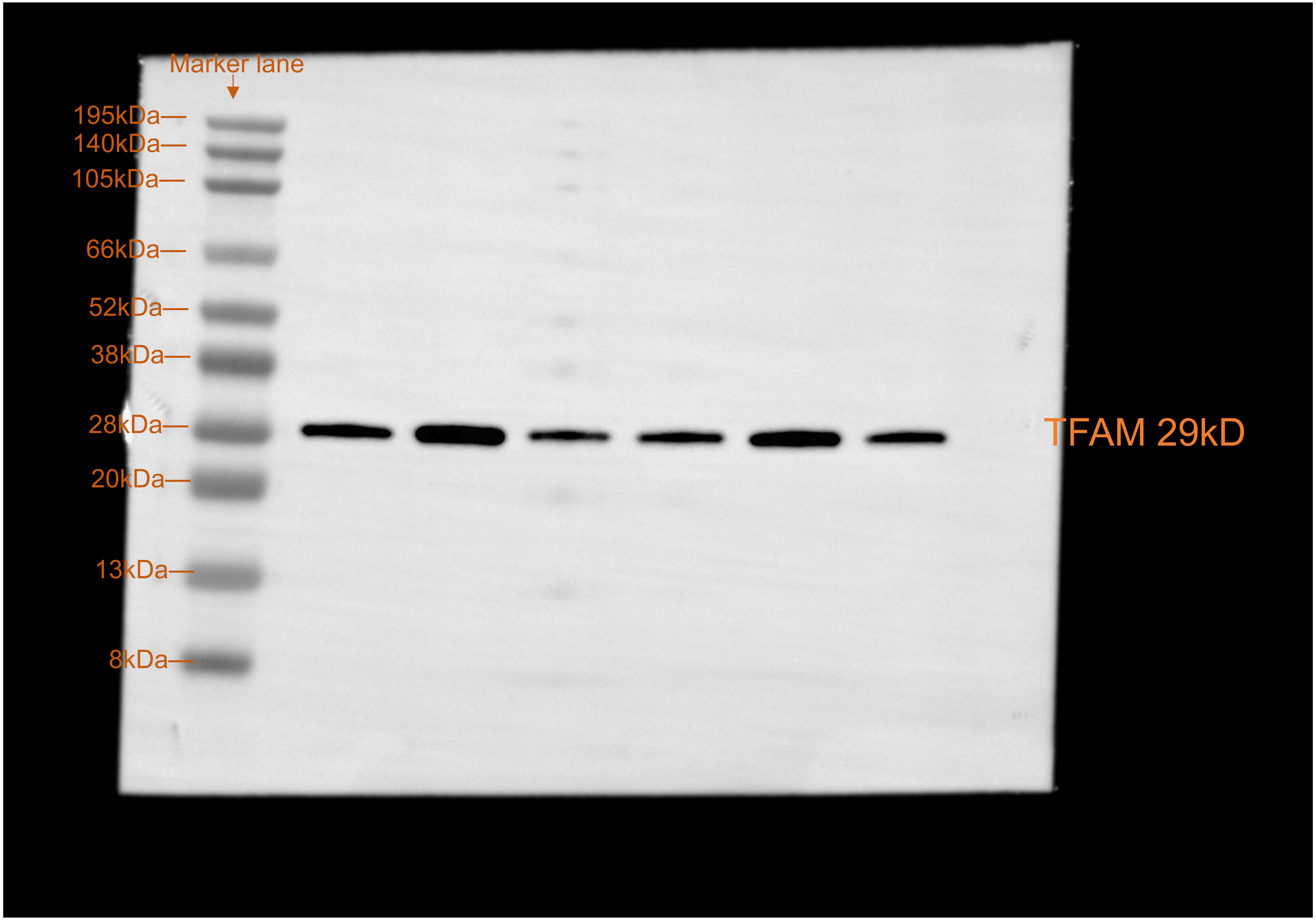
Marker lane
195kDa
140kDa
105kDa
66kDa
52kDa
38kDa
28kDa
20kDa
13kDa
8kDa
TFAM 29kD

Figure 5C

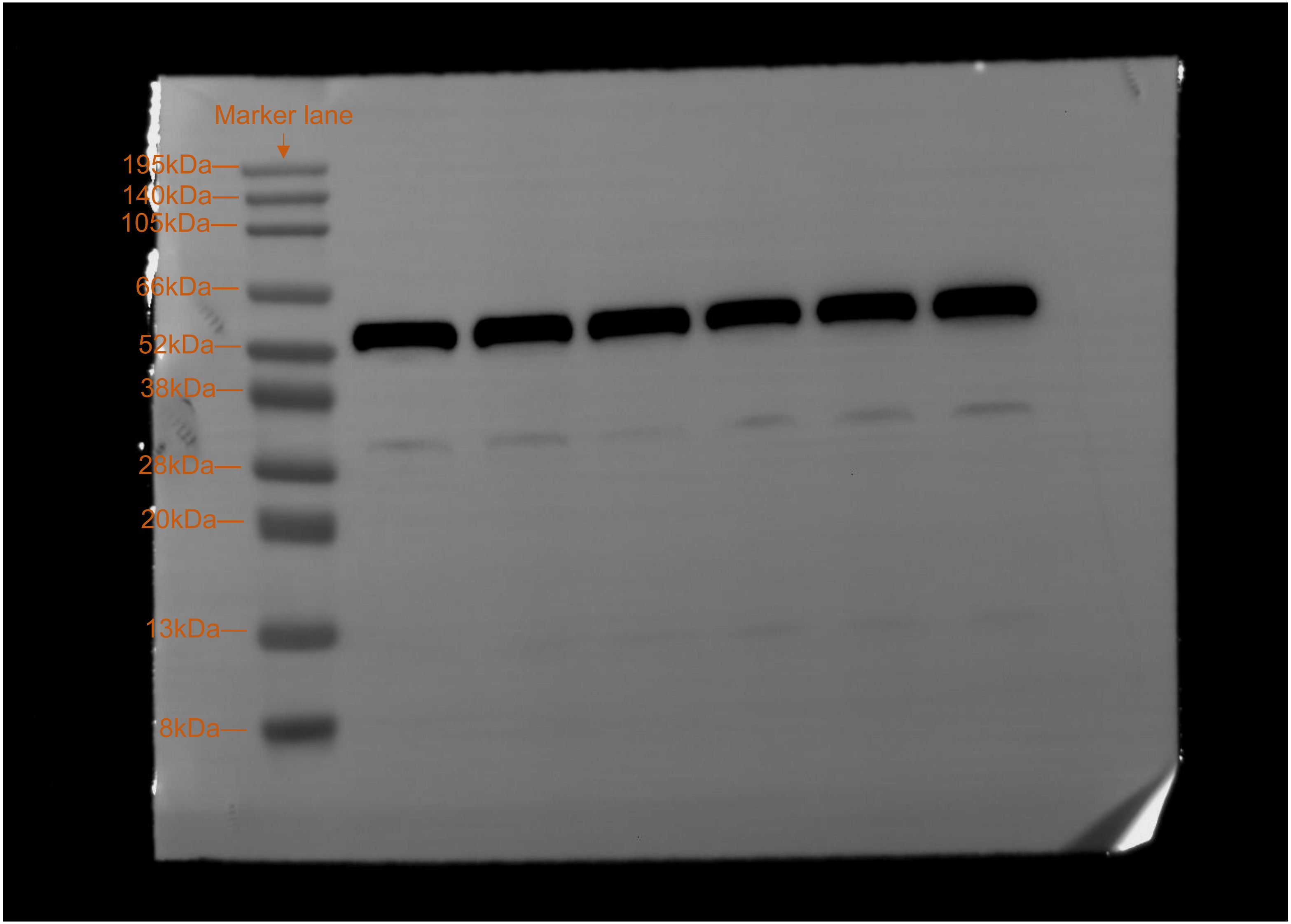

β-tubulin 55kD

Figure 5K

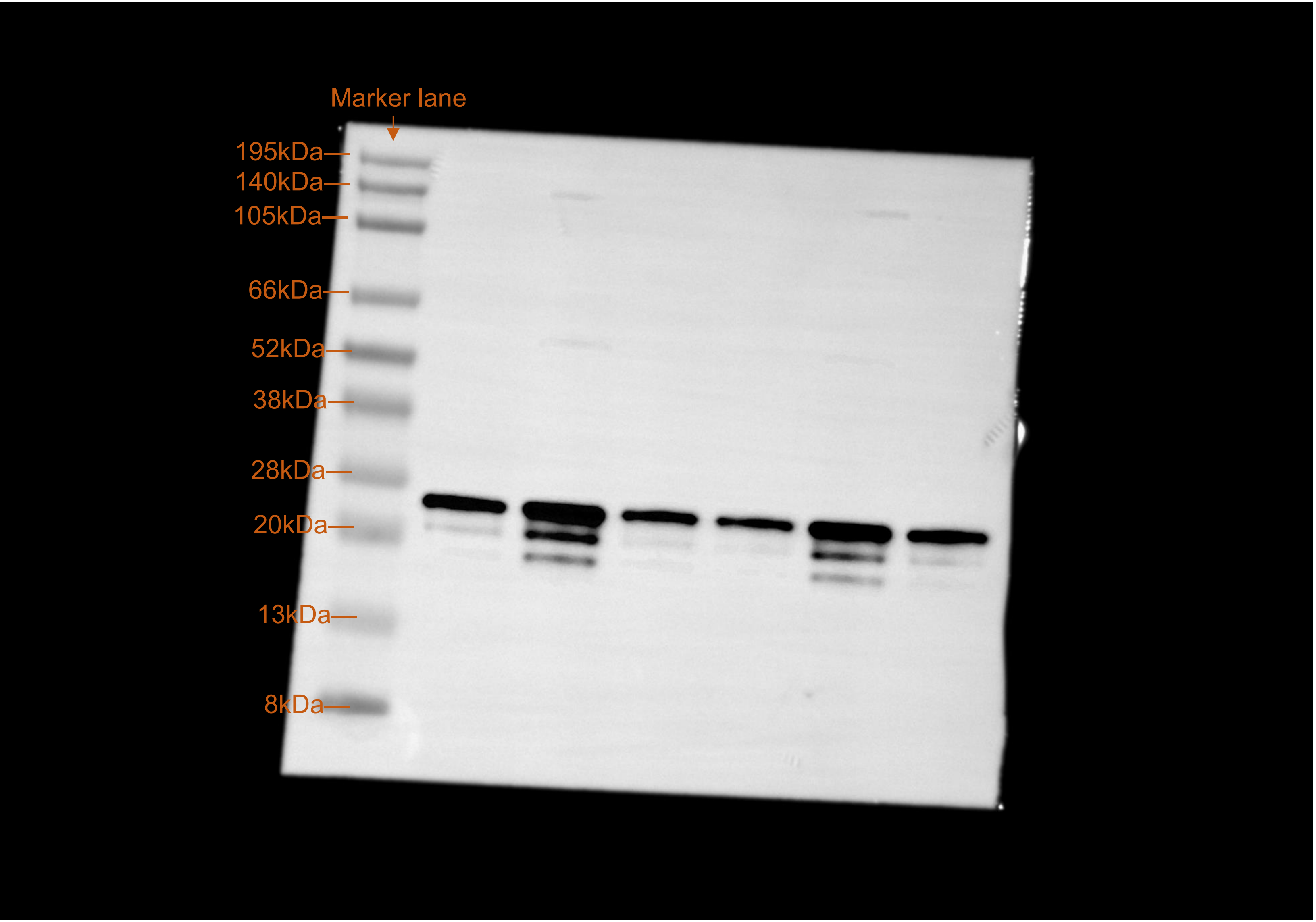

Bcl-2 26kD

Figure 5K

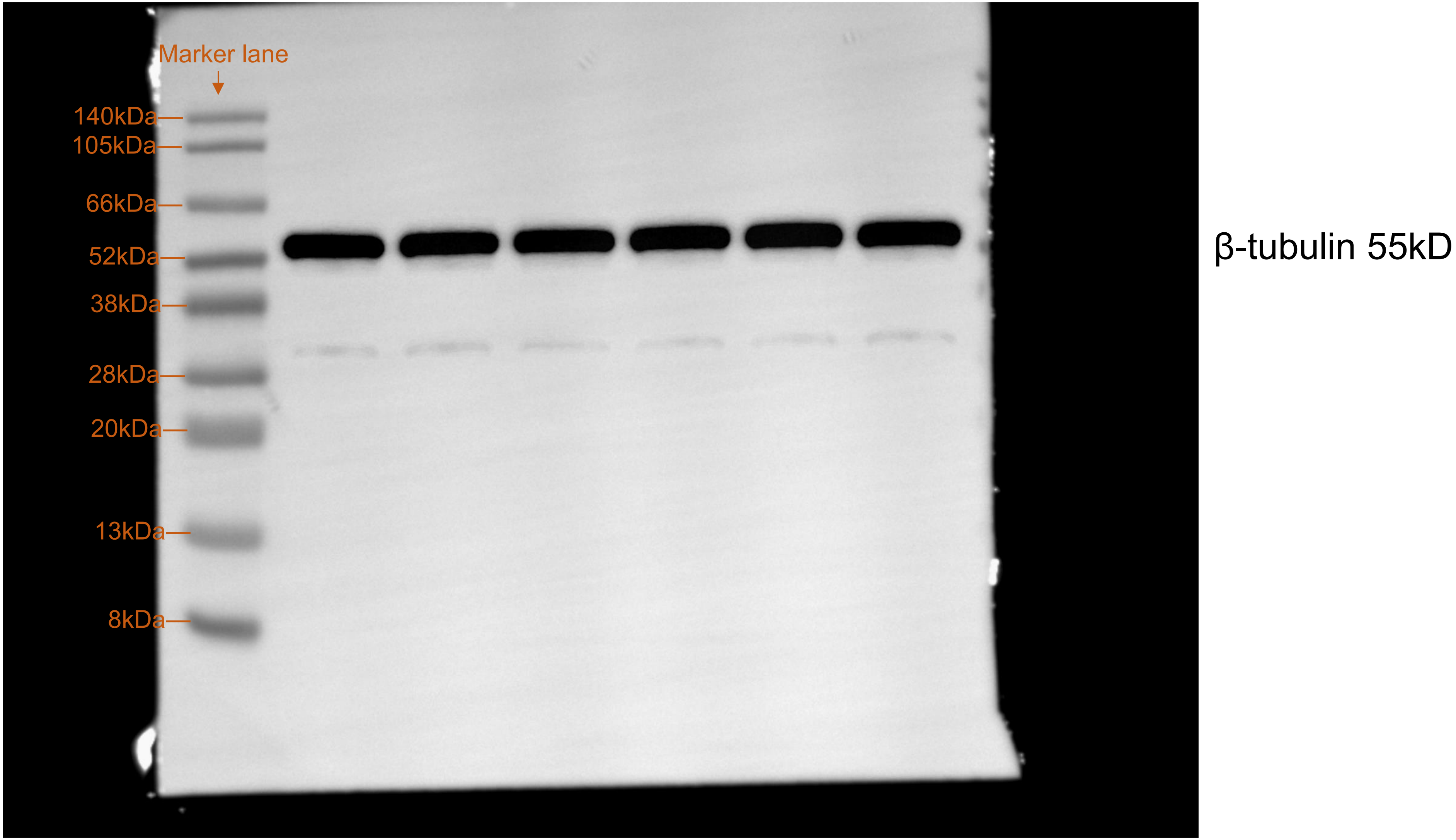

Marker lane
140kDa
105kDa
66kDa
52kDa
38kDa
28kDa
20kDa
13kDa
8kDa
β-tubulin 55kD

Figure 5K

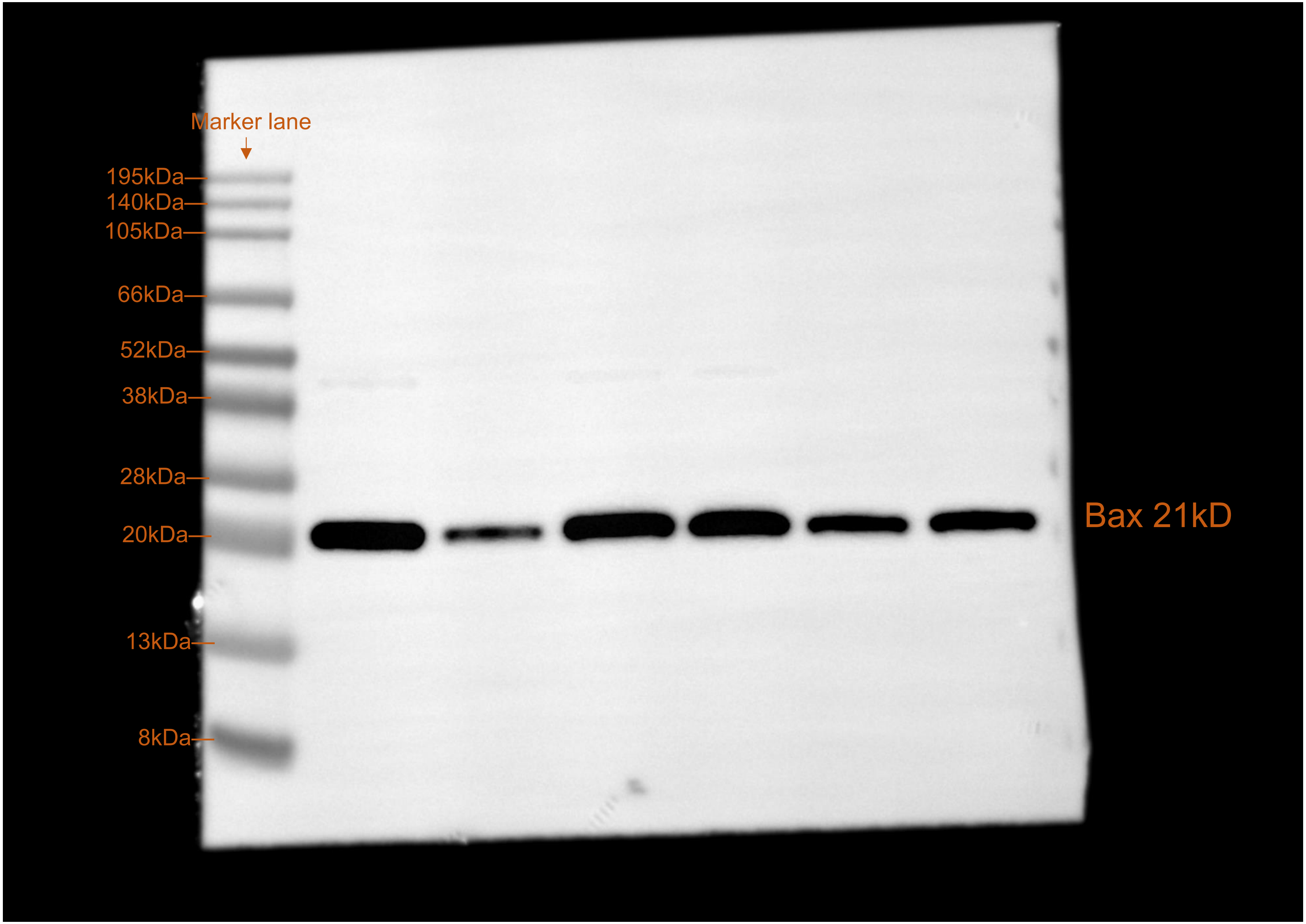
Marker lane
195kDa
140kDa
105kDa
66kDa
52kDa
38kDa
28kDa
20kDa
13kDa
8kDa
Bax 21kD

Figure 5K

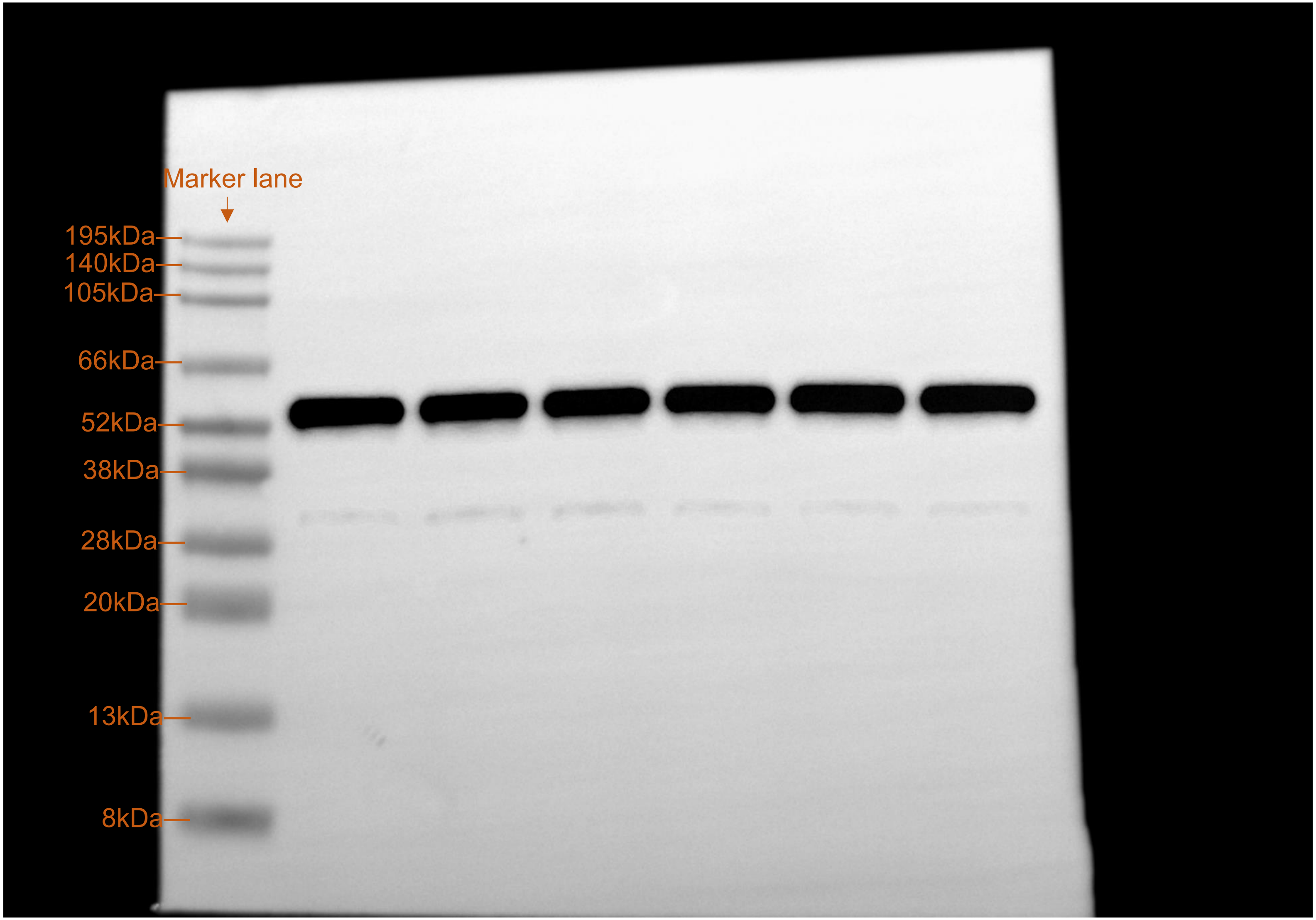

β-tubulin 55kD

Figure 6C

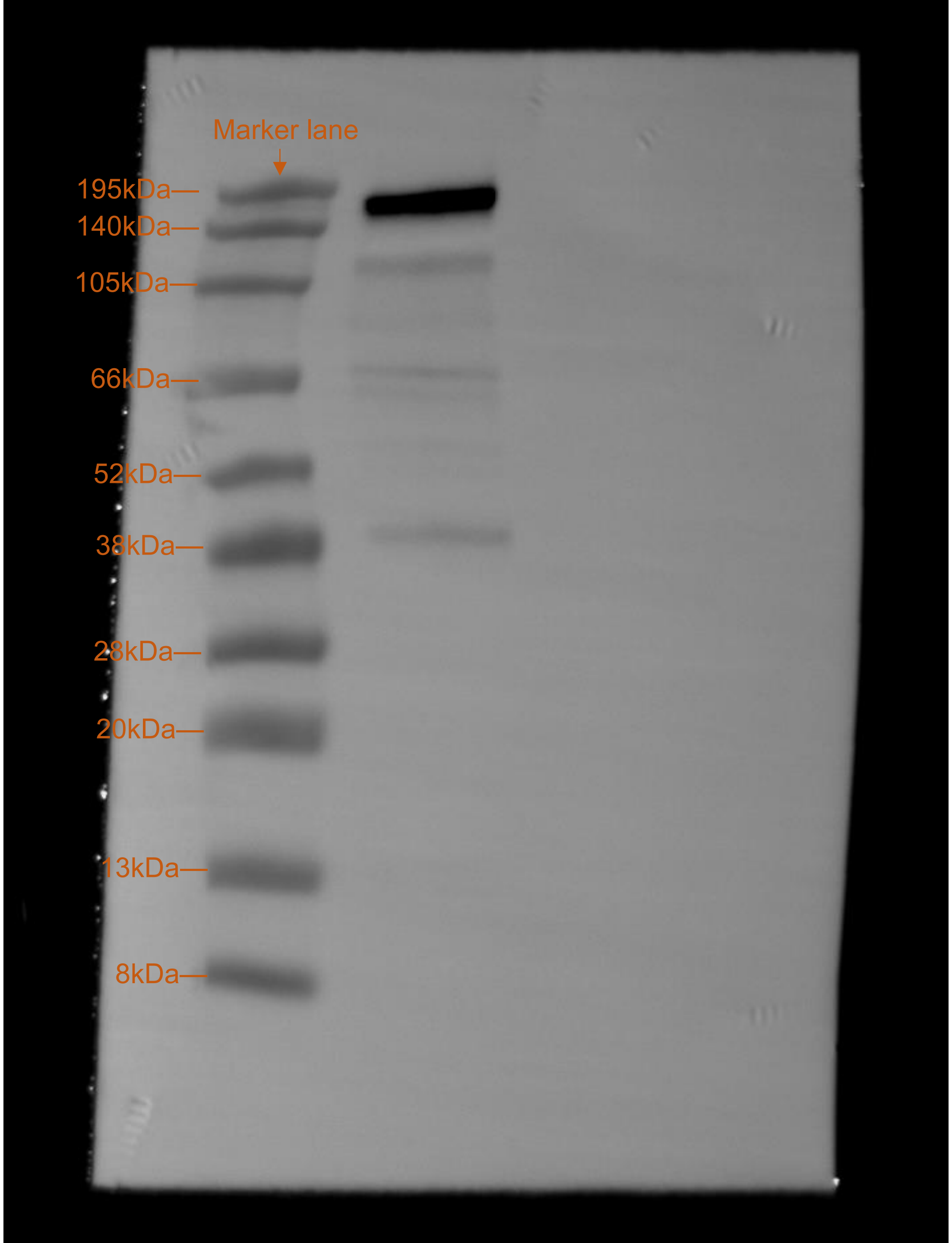

NRIP1 150kD

Figure 6C

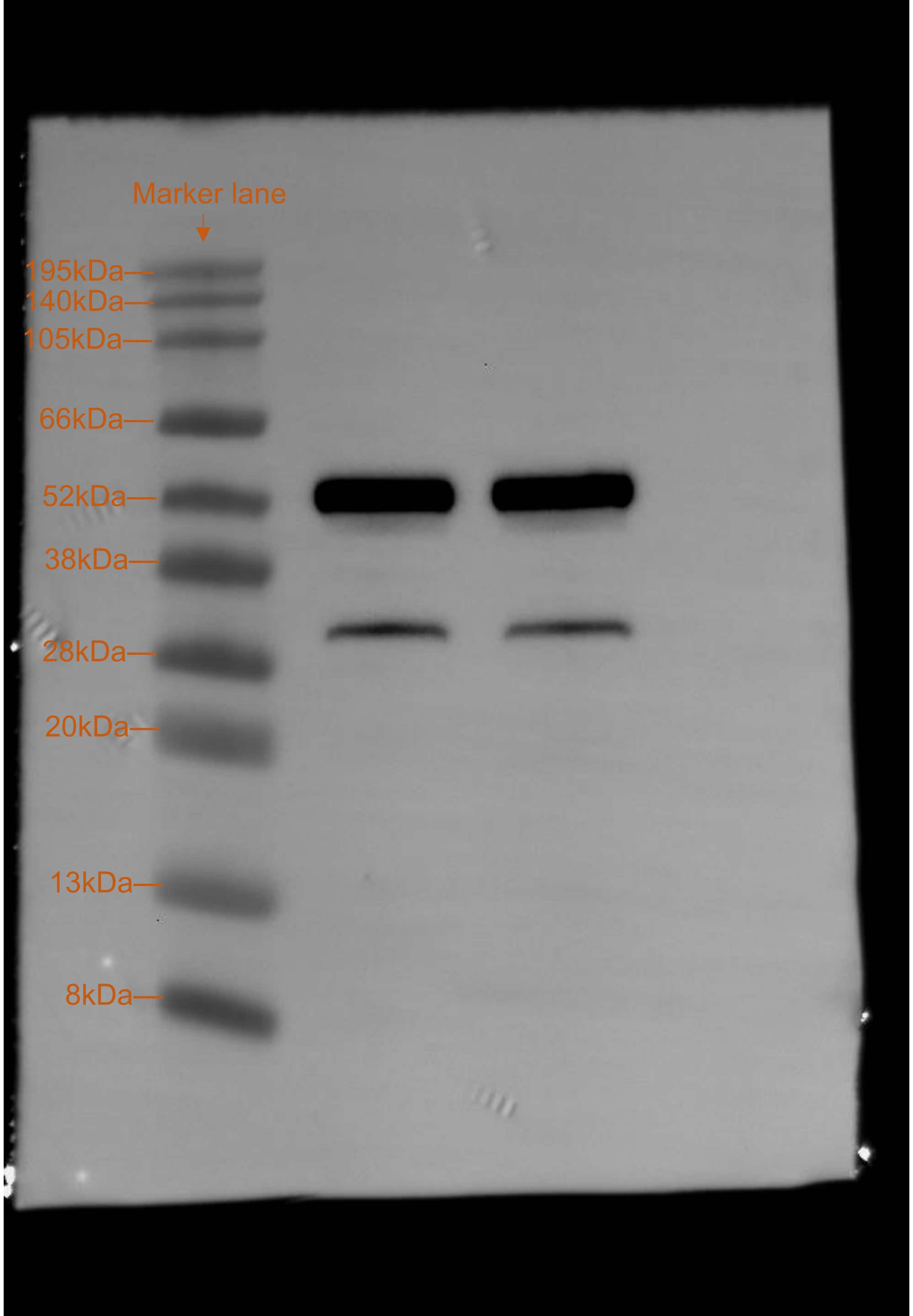

β-tubulin 55kD

Figure 6C

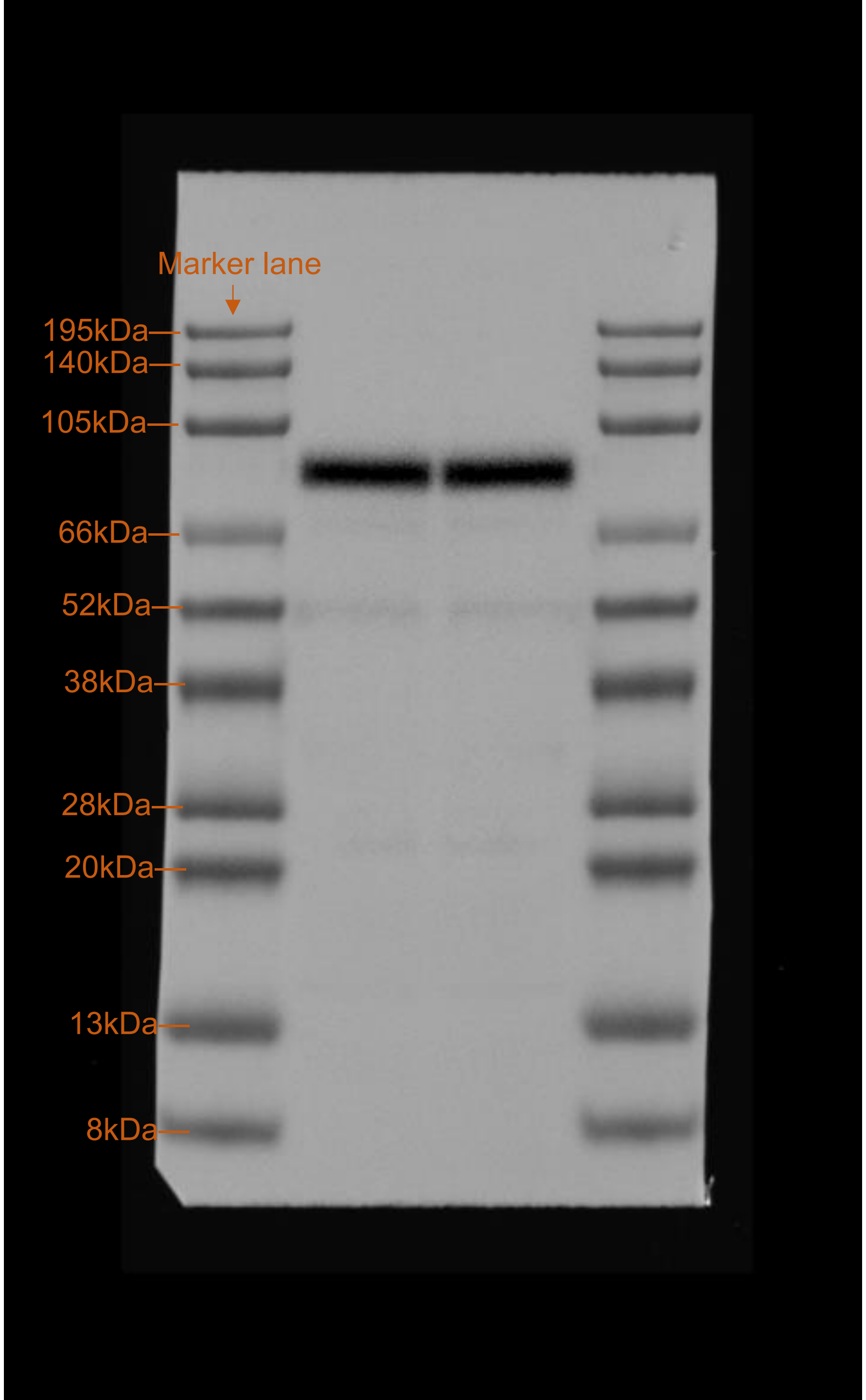

FOXO3 97kD

Figure 6C

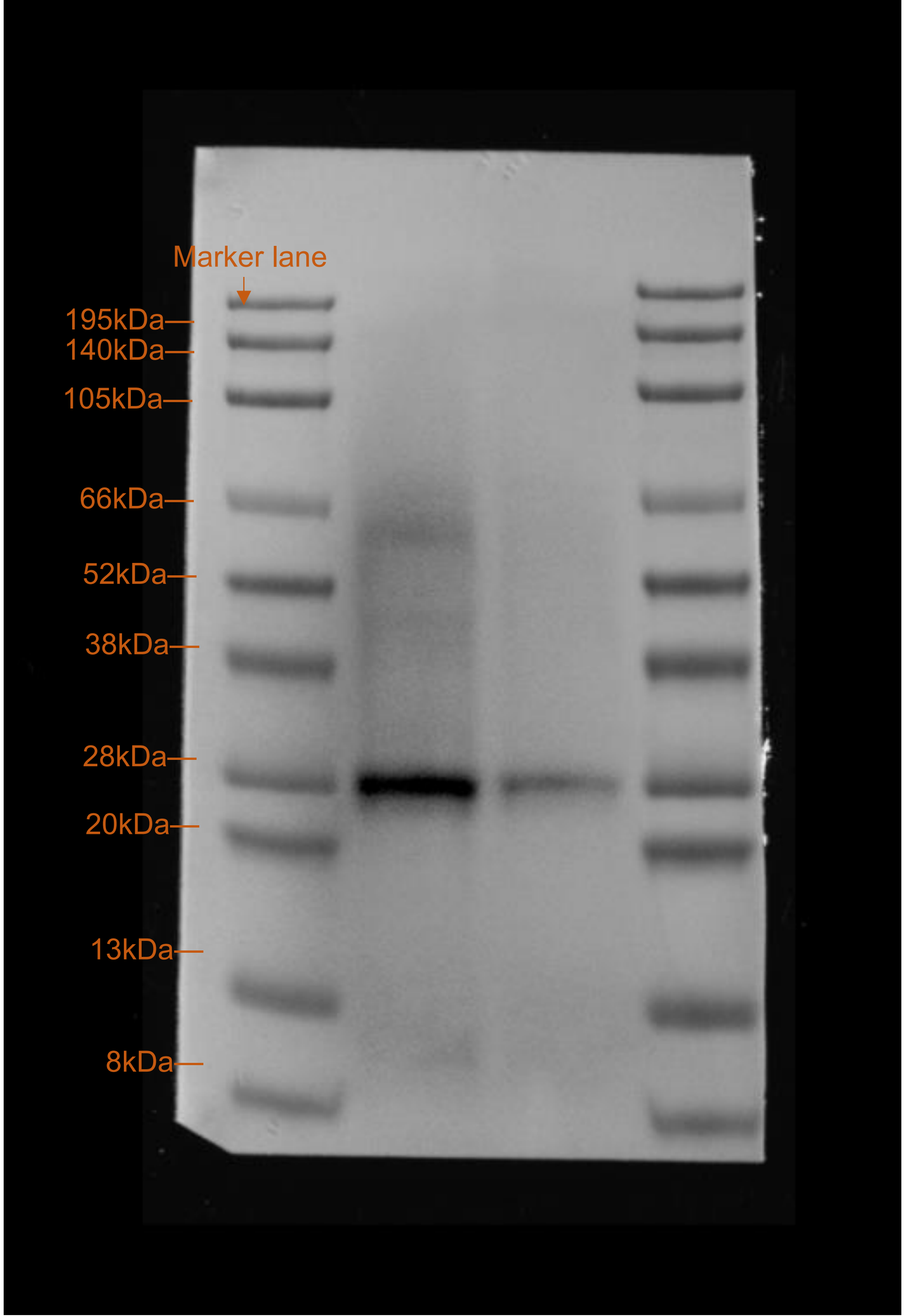

TFAM 29kD

Figure 6C

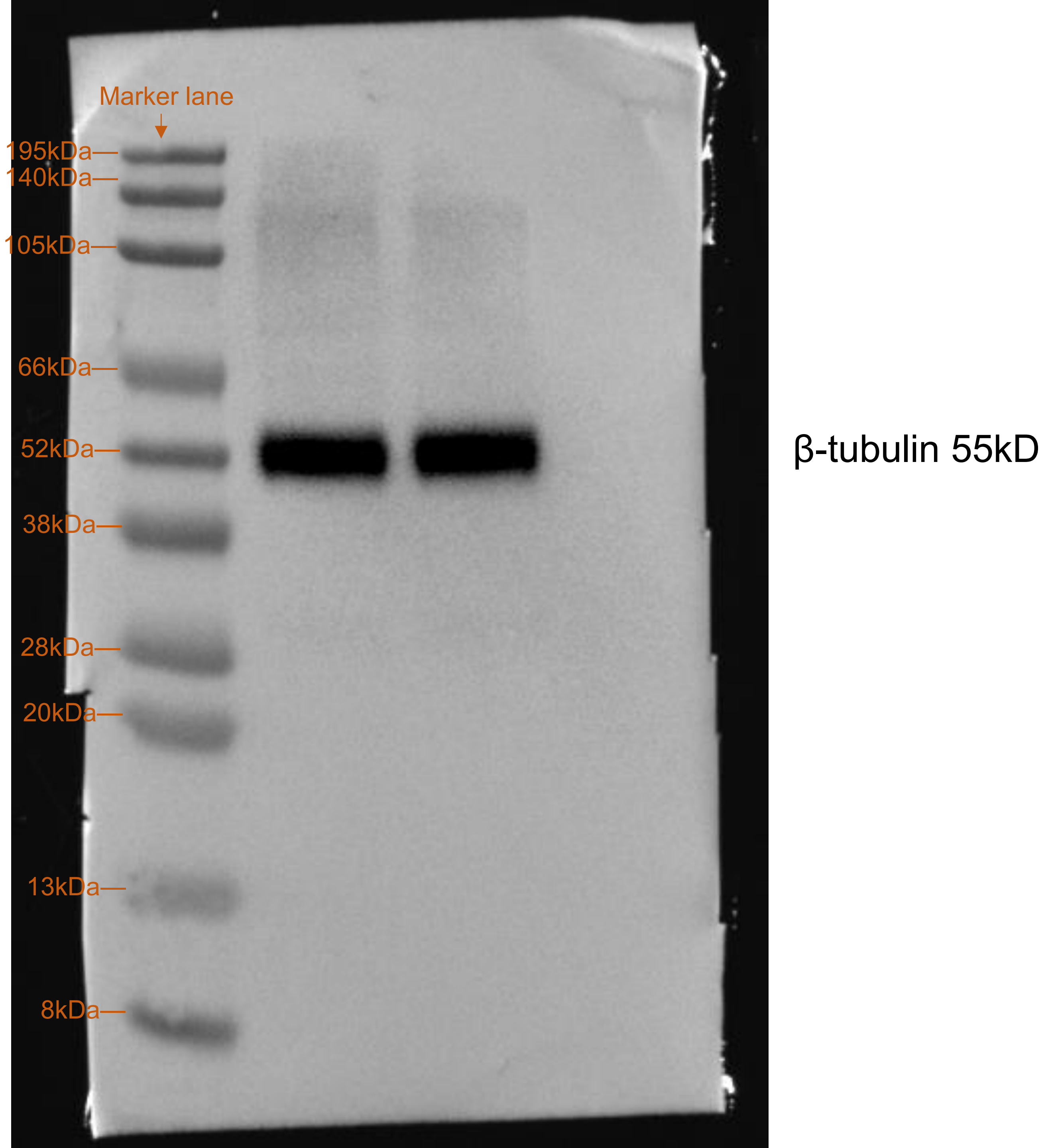
Marker lane
195kDa
140kDa
105kDa
66kDa
52kDa
38kDa
28kDa
20kDa
13kDa
8kDa
β-tubulin 55kD

Figure 6E

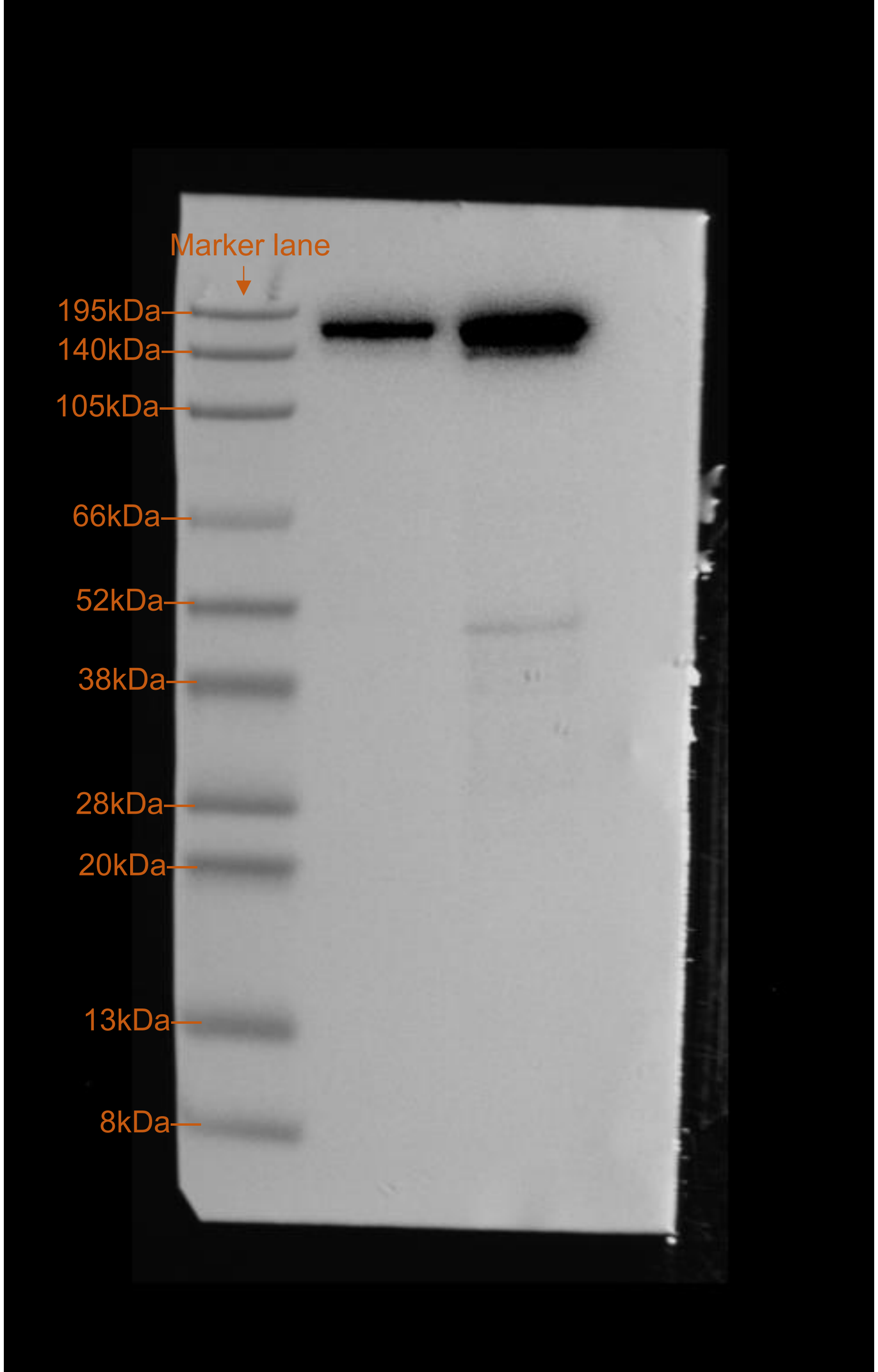

NRIP1 150kD

Figure 6E

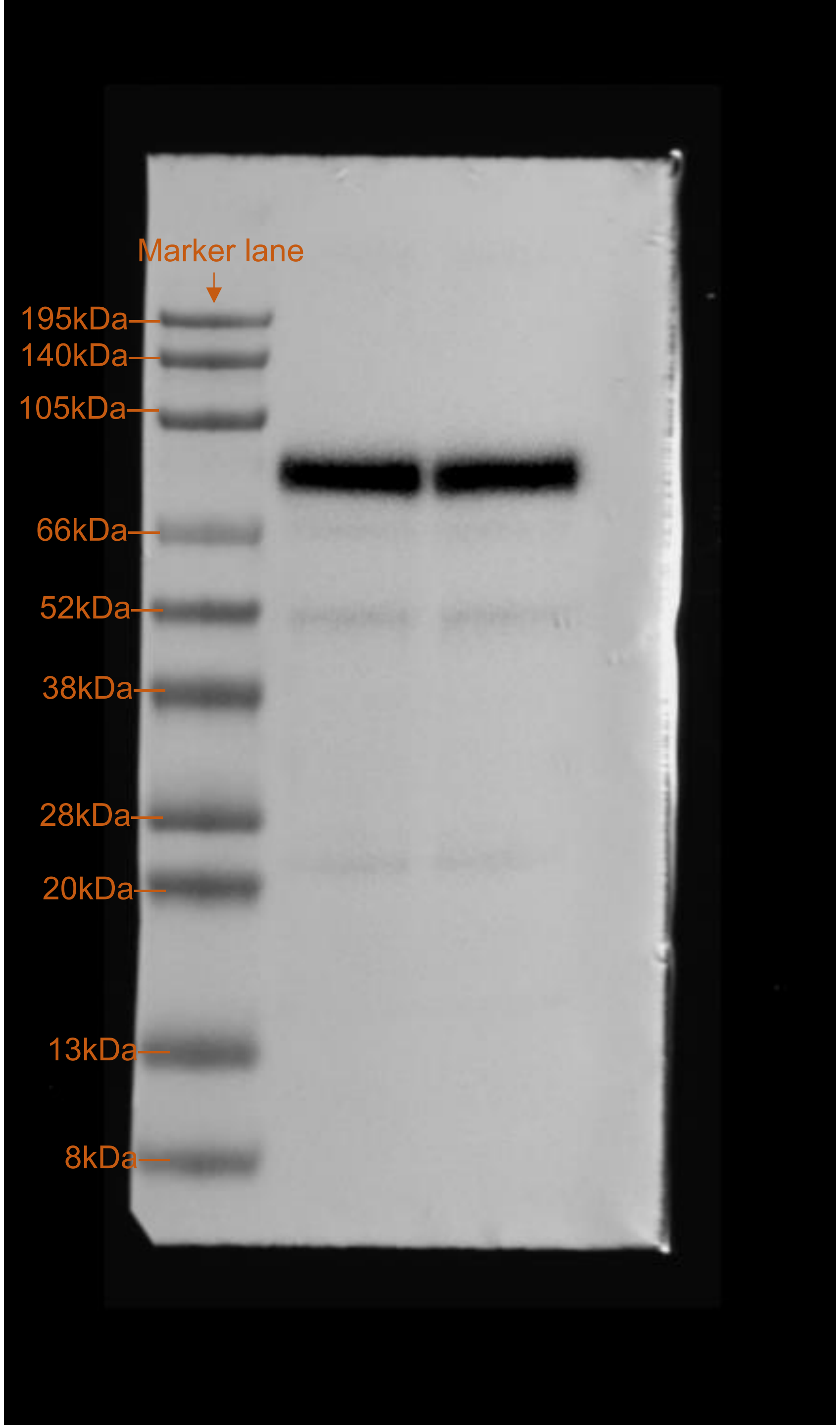

FOXO3 97kD

Figure 6E

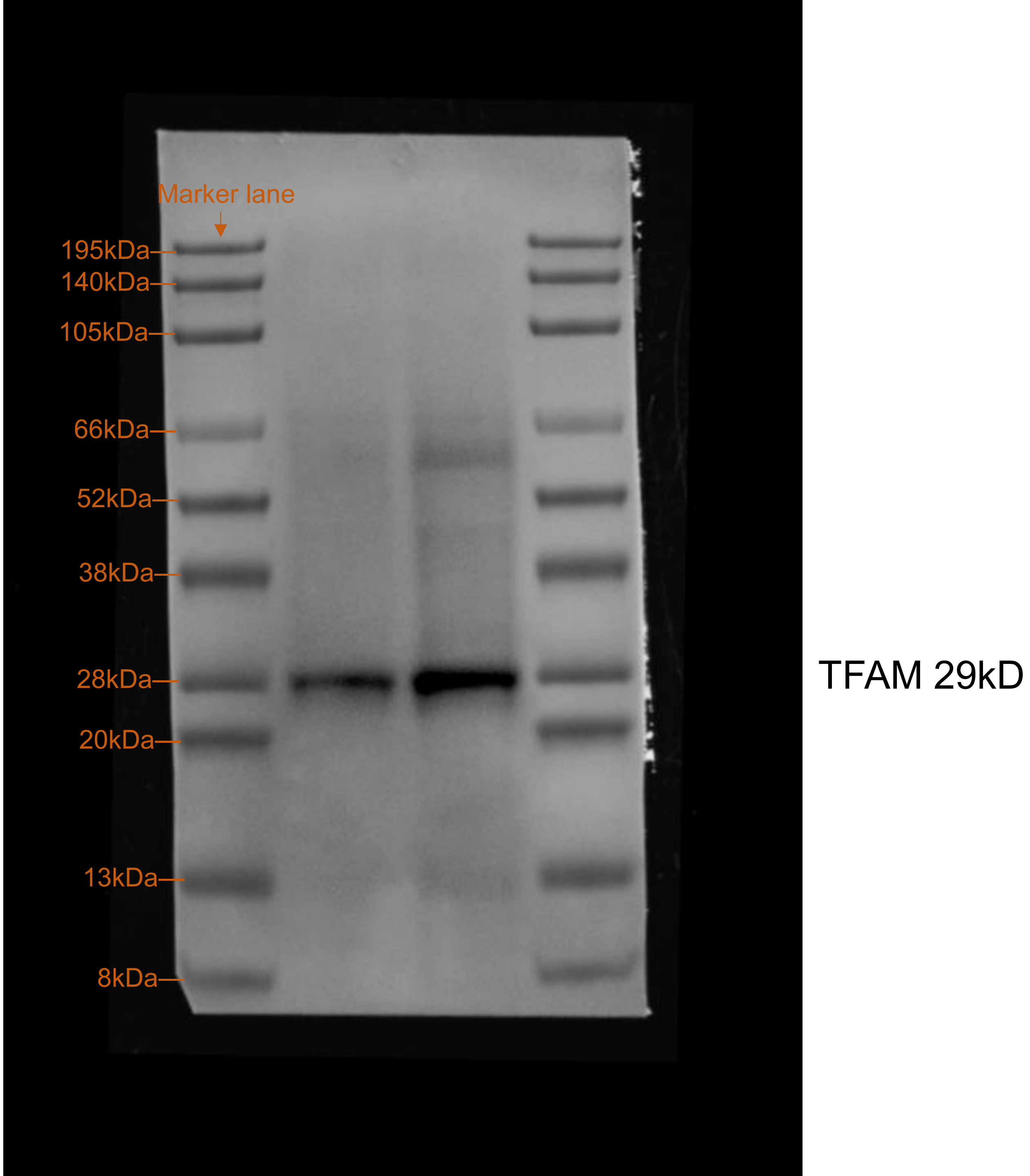
Marker lane
195kDa
140kDa
105kDa
66kDa
52kDa
38kDa
28kDa
20kDa
13kDa
8kDa
TFAM 29kD

Figure 6E

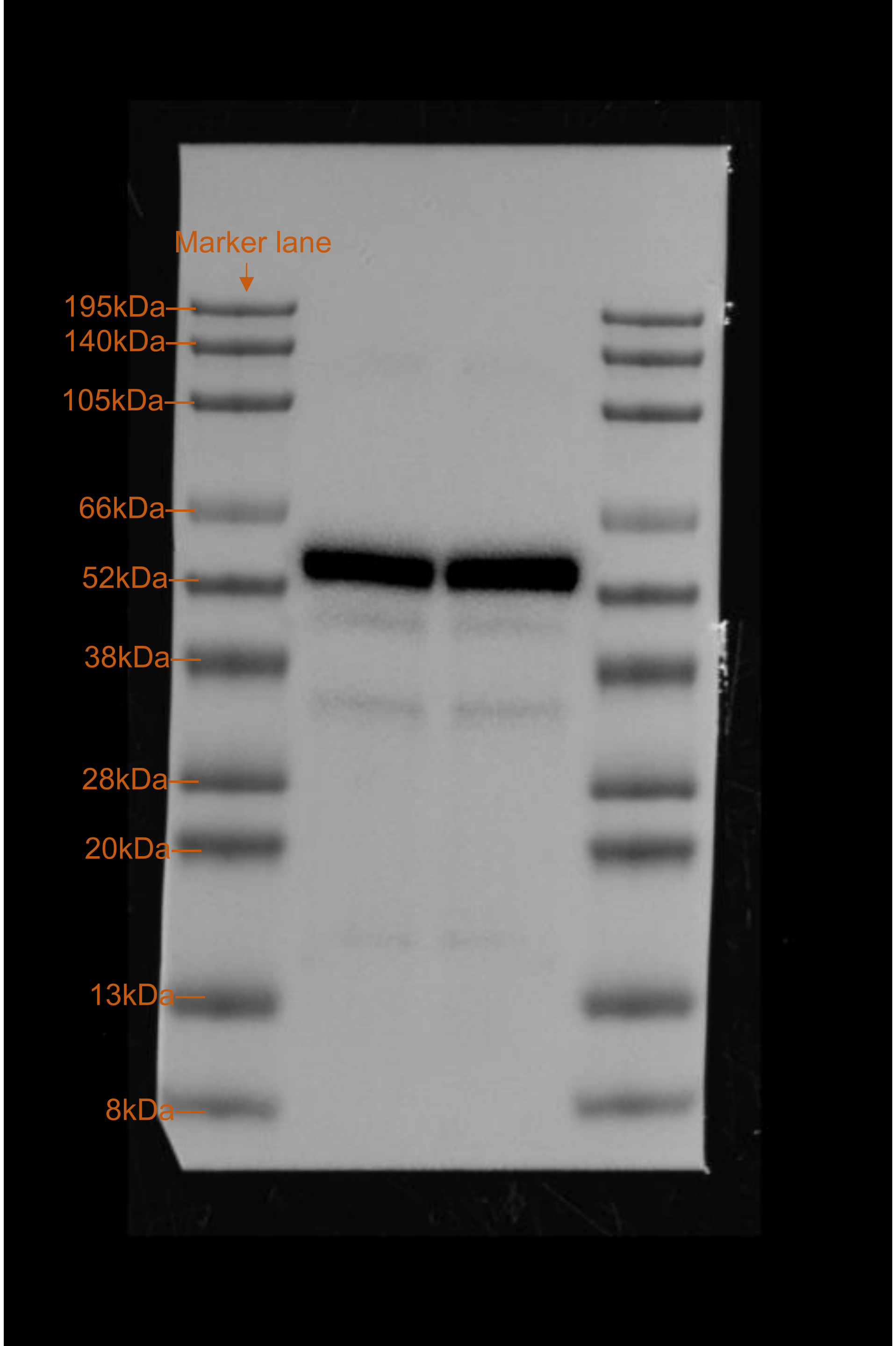

β-tubulin 55kD

Figure 6G

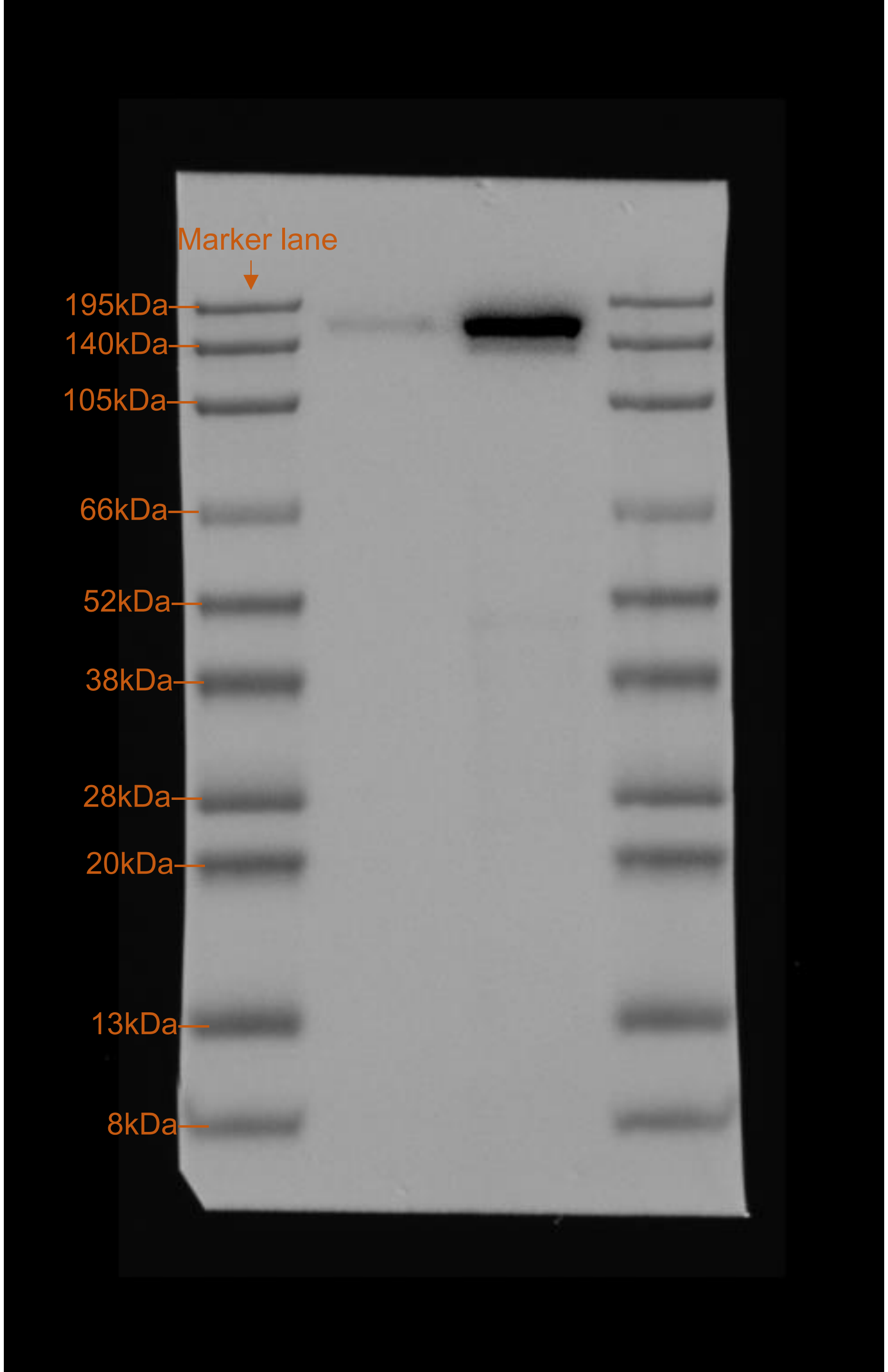

NRIP1 150kD

Figure 6G

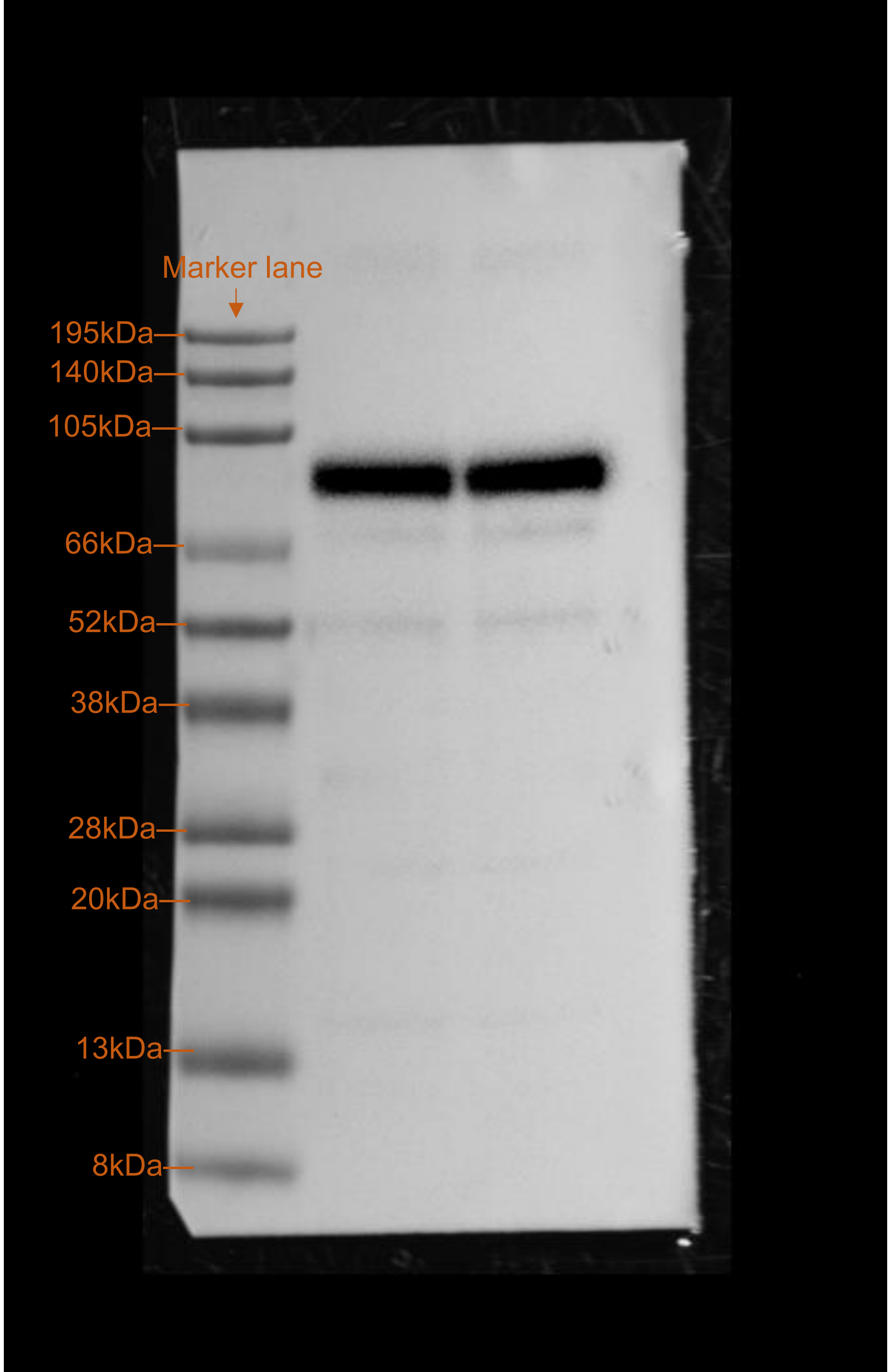

FOXO3 97kD

Figure 6G

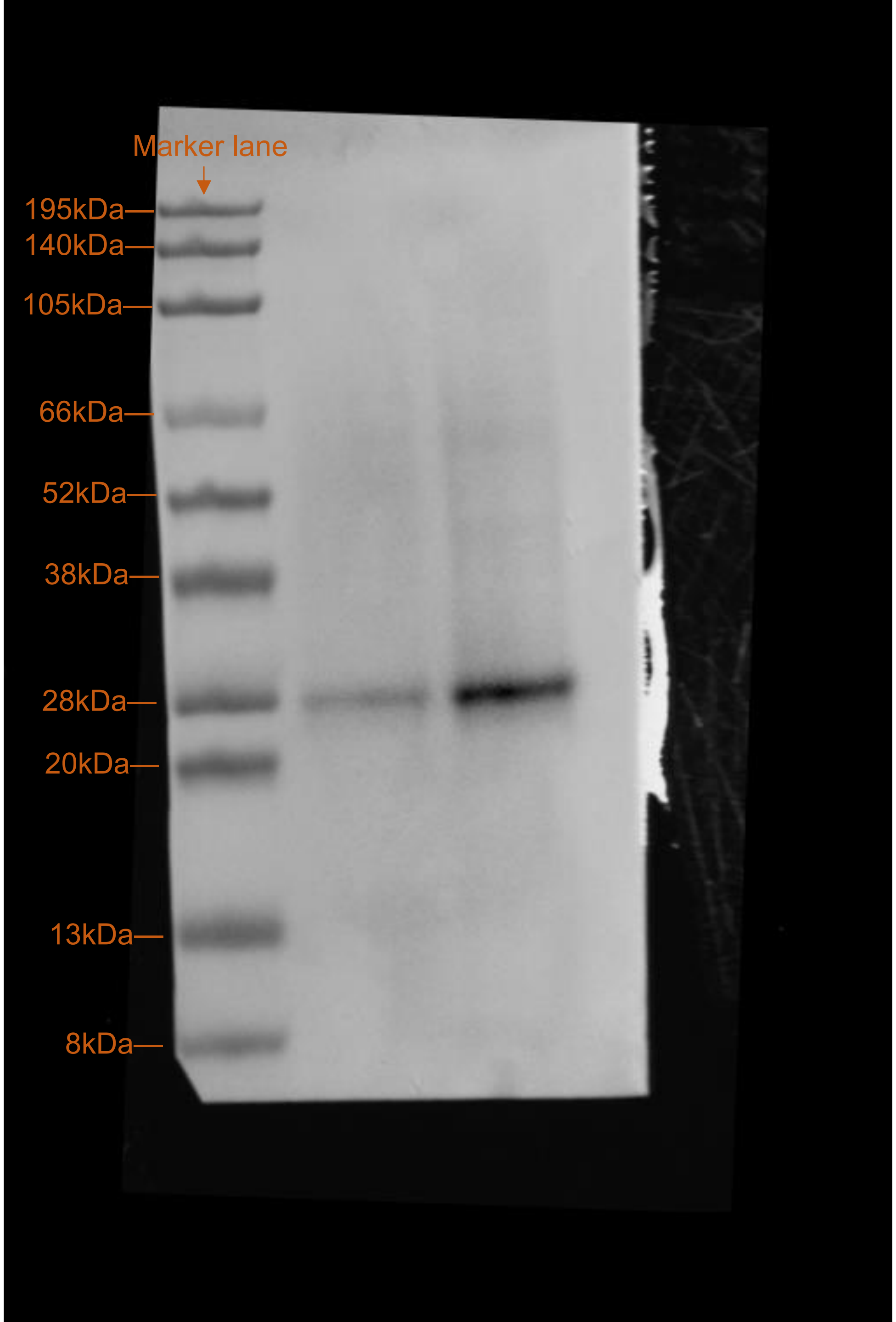

TFAM 29kD

Figure 6G

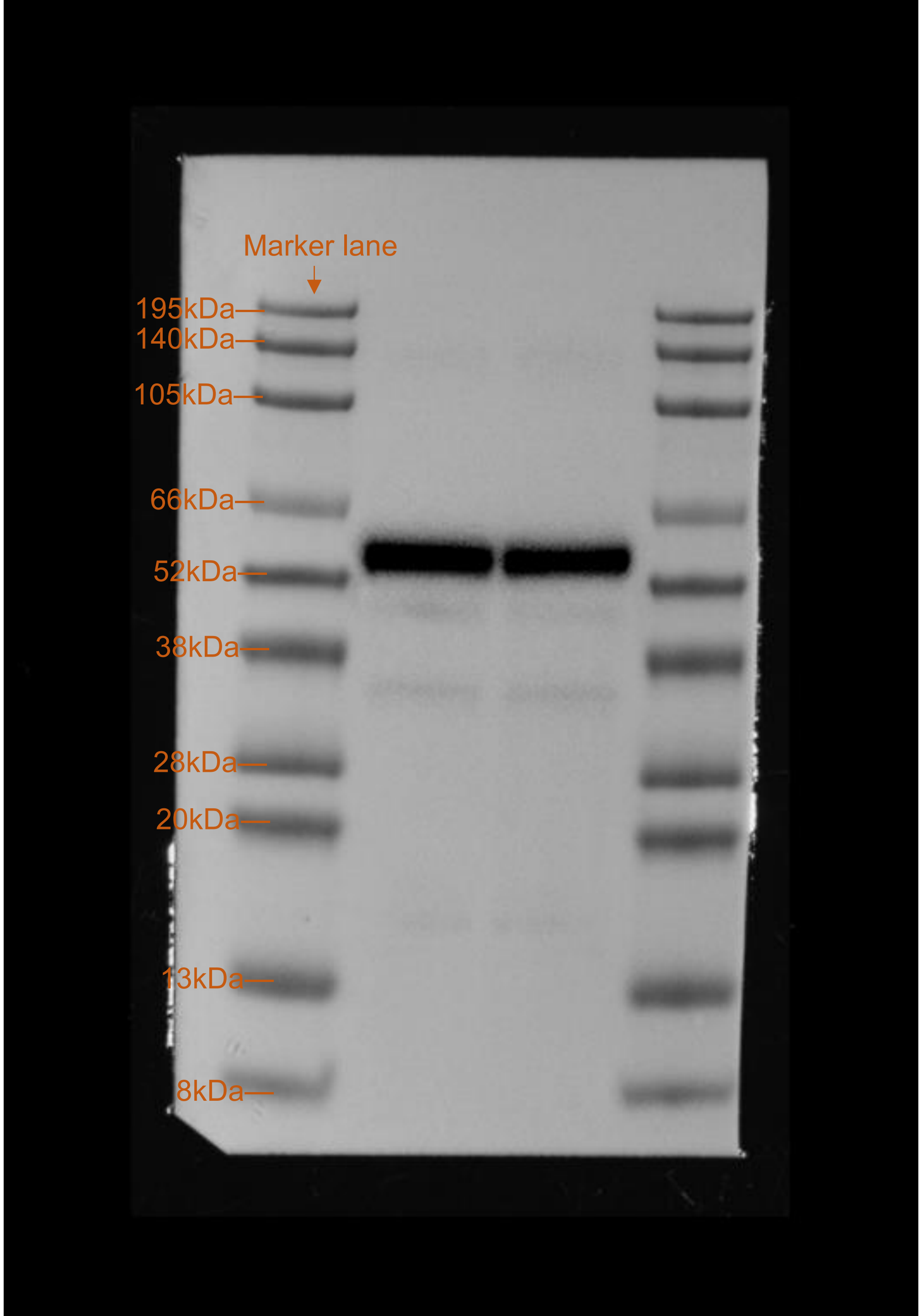

β-tubulin 55kD

Figure 7A

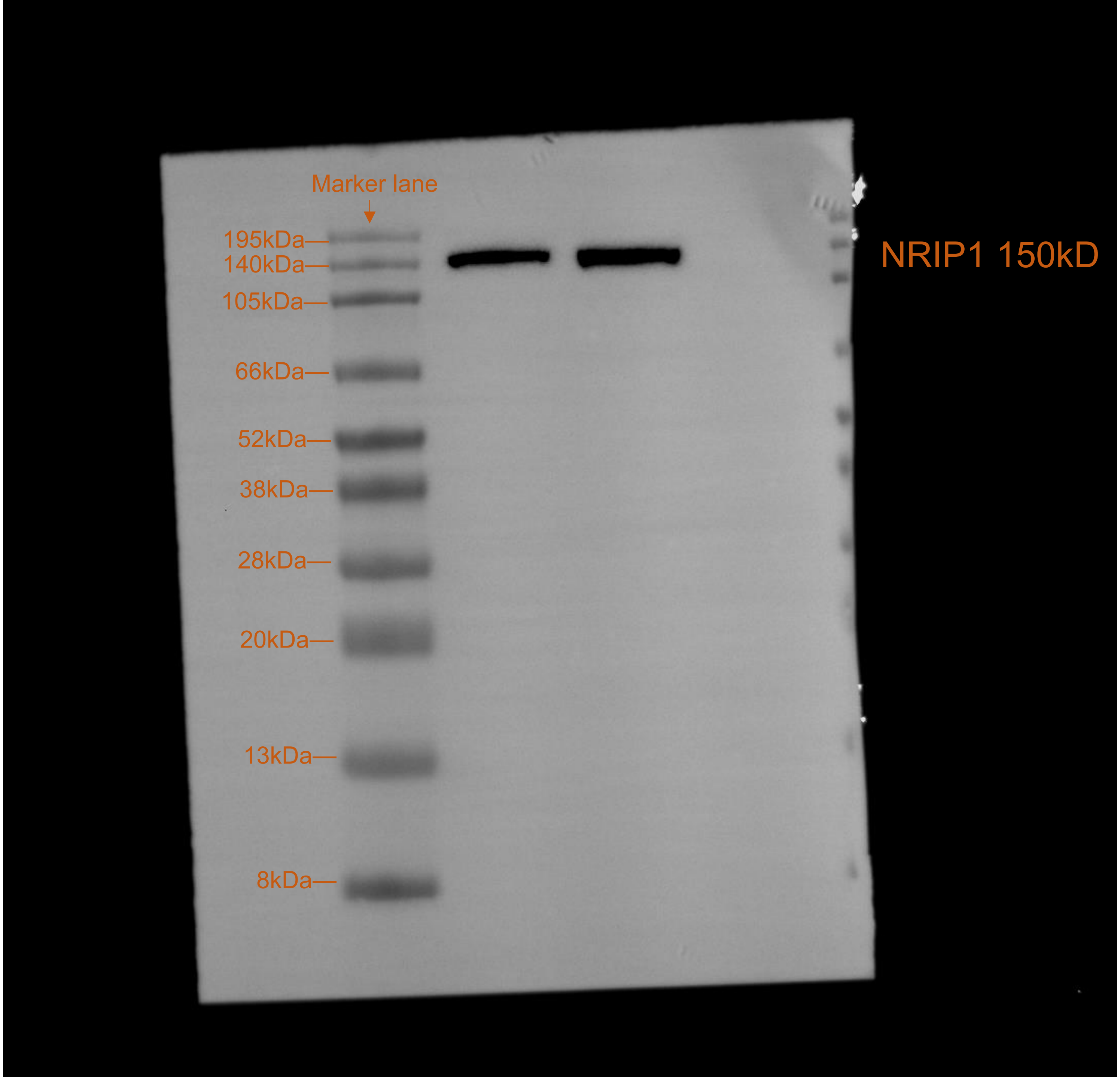

Marker lane
195kDa
140kDa
105kDa
66kDa
52kDa
38kDa
28kDa
20kDa
13kDa
8kDa
NRIP1 150kD

Figure 7A

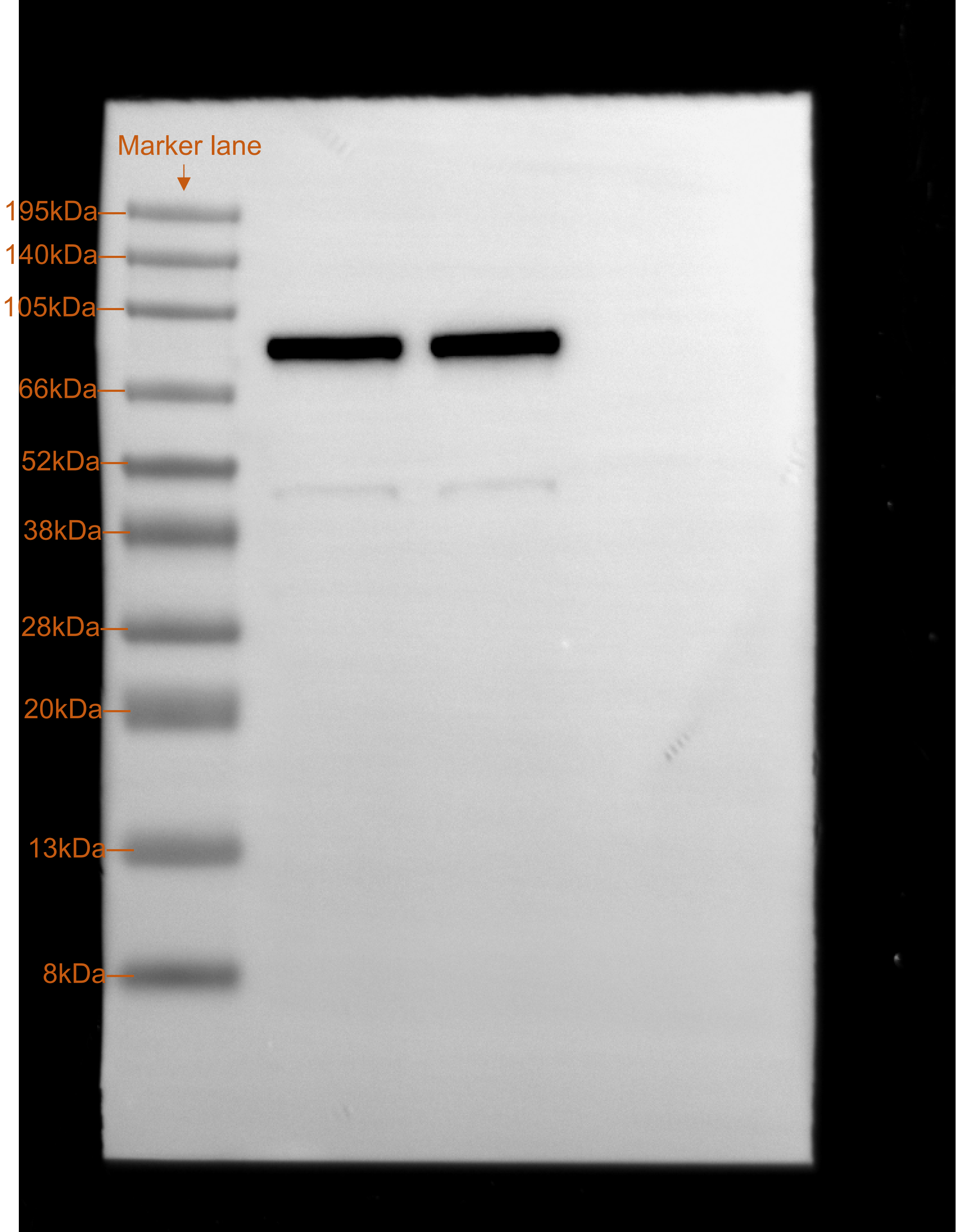

FOXO3 97kD

Figure 7A

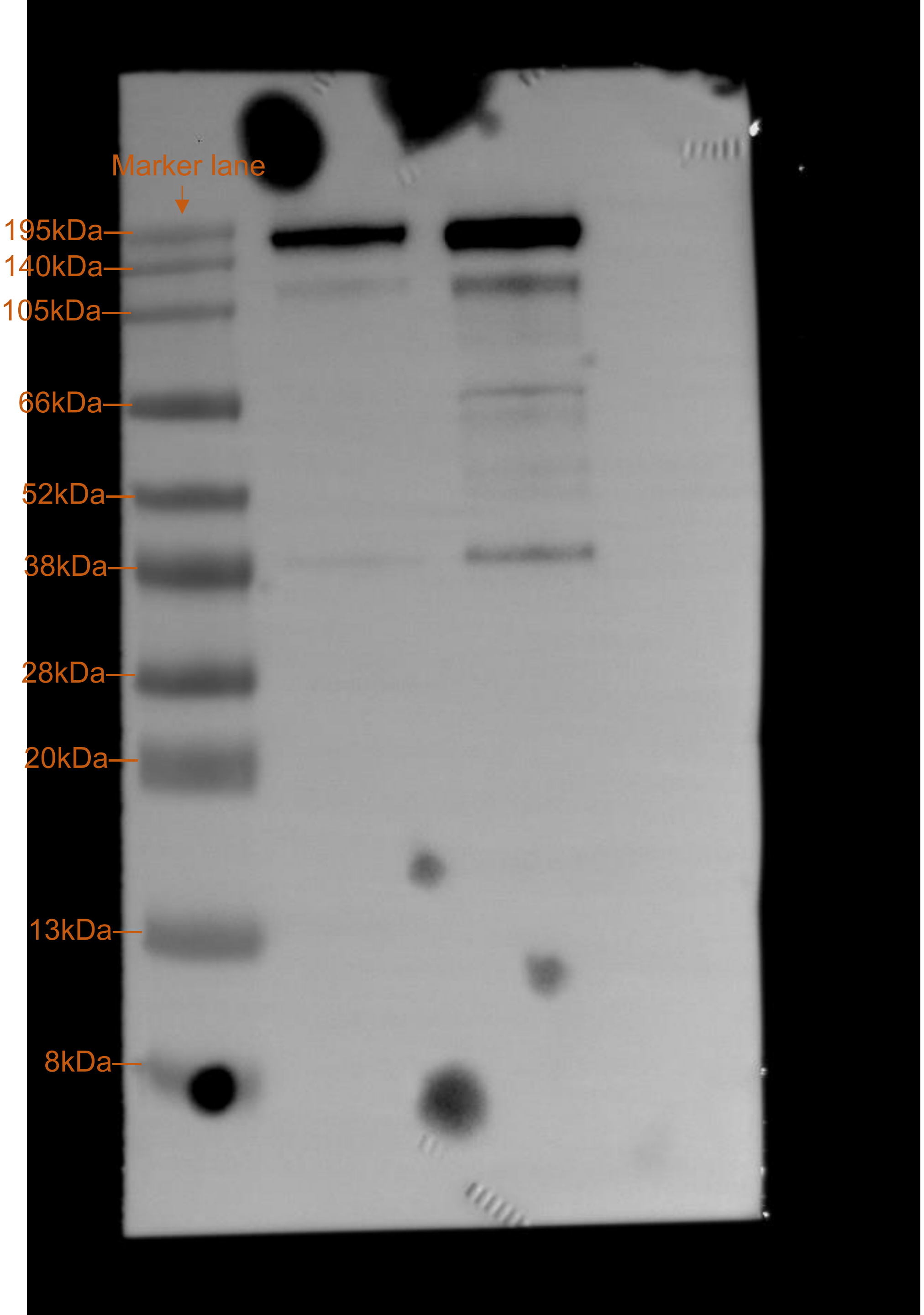

NRIP1 150kD

Figure 7A

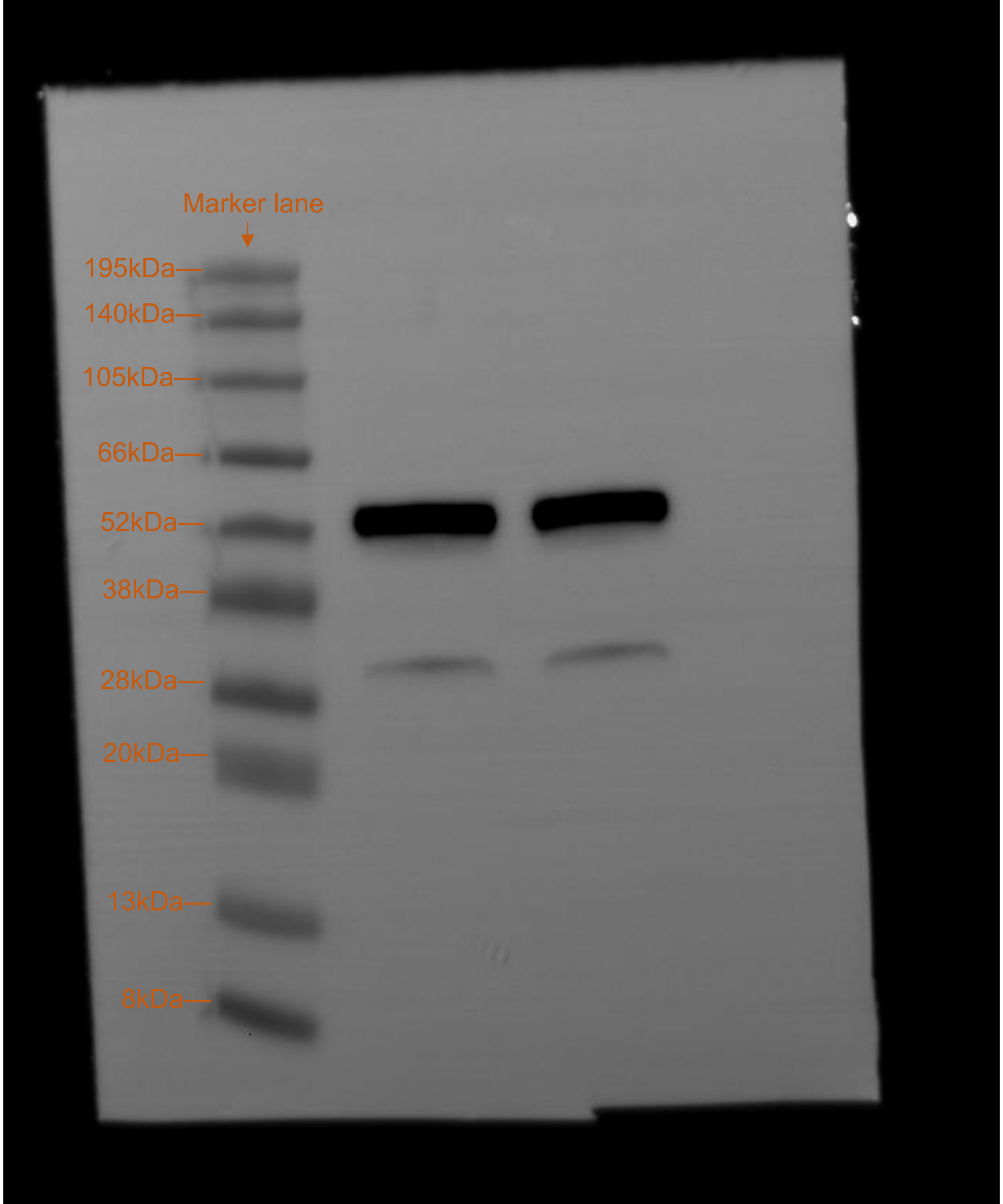

β-tubulin 55kD

Figure 7C

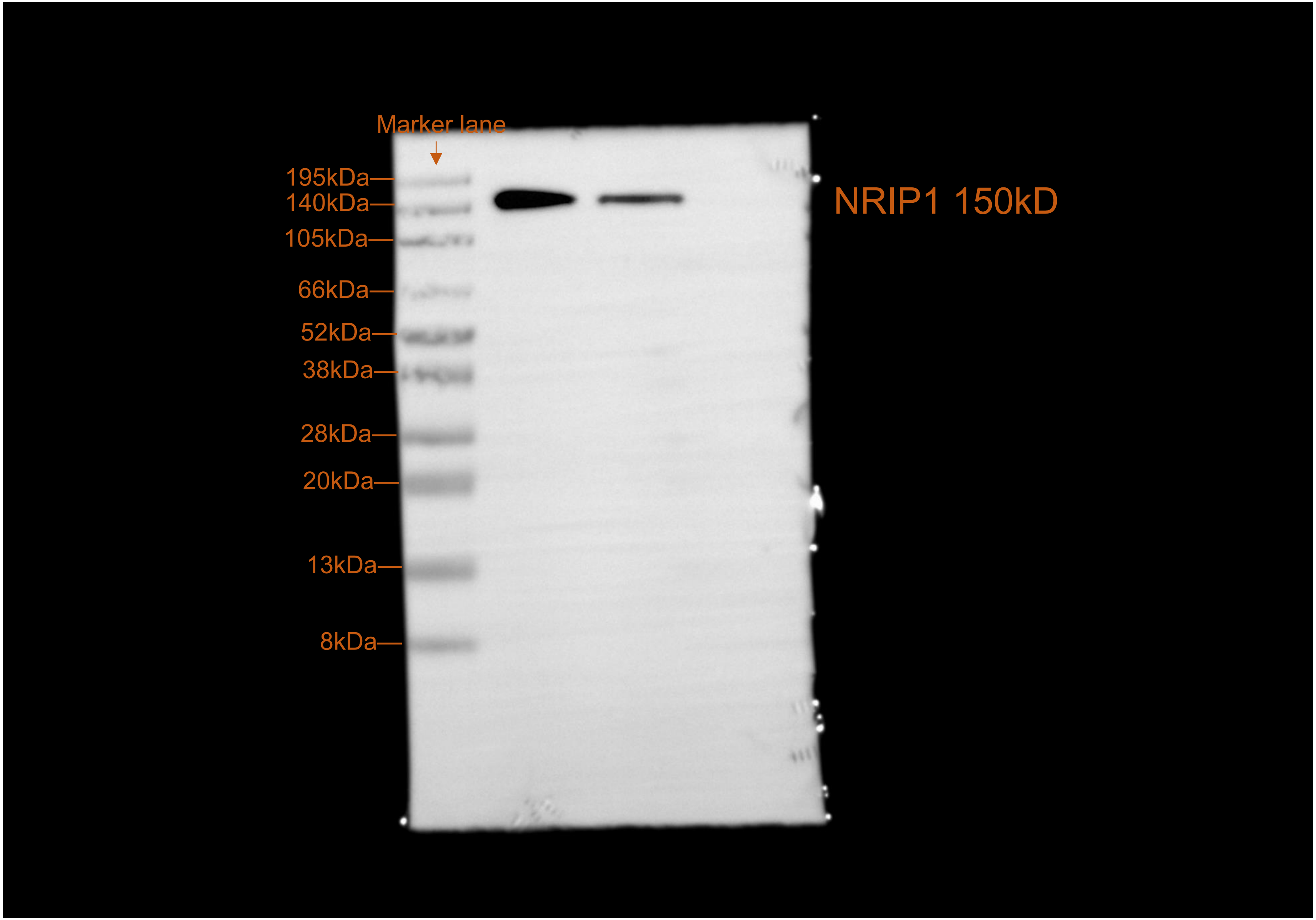

Marker lane
195kDa
140kDa
105kDa
66kDa
52kDa
38kDa
28kDa
20kDa
13kDa
8kDa
NRIP1 150kD

Figure 7C

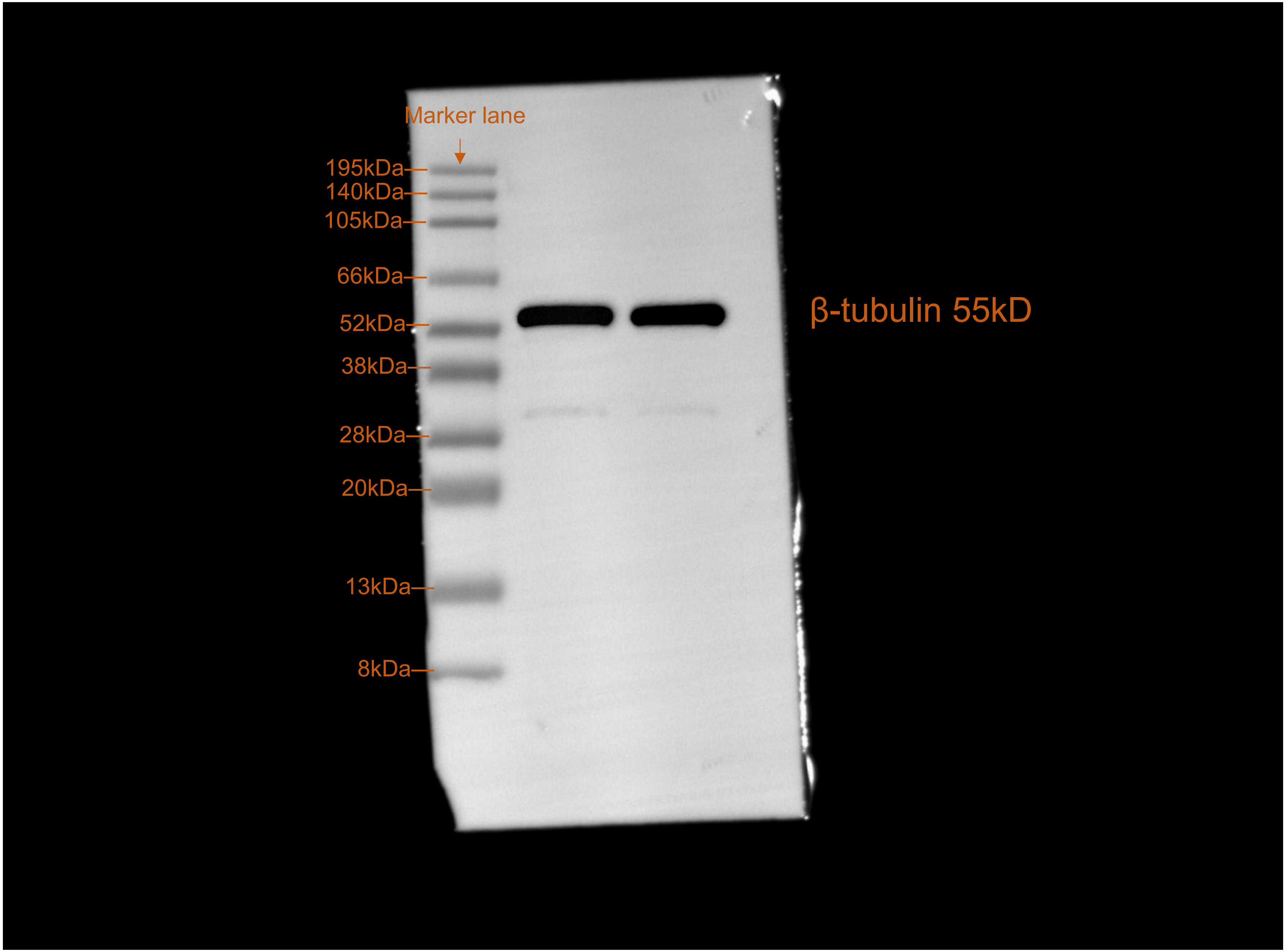
Marker lane
195kDa
140kDa
105kDa
66kDa
52kDa
38kDa
28kDa
20kDa
13kDa
8kDa
β-tubulin 55kD

Figure 7F

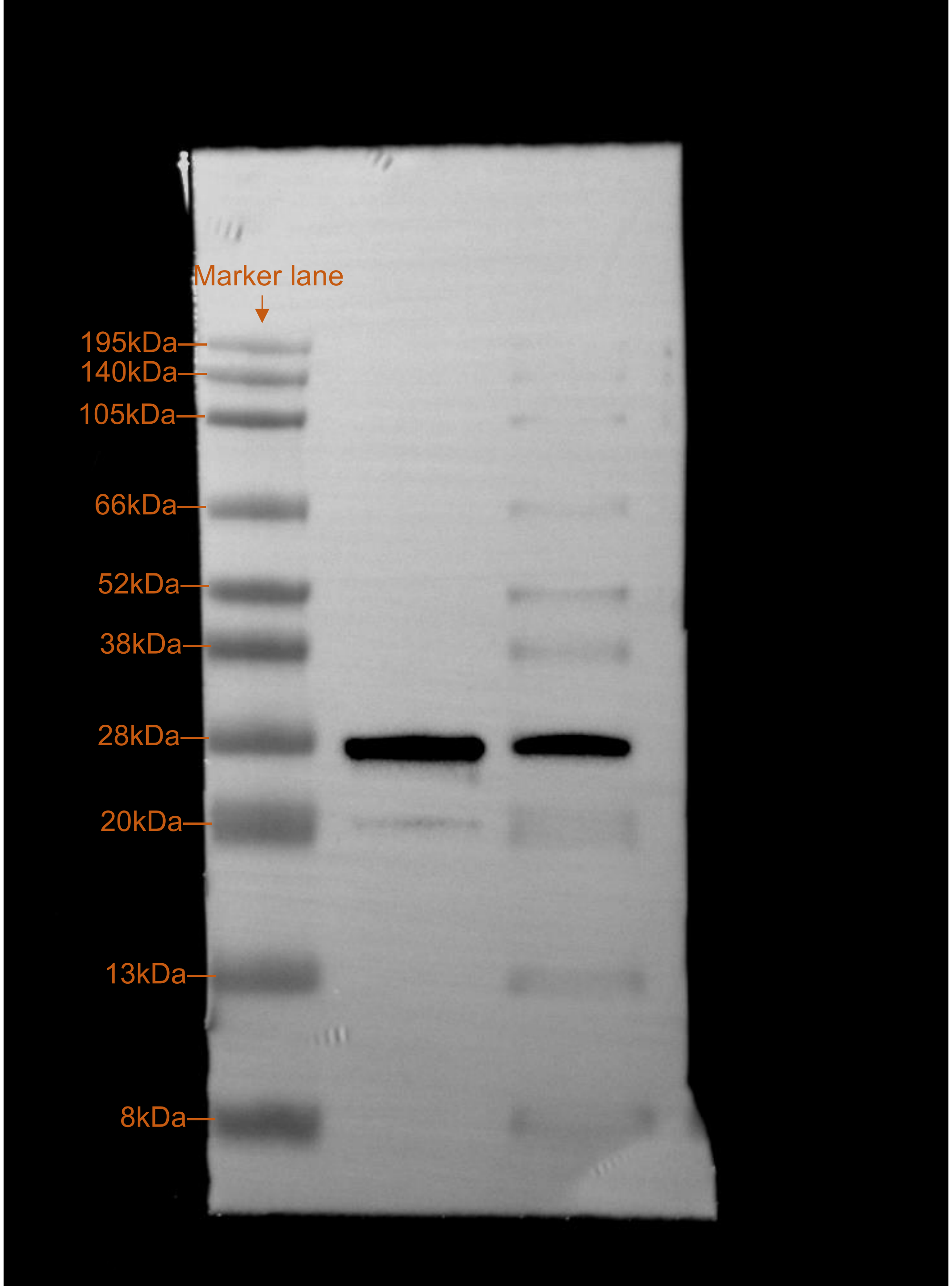

TFAM 29kD

Figure 7F

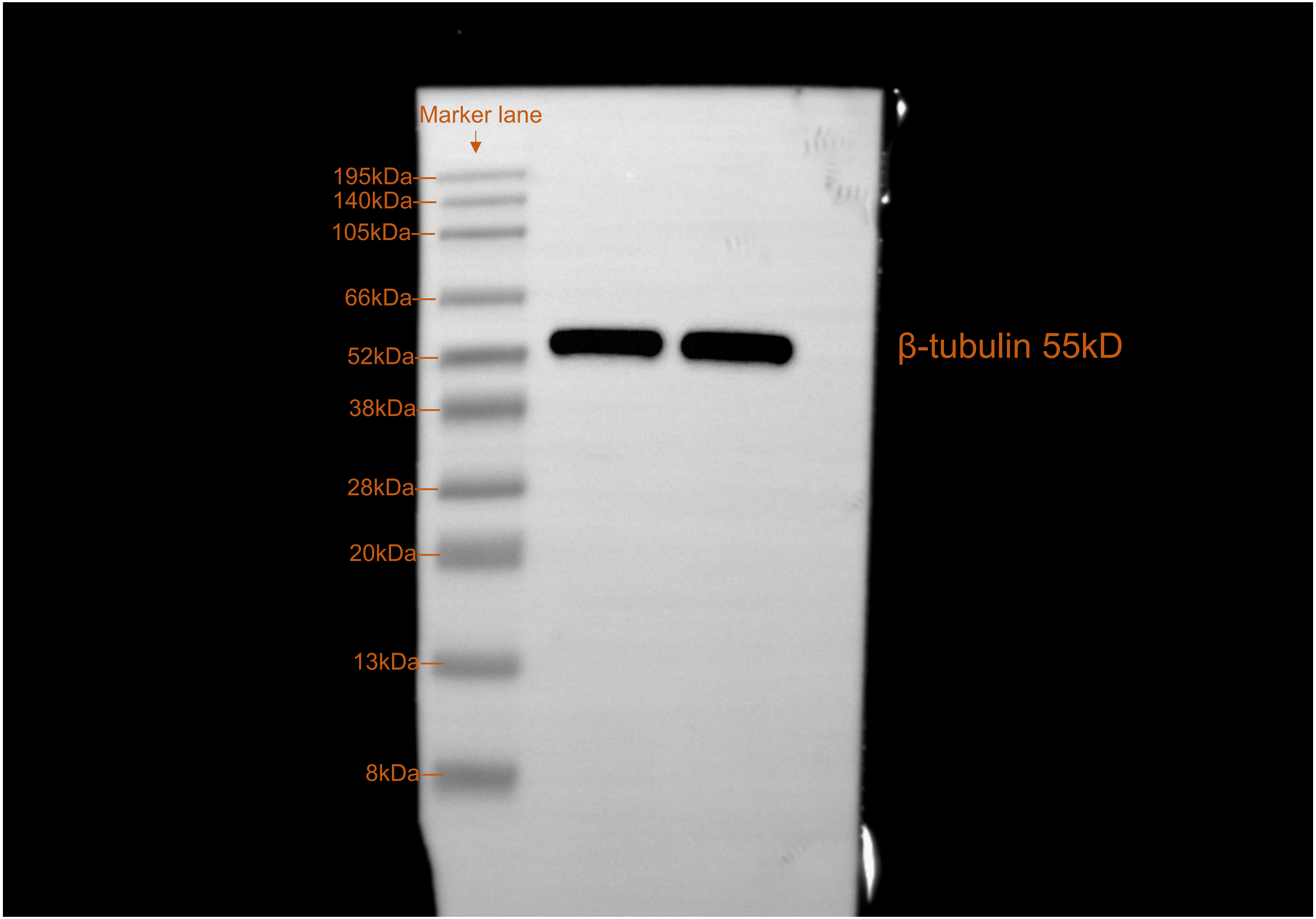
Marker lane
195kDa
140kDa
105kDa
66kDa
52kDa
38kDa
28kDa
20kDa
13kDa
8kDa
β-tubulin 55kD

Figure 7F

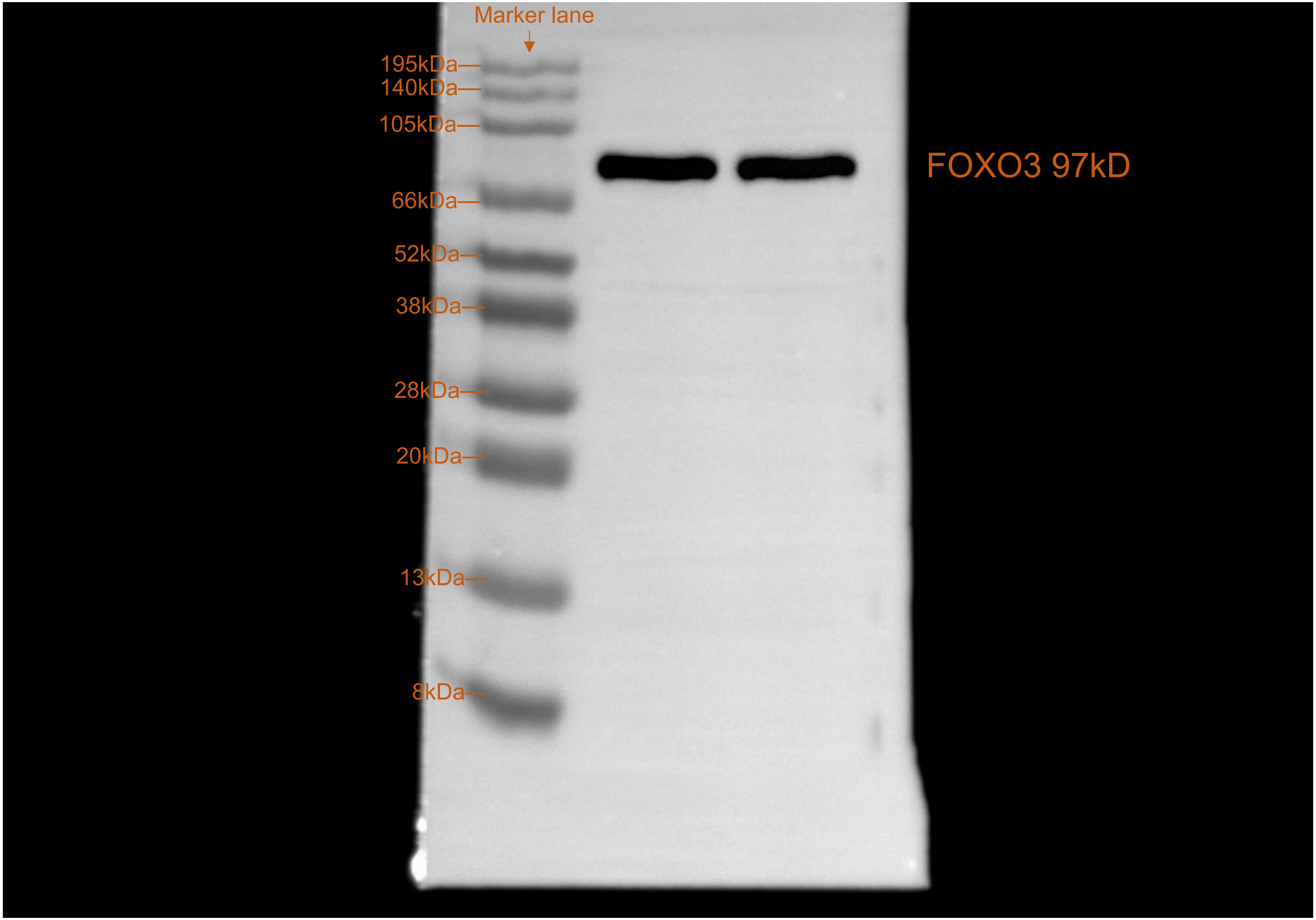

Marker lane
195kDa
140kDa
105kDa
66kDa
52kDa
38kDa
28kDa
20kDa
13kDa
8kDa
FOXO3 97kD

Figure 7F

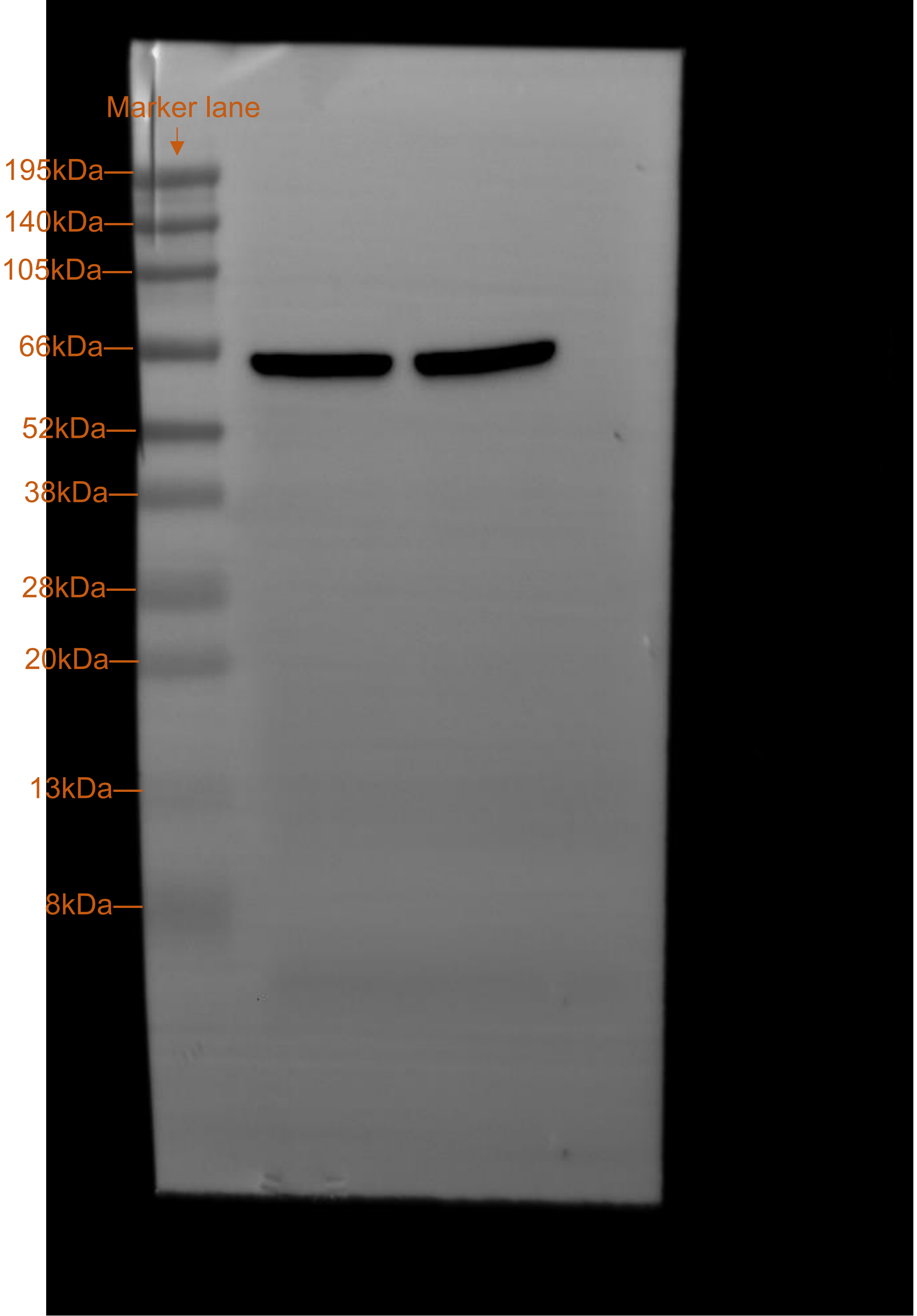

Lamin B1 66kD

Figure 7O

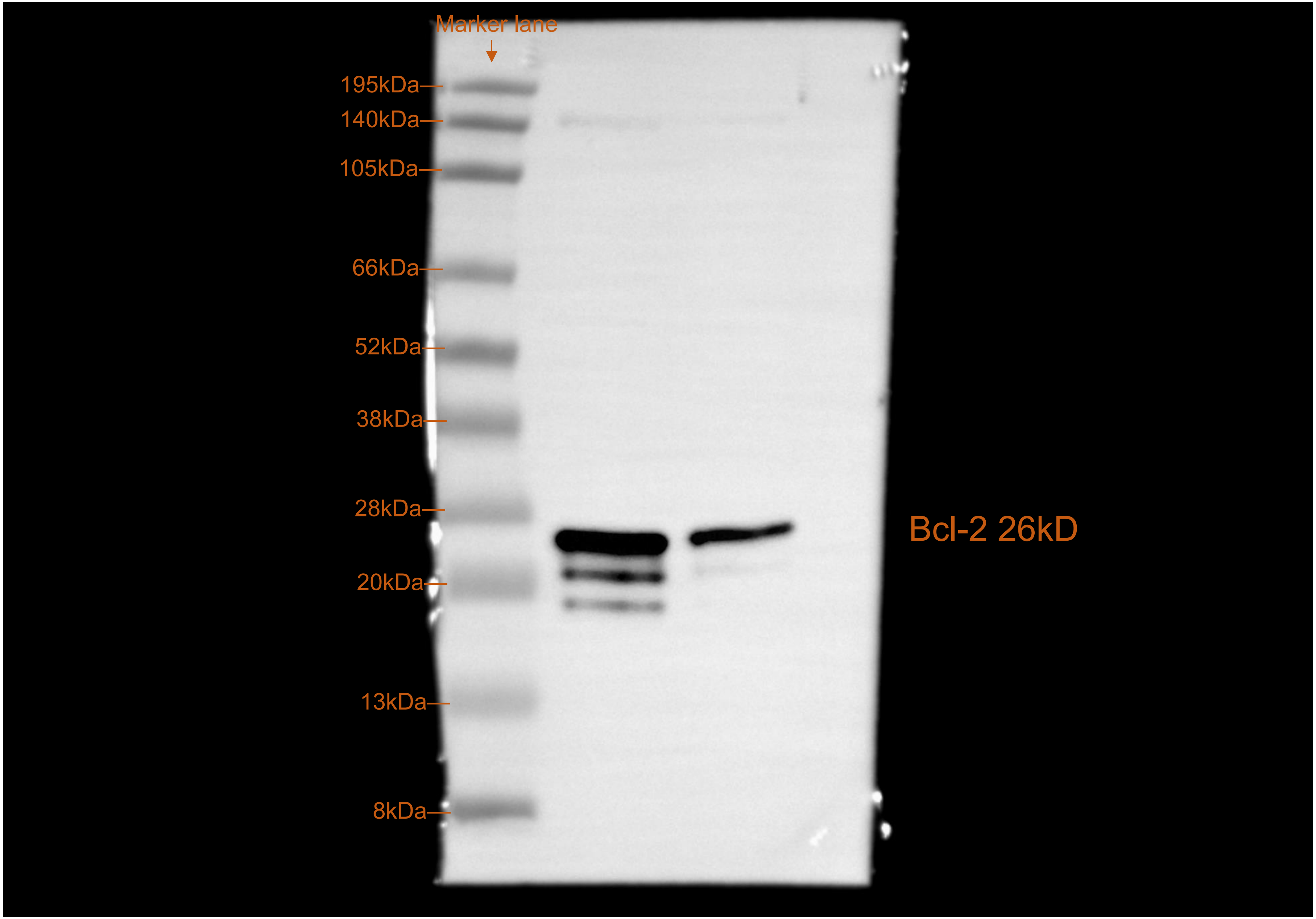
Marker lane
195kDa
140kDa
105kDa
66kDa
52kDa
38kDa
28kDa
20kDa
13kDa
8kDa
Bcl-2 26kD

Figure 7O

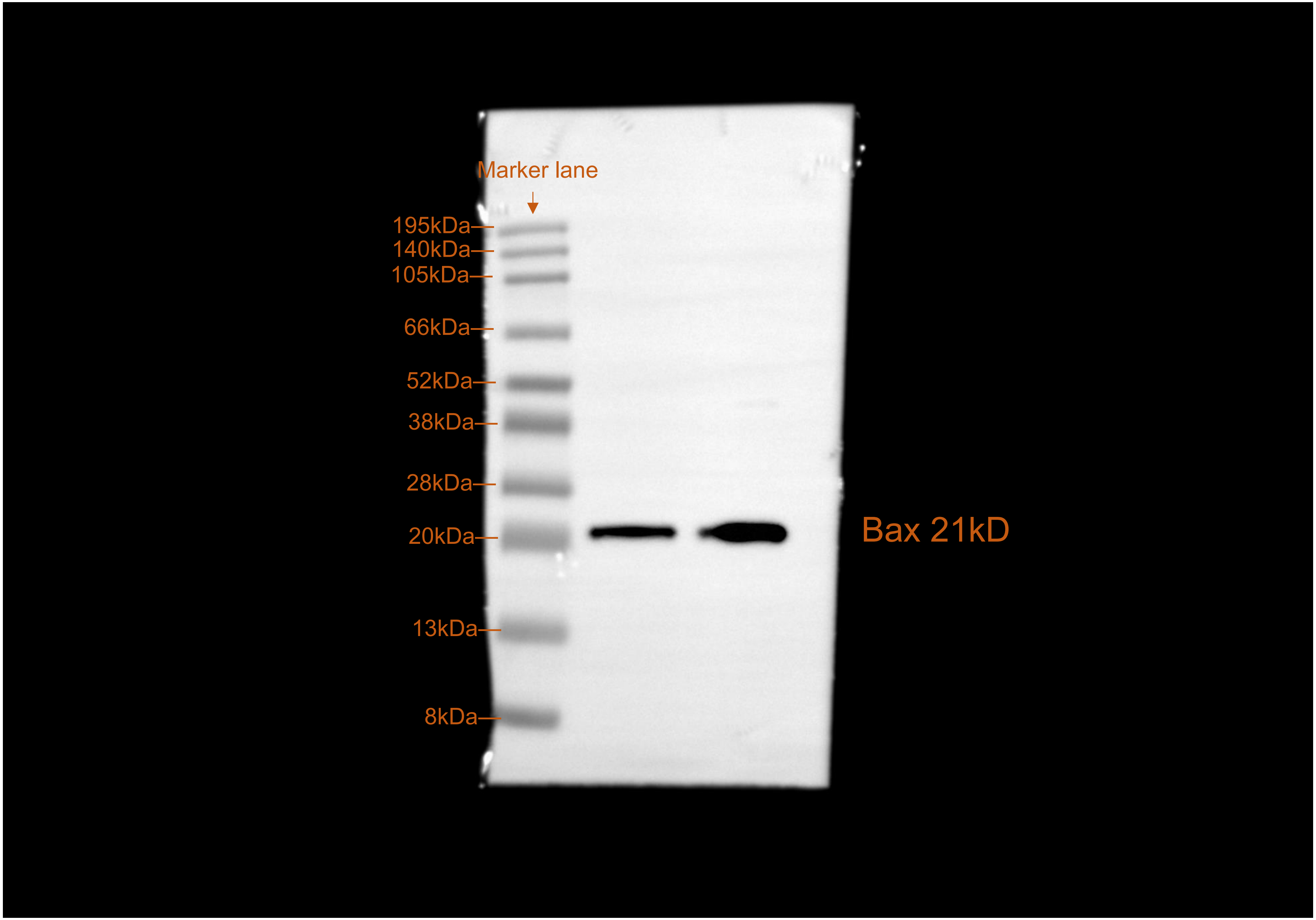

Marker lane
195kDa
140kDa
105kDa
66kDa
52kDa
38kDa
28kDa
20kDa
13kDa
8kDa
Bax 21kD

Figure 7O

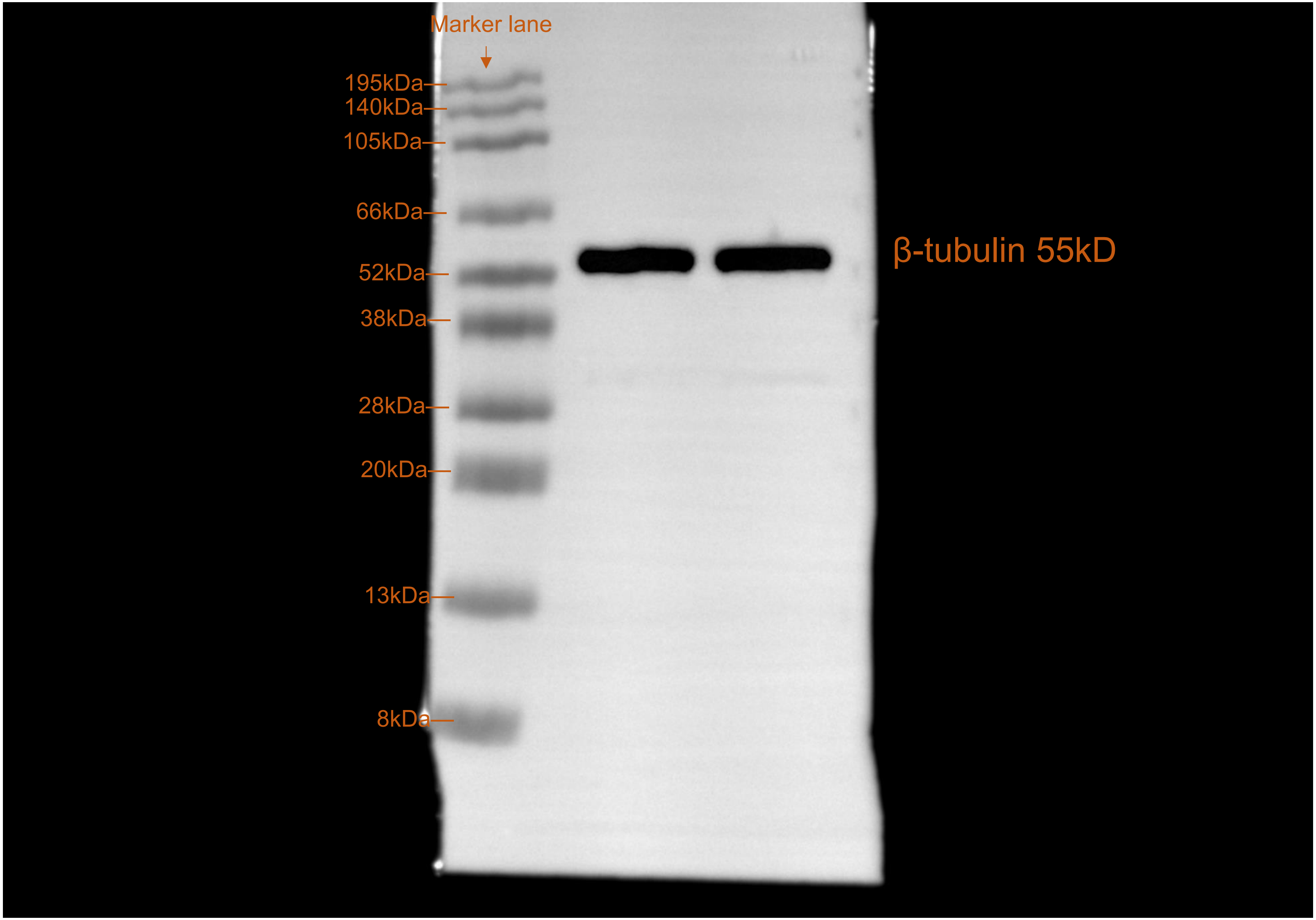

Marker lane
195kDa
140kDa
105kDa
66kDa
52kDa
38kDa
28kDa
20kDa
13kDa
8kDa
β-tubulin 55kD

Figure 8N

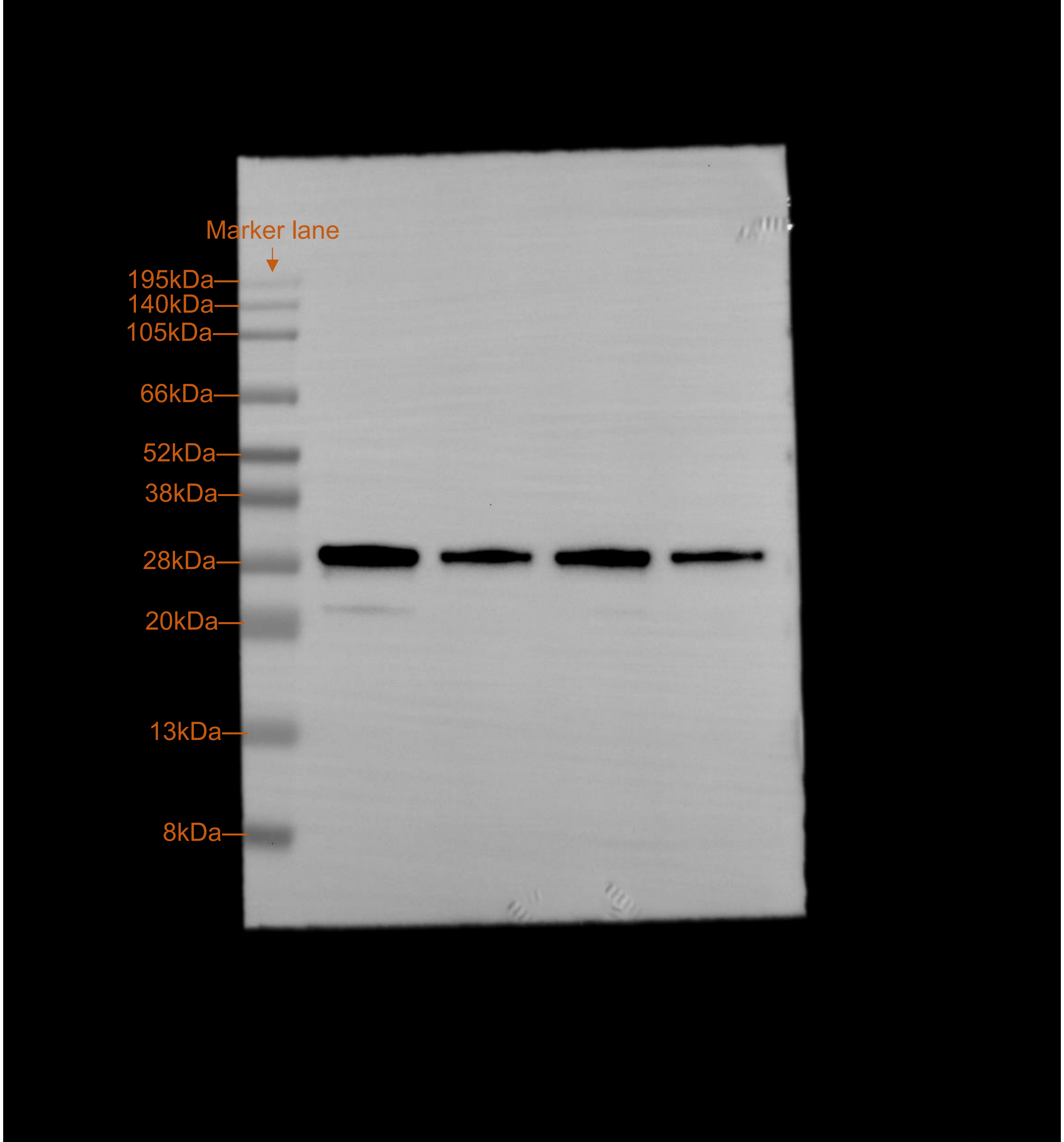

TFAM 29kD

Figure 8N

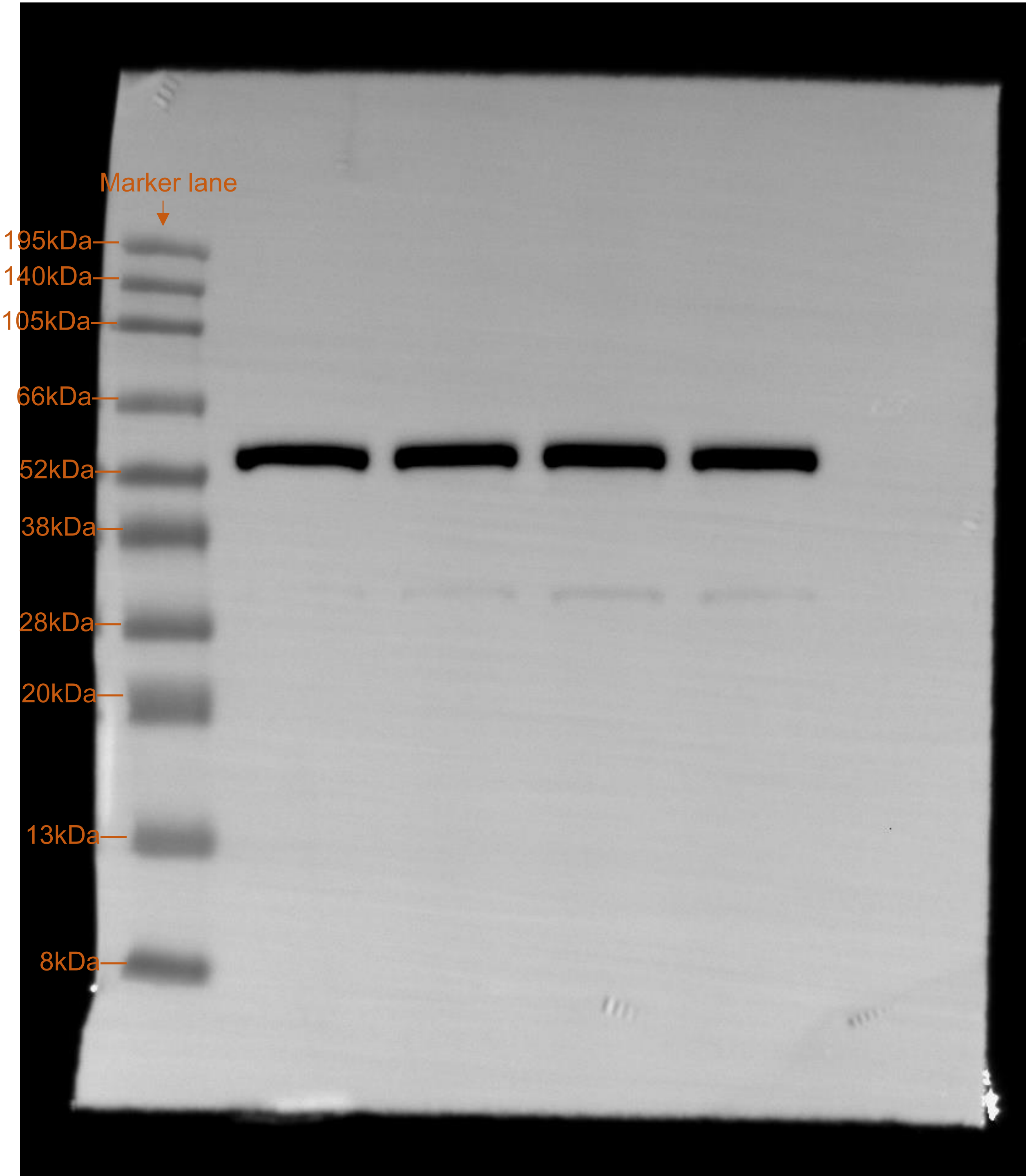

β-tubulin 55kD
